# Dietary sugar intake, genetic susceptibility, and risk of dementia: A prospective cohort study

**DOI:** 10.1016/j.tjpad.2025.100312

**Published:** 2025-07-26

**Authors:** Yu An, Limin Cao, Gang Zheng, Yashu Liu, Honghao Yang, Liangkai Chen, Yuhong Zhao, Xiaopeng Zhang, Yang Xia

**Affiliations:** a Medical Research Center, Beijing Institute of Respiratory Medicine and Beijing Chao-Yang Hospital, Capital Medical University, 100020, Beijing, China; b The Third Central Hospital of Tianjin, No. 83, Jin Tang Road, Tianjin, 300070, China; c Department of Clinical Epidemiology, Shengjing Hospital of China Medical University, Shenyang, No. 36, San Hao Street, Shenyang, Liaoning, 110004, China; d Liaoning Key Laboratory of Precision Medical Research on Major Chronic Disease, Shenyang, No. 36, San Hao Street, Shenyang, Liaoning, 110004, China; e Department of General Surgery, Shengjing Hospital of China Medical University, Shenyang, No. 36, San Hao Street, Shenyang, Liaoning, 110004, China; f Department of Nutrition and Food Hygiene, Hubei Key Laboratory of Food Nutrition and Safety, School of Public Health, Tongji Medical College, Huazhong University of Science and Technology, No. 13, Hang Kong Road, 430030, Wuhan, China; g Key Laboratory of Carcinogenesis and Translational Research (Ministry of Education/Beijing), Gastrointestinal Cancer Center, Peking University Cancer Hospital and Institute, Beijing 100142, China; h Department of Data Center, Shengjing Hospital of China Medical University, Shenyang, No. 36, San Hao Street, Shenyang, Liaoning, 110004, China.

**Keywords:** Dementia, Non-free sugar intake, Genetic susceptibility, Gut microbiota, Cohort

## Abstract

**Background:**

Sugar intake has been identified as a risk factor for incident dementia; however, the role of genetic susceptibility in such association remains unclear.

**Methods:**

This cohort study involved 158,408 participants from the UK Biobank to explore the effect of genetic susceptibility on the association between dietary sugar intake and dementia risk. Data on sugar intake were evaluated using repeated web-based 24-hour dietary recalls. Polygenic risk scores (PRS) for sugar metabolism (Triglyceride Glucose, TyG), gut microbiota, and disease susceptibility (Alzheimer's disease) were generated based on genome-wide association studies.

**Results:**

Over a median follow-up period of 9.94 years, 1,219 dementia cases (0.7%) were documented. There were significant positive dose-response relationships between sugar intake and dementia risk (non-free sugar: HR, 95% CI, _Quartile 4_ vs. _Quartile 1_ = 1.26, 1.04–1.52; free sugar: 1.43, 1.20–1.70). Genetic susceptibility, including TyG-PRS, gut microbiota, and disease susceptibility, showed a combined effect on the association between sugar intake and dementia risk. Notably, significant interactions were observed between sugar intake, PRS for *Ruminococcaceae UCG-014* and dementia, as well as between free sugar, PRS for *Oscillospira* and dementia. Participants with lower PRS of *Ruminococcaceae UCG-014*, or higher PRS of *Oscillospira*, posed a higher risk of dementia due to sugar intake.

**Conclusion:**

Both free and non-free sugar intake are independent risk factors for dementia incidence. The role of genetic susceptibility among such association cannot be ignored. These results underscore the importance of personalized nutritional interventions targeting both dietary habits and genetic risk profiles in dementia prevention strategies.

## 1 Background

Dementia poses a great global health challenge, currently affecting 55 million individuals with approximately 10 million new cases annually [1]. By 2050, this number is projected to rise to 152 million [2]. With the aging population, the prevalence of dementia is expected to double approximately every five years between 2019 and 2050, particularly among individuals aged 85 years and above [2], making it one of the leading contributors of disability and dependency among older adults [3]. Despite recent advances in the treatment of Alzheimer's disease (AD) showing clinical potential [4], primary prevention through dietary regulation remains critical [5].

Excessive intake of sugar, particularly free sugar (all monosaccharides and disaccharides added to foods by manufacturers, cooks, or consumers, as well as sugars occurring naturally in honey, syrups, and unsweetened fruit juices), has been linked to an increased incidence of noncommunicable diseases such as obesity, diabetes [6], and neurodegenerative disorders [[7], [8], [9], [10]]. Moreover, intake of non-free sugar has also been reported to have adverse effects on dementia [10], despite its demonstrated benefits for cardiovascular diseases (CVDs) [11] and reduction of liver fat [12]. Furthermore, the gut microbiota-brain axis (GMBA) has emerged as a potential target for modulating brain health [13]. The "gut microbiome" is the largest repository of microbes in the human body, containing about 10^14^ species [14]. These gut microbes play important roles in physiological homeostasis and metabolism, promoting the development of the immune system, vitamin production, and nutrient absorption, and have been demonstrated to be involved in the onset of neurodegenerative diseases (such as glucose and fat metabolism and insulin sensitivity) [15,16].

On the other hand, it has been widely accepted that dementia develops at the complex intersection of environmental factors and inherited predisposition [17]. Research from Genome-Wide Association Studies (GWASs) has recently identified a growing number of genetic variations associated with the risk of dementia, with heritability estimates spanning 13 %−80 % across different populations [18]. Notably, emerging evidence has demonstrated that a favorable lifestyle is associated with a decreased risk of dementia among individuals with high genetic risk [19]. Therefore, exploring modifiable risk factors for dementia needs to consider an individual’s inherent genetic susceptibility. However, limited studies reported the role of disease susceptibility among the association between sugar intake and dementia risk. What’s more, the genetic susceptibility of sugar metabolism and gut microbiota may also influence the risk of dementia, along with sugar intake.

To address these knowledge gaps, the current study aimed to prospectively explore the long-term associations of both non-free sugar and free sugar intake with dementia risk, and furtherly investigated whether genetic susceptibility of sugar metabolism, gut microbiota, and disease susceptibility play a role in such association based on the UK Biobank, a large-scale prospective cohort study involving approximately half million participants.

## 2 Methods

### 2.1 Study design and population

The UK Biobank is an extensive cohort involving over 500,000 individuals aged 37–73 years from across England, Scotland, and Wales. The detailed information about the structure and demographics of the UK Biobank can be found in previous studies [20,21].

**Fig 1:**
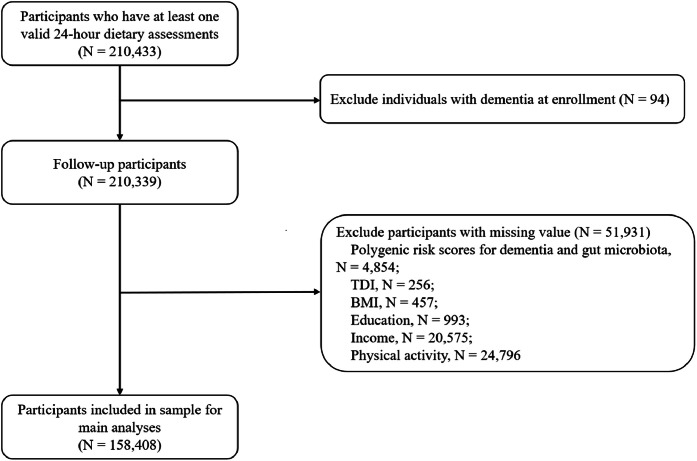
Flowchart for the selection of the study population. *Abbreviations:* BMI, body mass index; TDI, Townsend Deprivation Index.

In the current cohort study, a dietary questionnaire with credible energy intake (defined as >0–20 MJ/day for males, >0–18 MJ/day for females) was considered valid [22]. The baseline was defined as the time when participants completed the first valid 24-hour recall dietary questionnaire. Among 210,433 participants who had at least one valid 24-hour recall dietary assessment, we excluded (1) those who had dementia at baseline (*n* = 94) and (2) those with missing data on covariates (*n* = 51,931). Finally, 158,408 participants remained in the final analysis (Fig 1).

### 2.2 Assessment of sugar intake

The Oxford WebQ questionnaire, an online tool for assessing dietary intake over a 24-hour period, was utilized to collect dietary information [23]. The initial round was conducted for participants recruited between 2009 and 2010 at assessment centers. Individuals who submitted a verifiable email address during the recruitment process were subsequently extended an invitation to partake in 24-hour dietary evaluations spanning from February 2011 to April 2012. The intake of non-free sugar, free sugar, and other nutrients was derived from the average of ≥ one (maximum of five) 24-hour dietary assessments, calculated by multiplying the content of food items and beverages by the frequency of intake using UK Nutrient Databank food composition tables [23]. The primary exposure of interest was the percentage of total energy intake from non-free sugar and free sugar. For further analyses, the percentages were categorized into quartiles.

### 2.3 Assessment of dementia

Dementia cases were identified by the Hospital Episode Statistics for England, Scottish Morbidity Records for Scotland, and Patient Episode Database for Wales. The incident and prevalent dementia cases were determined by utilizing International Classification of Diseases, 10th Revision (ICD-10) (F00, F000, F001, F002, F009, G30, G300, G301, G308, G309, F01, F010, F011, F012, F013, F018, F019, I673, F020, G310, A810, F02, F021, F022, F023, F024, F028, F03, F051, F106, G311, G318), present in hospital inpatient data and death registry records. The identification of dementia-related deaths was accomplished by linking the data to the death registry. The follow-up time was analyzed starting at the baseline date and ending at the dementia diagnosis, censoring date, or death (up to May 2021), whichever came first.

### 2.4 The polygenic risk score (PRS) for sugar metabolism and gut microbiota

The triglyceride-glucose (TyG) index represents a robust surrogate marker for evaluating insulin resistance (IR) and overall glucose metabolism, calculated through the logarithmic transformation of fasting triglyceride (TG) and fasting plasma glucose (FPG) concentrations. This composite biomarker has demonstrated significant clinical utility in predicting the development of metabolic dysregulation and associated pathological consequences. Thus, we built a weighted polygenic risk score as an instrumental variable for TyG using independent genetic variants (linkage disequilibrium R^2^ < 0.2 and a 500 kb clustering window) with *P* < 5 × 10^−8^ at a genome-wide level in large numbers of individuals from European-descent populations [24]. Overall, 192 genetic variants were selected, and 21 SNPs were excluded due to a high R^2^. The remaining 171 SNPs were all independent of each other (i.e. linkage equilibrium) and were used to construct the PRS. The PRS for each participant was calculated by summing the number of risk alleles and was weighted by the corresponding effect size (*β*-coefficients) derived from the GWAS study. The following equation was applied: $PRS=\beta_{1}\times SNP_{1}+\beta_{2}\times SNP_{2}+\cdots +\beta_{n}\times SNP_{n}$ [25]. The characteristics of the SNPs associated with sugar metabolism were listed in **Supplementary Table 1**.

The PRS for gut microbiota was derived from the largest GWAS meta-analysis published to date conducted by the MiBioGen consortium [26]. The study coordinated 16S rRNA gene sequencing profiles and genotyping data from 24 cohorts. Most participants were of European ancestry (*N* = 13,266). Among them, the taxonomic level of genus was the lowest; 131 genera (including 12 unknown genera) were identified, and the average abundance was more than 1 %. We built weighted PRS for genus-level gut microbes using independent genetic variants (linkage disequilibrium R^2^ < 0.001 and a 10,000 kb clustering window) with *P* < 1 × 10^–5^ at a genome-wide level in large numbers of individuals from European-descent populations. Single-nucleotide polymorphisms (SNPs) with a minor allele frequency of < 0.01, ambiguous SNPs with non-concordant alleles, and palindromic SNPs were excluded. The remaining SNPs were all independent of each other (i.e., linkage equilibrium) and were used to construct the PRS. The PRS for each participant was calculated by summing the number of risk alleles and was weighted by the corresponding effect size (β-coefficients) derived from the GWAS study. The following equation was applied: $PRS=\beta_{1}\times SNP_{1}+\beta_{2}\times SNP_{2}+\cdots +\beta_{n}\times SNP_{n}$ [25]. The characteristics of the SNPs associated with genus-level gut microbes were listed in **Supplementary Table 2**.

### 2.5 Assessment of covariates

Potential confounding variables were evaluated using the baseline questionnaire, which included information on comorbidities, demographics, physical, lifestyle characteristics, and family history of dementia. The identification of previous occurrences of comorbidities, including cardiovascular disease, diabetes, and hypertension, was based on either the self-reporting of medical conditions or the utilization of ICD-10 codes in hospital inpatient records. Standardized techniques were employed to gather physical measurements and biological specimens, and these procedures have been previously described and validated [27]. Demographic, physical, and lifestyle characteristics, including age, sex, race, education level, average total household income before tax, Townsend Deprivation Index (TDI), smoking status, alcohol drinking status, and family history of dementia, were all collected by self-administered touchscreen survey and interview at recruitment. Physical activity was evaluated in metabolic equivalent (MET) minutes per week for all activities using a short-form international physical activity questionnaire [28]. Anthropometric indices (e.g., height and weight) were calculated by trained and experienced staff during the initial visit at the assessment center. Body mass index (BMI) was calculated by weight in kilograms divided by the square of height in meters.

### 2.6 Statistical analyses

The baseline characteristics of participants were described by the highest and lowest quartiles of the percentage of energy intake of non-free sugar and free sugar. Continuous and categorical variables were expressed as means (standard deviations) and numbers (proportions), respectively. The cause-specific competing risk model was performed to determine the hazard ratios (HRs) and 95% confidence intervals (CIs) for the associations of non-free and free sugar intake with dementia risk, while treating deaths before dementia development as competing risks and censoring. The linear trend was tested by using the median values of each quartile for non-free and free sugar intake (per 5 % energy increase) as continuous values. Restricted cubic splines were performed to flexibly model the associations between non-free and free sugar intake (continuous variables) and dementia risk, and the minimum Akaike information criterion was used to choose optimal knots. The reference point was 10 % of total energy intake for non-free and free sugar following World Health Organization guidelines [29]. Three sets of models were fitted. Model 1 was the crude model. Model 2 was adjusted for age at recruitment (years), sex (male, female), race (white, non-white), education (college or university degree, high school, below), average total household income before tax (< £18,000, £18,000–£52,000, ≥ £52,000), TDI (continuous), physical activity (MET, continuous), tobacco intake status (never, previous, and current), alcohol intake status (never, previous, and current), protein and fat intake (percentage of energy intake), and total energy intake (kcal/day, continuous). Model 3 was additionally adjusted for variables in model 2, plus hypertension history (yes, no), diabetes history (yes, no), CVD history (yes, no), and family history of dementia (yes, no). Model 3 was also mutually adjusted for non-free sugar intake and free sugar intake.

To examine potential modifying effects, we additionally repeated analyses stratified by age (< 60 or ≥ 60 years), sex (male or female), BMI (< 25 or ≥ 25 kg/m^2^), physical activity (below or above the median value), history of diabetes (yes or no), and history of hypertension (yes or no). *P*-value for interactions between these stratifying factors and sugar intake were calculated by entering their multiplication terms in model 3. The robustness of our findings was examined in sensitivity analyses by (i) excluding dementia cases within the first 2 years of follow-up to reduce the possibility of reverse causation; (ii) restricting to participants who had completed ≥ 2 dietary assessments to minimize the effects of random error and within-person variability; (iii) using absolute intake of non-free and free sugar (g/day) as exposures; (iv) using the ratio dividing non-free sugar intake or free sugar intake by total carbohydrate intake (%) as exposures; and (v) using Fine and Gray’s model to estimate the sub-distribution HRs (95% CIs) of incident dementia with the intake of sugar.

Furthermore, we investigated the PRS of sugar metabolism, gut microbiota, and disease susceptibility of dementia in the association between sugar intake and incident dementia, respectively. To explore the role of microbiota abundance on the association of sugar intake and dementia risk, valid data were selected from 119 kinds of gut microbiota’s PRS with false discovery rate (FDR) estimation [30,31]. As reported elsewhere, we additionally considered the PRS of total relative abundance of gut microbial taxa [32] as a covariate in the sensitivity analysis. Finally, the modifying effects of the valid gut microbiota’s PRS on the association between sugar intake and dementia risk were assessed. To assess for modifying effects between the disease susceptibility and the intake of non-free and free sugars on dementia risk, the standard released version of PRS for AD from the UK-Biobank was used to evaluate the disease susceptibility. Detailed information on the PRS can be found elsewhere [33].

The analyses were stratified by PRS (TyG-PRS, gut microbial-PRS, or dementia-PRS) categories (high PRS: ≥ median value; low PRS: < median value), and the product term of non-free or free sugar with PRS was included in the multivariate-adjusted models. To further evaluate the joint associations of sugar (non-free or free) intake and genetic susceptibility, we classified participants into eight groups according to the categories of PRS and the quartiles of sugar intake. The HRs (95% CIs) of incident dementia in different groups were estimated and compared to those with low risk of PRS and in the lowest quartile of sugar intake. The HRs (95% CIs) were estimated using Model 3 and further adjusted for the first 10 genetic principal components and the genotyping batch.

All statistical analyses were conducted using SAS software version 9.4 (SAS Institute Inc., Cary, NC, USA). Two-sided *P* values < 0.05 were recognized as statistically significant.

## 3 Results

### 3.1 Characteristics of participants

After a median follow-up period of 9.94 years, a total of 1,219 dementia cases were documented among 158,408 dementia-free participants. Participant characteristics based on the lowest and highest quartiles of non-free and free sugar intake are presented in Table 1**.** The findings revealed that individuals with elevated levels of non-free sugar intake were more likely to be women, older, experiencing higher levels of deprivation, having higher levels of education and income, non-smokers, and showing higher carbohydrate intake but lower fat, protein, and total energy intake. In contrast, participants with higher intake of free sugar tended to be younger men, experiencing higher levels of deprivation, possessing lower levels of education and higher levels of income, as well as smokers. Additionally, they showed higher total energy and carbohydrate intake, but lower fat and protein intake.

**Table 1:** Baseline characteristics according to the lowest and highest quartiles of non-free sugar and free sugars intake (*n* = 158,408).

**Characteristics**	**Total**	**Non-free sugar intake**		**Free sugar intake**	
		**Quartile 1**	**Quartile 4**	**Quartile 1**	**Quartile 4**
No. of participants	158,408	39,602	39,602	39,602	39,602
Intake of sugars of interest (% energy intake)		6.3 (1.7)	21.3 (5.0)	5.4 (1.9)	19.3 (4.7)
Sociodemographic factors					
Age at recruitment (years)	55.7 (8.0)	53.8 (8.1)	57.1 (7.6)	55.9 (7.8)	55.2 (8.2)
Female sex, n (%)	82,304 (52.0)	15,021 (37.9)	26,392 (66.6)	23,585 (59.6)	17,430 (44.0)
White ethnicity, n (%)	144,248 (91.1)	36,157 (91.3)	35,675 (90.1)	35,578 (89.8)	36,010 (90.9)
Townsend deprivation index	−1.6 (2.9)	−1.3 (3.0)	−1.7 (2.8)	−1.5 (2.9)	−1.4 (3.0)
College or university degree, n (%)	24,950 (15.8)	5493 (13.9)	6577 (16.6)	6702 (16.9)	5581 (14.1)
Income ≥ 52,000 EUR, n (%)	23,194 (14.6)	5396 (13.6)	6648 (16.8)	5730 (14.5)	6807 (17.2)
Lifestyle and dietary information					
BMI (kg/m^2^)	26.9 (4.6)	27.5 (4.6)	26.6 (4.6)	27.1 (4.8)	26.9 (4.5)
Current smoker, n (%)	12,488 (7.9)	5328 (13.5)	1811 (4.6)	2992 (7.6)	4185 (10.6)
Current alcohol drinker, n (%)	149,264 (94.2)	37,981 (95.9)	36,157 (91.3)	37,374 (94.4)	36,458 (92.1)
Physical activity (MET min/wk)	964.8 (1009.4)	926.1 (1023.8)	1035.4 (1027.0)	957.1 (991.5)	1001.5 (1067.9)
Total carbohydrate intake (% energy intake)	50.2 (8.5)	45.2 (8.7)	55.5 (7.5)	47.1 (9.5)	54.0 (7.7)
Total fat intake (% energy intake)	30.9 (6.6)	33.0 (7.0)	27.7 (6.1)	31.9 (7.3)	29.4 (6.3)
Total protein intake (% energy intake)	16.2 (3.6)	15.6 (3.8)	16.9 (3.9)	17.9 (4.2)	14.6 (3.2)
Total energy intake (kal/day)	8669.4 (2395.8)	9312.1 (2623.3)	7769.5 (2160.1)	8090.7 (2345.4)	8948.0 (2528.3)
Comorbidity, n (%)					
Cardiovascular disease	6776 (4.3)	1804 (4.6)	1718 (4.3)	1742 (4.4)	1869 (4.7)
Hypertension	81,326 (51.3)	20,503 (51.8)	20,389 (51.5)	20,460 (51.7)	20,377 (51.5)
Diabetes	8442 (5.3)	2093 (5.3)	2104 (5.3)	3262 (8.2)	1522 (3.8)
Laboratory indicators					
LDL‑C (mmol/L)	3.5 (0.8)	3.6 (0.8)	3.5 (0.9)	3.5 (0.9)	3.6 (0.8)
HDL‑C (mmol/L)	1.5 (0.4)	1.4 (0.4)	1.5 (0.4)	1.5 (0.4)	1.4 (0.4)
Triglycerides (mmol/L)	1.7 (1.0)	1.8 (1.1)	1.6 (0.9)	1.6 (1.0)	1.8 (1.0)
Family history of dementia	20,543 (13.0)	4565 (11.5)	5526 (14.0)	5143 (13.0)	4920 (12.4)
Incident dementia	1219 (0.8)	241 (0.6)	369 (0.9)	280 (0.7)	366 (0.9)

*Abbreviations:* BMI, body mass index; hs-CRP, high-sensitive C-reactive protein; HDL-C, high-density lipoprotein cholesterol; LDL-C, low-density lipoprotein cholesterol; MET, metabolic equivalent task; Q, quartile. Numbers are presented as means (standard deviation) unless otherwise specified as numbers (%).

### 3.2 Association between dietary sugar intake and incident dementia

In the multivariable cause-specific competing risk model, both non-free sugar and free sugar intake were positively associated with the risk of incident dementia (both *P* for trend < 0.01) (Table 2). Compared with participants in the lowest quartile of non-free and free sugar intake, those in the highest quartile had adjusted HRs (95% CIs) 1.26 (1.04, 1.52) and 1.43 (1.20, 1.70) for dementia, respectively. The adjusted HRs (95% CIs) for dementia per 5 % increase in energy from non-free and free sugar intake were 1.10 (1.04, 1.16) and 1.16 (1.10, 1.23), respectively. Moreover, using a restricted cubic spline curve, there was no evidence for a nonlinear association between non-free sugar intake and dementia risk (**Supplementary Fig 1A**). Conversely, a significant nonlinear relationship (*P* for nonlinearity = 0.038) was observed for free sugar intake and incident dementia risk (**Supplementary Fig 1B**). The risk of dementia remained relatively flat until free sugars constituted approximately 10 % of total energy, after which it started to increase rapidly. In the sensitivity analyses, the results were generally consistent with the main results when we (i) included BMI as a covariate (**Supplementary Table 3**); (ii) excluded dementia cases within the first 2 years of follow-up (**Supplementary Table 4**); (iii) restricted the analysis to participants who had completed ≥ 2 dietary assessments (**Supplementary Table 5**); (iv) used absolute intake of free and non-free sugar (g/day) as exposures (**Supplementary Table 6**); (v) used the ratio by dividing free sugar intake or non-free sugar intake by total carbohydrate intake (%) as exposures (**Supplementary Table 7**); and (vi) used Fine and Gray’s model instead of the cause-specific competing risk model (**Supplementary Table 9**).

**Table 2:** Associations of non-free and free sugars consumption with incident dementia (*n* = 158,408) a.

**Sugar intake**	**Cases (%)**	**Model 1**^c^	**Model 2**^d^	**Model 3**^e^
		**HR (95% CI)**	**HR (95% CI)**	**HR (95% CI)**
**Non-free sugar intakes (% energy intake)**				
**Quartile**				
<8.62 %	241 (0.61)	Reference	Reference	Reference
≥8.62 % and <12.11 %	279 (0.70)	1.15 (0.97, 1.36)	1.00 (0.84, 1.19)	1.02 (0.85, 1.21)
≥12.11 % and <16.32 %	330 (0.83)	1.35 (1.15, 1.60)	1.12 (0.94, 1.33)	1.14 (0.96, 1.36)
≥16.32 %	369 (0.93)	1.50 (1.28, 1.76)	1.22 (1.01, 1.47)	1.26 (1.04, 1.52)
***P*-trend**^b^		<0.0001	0.02	< 0.01
**Continuous (per 5% energy increase)**		1.12 (1.08, 1.17)	1.09 (1.03, 1.15)	1.10 (1.04, 1.16)
**Free sugar intakes (% energy intake)**				
**Quartile**				
<7.87 %	280 (0.71)	Reference	Reference	Reference
≥7.87 % and <11.08 %	279 (0.70)	1.00 (0.84. 1.18)	1.03 (0.87, 1.22)	1.06 (0.90, 1.26)
≥11.08 % and <14.78 %	294 (0.74)	1.05 (0.89, 1.24)	1.07 (0.90, 1.26)	1.11 (0.94, 1.32)
≥14.78 %	366 (0.92)	1.31 (1.12, 1.53)	1.36 (1.14, 1.62)	1.43 (1.20, 1.70)
***P*-trend**^b^		<0.001	<0.001	<0.0001
**Continuous (per 5 % energy increase)**		1.11 (1.06, 1.16)	1.15 (1.09, 1.21)	1.16 (1.10, 1.23)

*Abbreviations:* CIs, confidence intervals; HRs, hazard ratios.

a HRs and 95 % CIs were calculated by the cause-specific competing risk model.

b Test for trend based on variables containing the median value for each quartile.

c Model 1 was the crude model.

d Model 2 was adjusted for age at recruitment, sex, race, education level, average total household income before tax, Townsend Deprivation Index, physical activity, alcohol consumption status, smoking status, intakes of protein and fat (percentage of energy intake), and total energy intake.

e Model 3 was adjusted for Model 2 + history of hypertension, diabetes, and cardiovascular diseases, and family history of dementia (free sugar intake or non-free sugar intake were mutually adjusted for each other).

No significant heterogeneity across the majority of prespecified subgroups for the association of sugar intake with dementia was observed (*P* for interactions > 0.05 for most stratification factors) **(Supplementary Table 9)**. However, a significant interaction between free sugar intake and age (*P* for interaction = 0.04) was noted. The positive association of dementia risk with free sugar intake was slightly more pronounced among younger participants (≤ 60 years).

### 3.3 Association between dietary sugar intake, genetic susceptibility, and incident dementia

As shown in **Supplementary Fig 2**, there were no significant interaction effects between sugar intake and the TyG-PRS (both *P* for interaction > 0.05) on dementia risk. In the joint association analysis, the highest hazard of dementia was observed among participants with high TyG-PRS and in the highest quartile of sugar intake, with HRs (95% CIs) of 1.45 (1.13, 1.86) for non-free sugar and 1.92 (1.50, 2.44) for free sugar intake **(Supplementary Fig 3)**.

**Fig 2:**
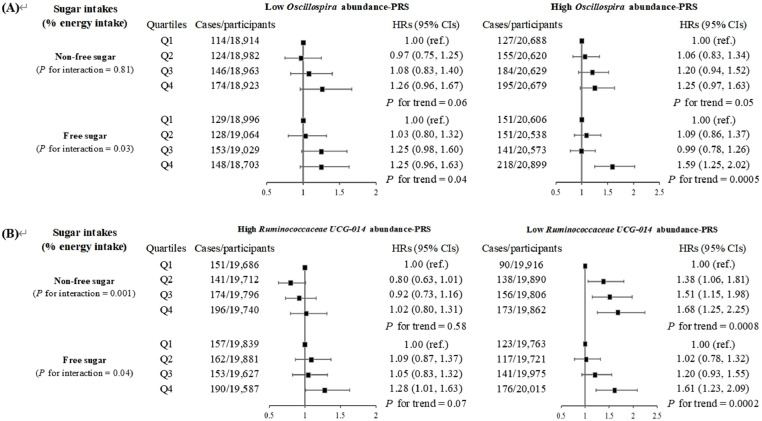
Associations of sugar intake with incident dementia according to PRS of gut microbial (*n* = 158,408). *Abbreviations:* CIs, confidence intervals; HRs, hazard ratios; PRS, polygenic risk score. HRs and 95 % CIs were calculated by the cause-specific competing risk model with adjustments for age at recruitment, sex, ethnicity, education level, average total household income before tax, Townsend Deprivation Index, physical activity, alcohol intake status, smoking status, intakes of protein and fat (percentage of energy intake), total energy intake, history of hypertension, diabetes, and cardiovascular diseases, and family history of dementia.

**Fig 3:**
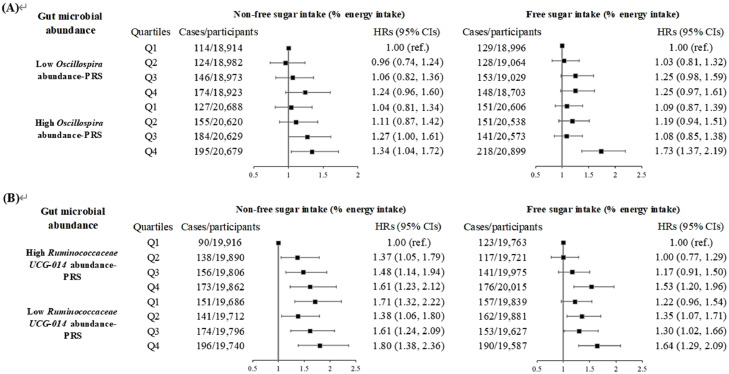
Joint associations of sugar intake and *Oscillospira-PRS* (A) or *Ruminococcaceae UCG-014* -PRS (B) with incident dementia (*n* = 158,408). *Abbreviations:* CIs, confidence intervals; HRs, hazard ratios; PRS, polygenic risk score. HRs and 95% CIs were calculated by the cause-specific competing risk model with adjustments for age at recruitment, sex, ethnicity, education level, average total household income before tax, Townsend Deprivation Index, physical activity, alcohol intake status, smoking status, intakes of protein and fat (percentage of energy intake), total energy intake, history of hypertension, diabetes, and cardiovascular diseases, and family history of dementia.

Then, we investigated the modifying effect of gut microbiota on the association of dementia risk with sugar intake. As outlined in **Supplementary Table 10**, increased risk of dementia was positively associated with *Oscillospira* PRS, but negatively linked to PRS of *Ruminococcaceae UCG-014* with adjustment of FDR. Moreover, as listed in Fig 2, a significant interaction effect was only observed between free sugar intake and PRS of *Oscillospira* (*P* for interaction = 0.03); in other words, individuals with high PRS of *Oscillospira* were more likely to develop dementia with HRs (95% CIs) of 1.59 (1.25, 2.02). On the contrary, there were significant interaction effects of *Ruminococcaceae UCG-014*-PRS on the associations between non-free sugar and free sugar intake and dementia risk (both *P* for interaction < 0.05). Those with high PRS of *Ruminococcaceae UCG-014* showed an increased risk of dementia (HRs, 95% CIs: 1.68, 1.25–2.25 for non-free sugar intake; 1.61, 1.23–2.09 for free sugar intake). In the joint association analysis, the highest hazard of dementia was observed among participants with high PRS of *Oscillospira* and in the highest quartile of sugar intake, with HRs (95% CIs) of 1.34 (1.04, 1.72) for non-free sugar and 1.73 (1.37, 2.19) for free sugar intake (Fig 3**A**). However, for *Ruminococcaceae UCG-014*, the highest hazard of dementia was observed among participants with low PRS of *Ruminococcaceae UCG-014* and in the highest quartile of sugar intake, with HRs (95% CIs) of 1.80 (1.38, 2.36) for non-free sugar and 1.64 (1.29, 2.09) for free sugar intake (Fig 3**B**). After additionally adjusting for the total relative abundance of gut microbial taxa, the results were still consistent with the main results (data not shown).

The positive association between disease susceptibility and dementia risk was confirmed in **Supplementary Table 11.** Compared with participants with low dementia-PRS (< median value), those with high dementia-PRS (≥ median value) had an adjusted HR (95% CI) of 2.30 (2.03, 2.60) for dementia. The associations of sugar intake with incident dementia according to dementia-PRS are presented in **Supplementary Fig 4**. There was a significant interaction effect of dementia-PRS on the associations between non-free sugar intake and dementia risk (*P* for interaction = 0.04). The positive association between non-free sugar intake and dementia risk was observed only in the high dementia-PRS subgroup (*P* for trend < 0.01), but not in the low dementia-PRS subgroup (*P* for trend = 0.13). However, intake of free sugar was positively associated with risk of dementia in both high and low dementia-PRS subgroups, with no significant interaction of dementia-PRS detected (*P* for interaction = 0.37). In the joint association analysis, the highest hazard of dementia was observed among participants with high dementia-PRS and in the highest quartile of sugar intake, with HRs (95% CIs) of 2.59 (1.99, 2.38) for non-free sugar and 3.09 (2.38, 4.00) for free sugar intake **(Supplementary Fig 5)**.

## 4 Discussion

In the current cohort study of 158,408 participants from the UK Biobank, we found that there is a synergistic effect of dietary sugar intake and genetic susceptibility on incident dementia. This comprehensive analysis incorporated three dimensions of genetic susceptibility: sugar metabolism, gut microbiota (represented by *Ruminococcaceae UCG-014* and *Oscillospira* PRS), and disease susceptibility. Intriguingly, we provided the first evidence that individuals with high TyG-PRS, as well as with low PRS of *Ruminococcaceae UCG-014* or high PRS of *Oscillospira*, demonstrate substantially heightened dementia risk when consuming high-sugar diets.

Sugar intake, particularly free sugar intake, has been identified as a risk factor for the onset and progression of dementia. Evidence from the United States Women’s Health Initiative-Dietary Modification Trial and the Rush Memory and Aging Project both reported a significant association between excessive total sugar intake and dementia [34,35]. Specifically, an increase of 10 *g* per day in total sugar intake was associated with a 1.3 %–1.4 % elevated risk of AD [35]. Studies from the United States community-based Framingham Heart Study have further supported the closely association between dementia risk and higher intake of sugar in beverages (HR: 2.80; 95% CI: 2.24, 3.50) [36] and artificially sweetened soft drinks (HR: 2.20; 95% CI: 1.09, 4.45) [8]. Another study based on 186,622 UK Biobank participants revealed a “J-shaped” association between increased sugar intake and dementia risk, with the HR-nadir observed at 9 % total energy [10]. However, to the best of our knowledge, only one study from the UK reported a linear-shaped association between non-free sugar intake and dementia risk [10], consistent with our findings. Subgroup analyses confirmed these associations remained robust after adjustment for demographic and lifestyle covariates, though the free sugar-dementia link exhibited particular prominence in younger adults (≤ 60 years), likely reflecting their greater consumption of processed sweets and beverages. Contrary to prevailing assumptions about non-free sugars' protective effects against cardiovascular disease [11,37], our data underscore their underappreciated neurocognitive risks.

The pathophysiological mechanisms underlying sugar-induced neurodegeneration remain incompletely characterized but appear multifaceted. Free sugars include all monosaccharides and disaccharides added to foods by the manufacturer, cook, or consumer, plus sugars naturally present in honey, syrups, and unsweetened fruit juices. Non-free sugars are made up of all sugars excluded from the definition of free sugars, mostly naturally occurring in fruit, vegetables, and dairy products, which are enclosed within plant cell walls or the food matrix, requiring chewing or digestion in the intestines to be gradually released [38]. Because free sugars are not encapsulated by the cell walls, they can quickly enter the bloodstream and exert effects. On the one hand, studies have demonstrated that the brain, as the most energy-hungry organ, relies heavily on a quarter-pound daily supply of glucose to produce the ATP necessary for its function [39]. Animal models of dementia have revealed that excessive intake of fructose, present in refined sugars such as sucrose and high-fructose corn syrup, may contribute to dementia by increasing central neuronal insulin resistance and amyloid deposition, a factor linked to AD [40,41]. On the other hand, dementia, including AD, is essentially an age-related disease where excessive sugar intake may accelerate cellular aging through inflammatory responses and oxidative stresses [42]. Despite the absence of a chemical difference between non-free sugars and free sugars, further studies are needed to elucidate the underlying mechanisms.

In addition, our findings suggested that genetic susceptibility is also an important factor influencing the effect of sugar intake on dementia. In the present study, we first explored it from three related aspects: sugar metabolism, gut microbiota, and disease susceptibility. The TyG Index is an economical and effective diagnostic tool for several medical conditions. It not only reflects underlying insulin resistance but is also considered a key factor in several metabolic disorders (including dementia) [43]. Published meta-analyses have demonstrated that the TyG Index is associated with an increased risk of dementia [43,44]. Consistently, we found that individuals who consumed more sugar in their diet and had a high PRS of TyG are more likely to develop dementia.

Then, we explored the interaction effect of dementia-PRS on such associations. There was no significant interaction between dementia-PRS and free sugar, but an interacting effect of dementia-PRS was observed in the association between non-free sugar intake and dementia risk. Specifically, individuals with high dementia-PRS showed an increased dementia risk with non-free sugar intake. Moreover, the joint effect analysis revealed that the highest hazard of dementia was observed in individuals with high AD-PRS and the highest intake of free sugar or non-free sugar intake. These results strongly suggest the importance of controlling the quantity of free sugar and non-free sugar intake, especially for individuals with a high hereditary risk of dementia.

More importantly, mounting evidence underscores the pivotal role of dietary patterns in modulating gut microbial ecology, with profound implications for disease pathogenesis [45]. At the gut level, sugar and elevated blood sugar strongly influence the gut microbiota and gut barrier by increasing permeability and susceptibility to infection [46]. Evidence from both human and animal studies has revealed that the intake of non-caloric artificial sweeteners (a type of free sugar) enhances the risk of glucose intolerance by influencing the composition and function of the microbiota [47]. Another animal study reported that the intake of sugary drinks during adolescence leads to alterations in the gut microbiota, including an elevated abundance of two species in the genus *Parabacteroides* (*P. distasonis* and *P. johnsonii*), which are inversely associated with hippocampal function [48]. However, according to 119 kinds of gut microbial-PRS, we found that different gut microbial show inconsistent effects on dementia. The *Oscillospira* emerged as a significant risk factor, whereas *Ruminococcaceae UCG-014* demonstrated protective effects. Both microbial PRS scores exhibited interactive effects with dietary sugar consumption on dementia risk. The paradoxical nature of *Oscillospira* warrants particular attention - while recognized as a butyrate producer and potential next-generation probiotic candidate [49], this bacterium has been implicated in the pathogenesis of type 2 diabetes [50], Parkinson’s disease [51], and dementia [52]. Supporting our findings, animal models indicate that *Oscillospira* enrichment predicts activation of the farnesoid X receptor pathway, a known dementia risk factor [52]. In contrast, although *Ruminococcaceae UCG-014*′s anti-obesity properties are well-documented [53], its neuroprotective potential remains unexplored. We hypothesize that this bacterium may confer cognitive benefits through enhanced dietary fiber fermentation, yielding butyrate and other neuroprotective short-chain fatty acids that mitigate *β*-amyloid accumulation and neuroinflammation [[54], [55], [56]]. Interestingly, it should be noted that *Ruminococcaceae UCG-014* and *Oscillospira* belong to the same genus *Firmicute* - *Clostridia* – *Ruminococcaceae*, which makes the two exhibit some similar physiological functions. Although previous evidence has shown that high sugar intake could reduce the abundance of *Ruminococcaceae UCG-014* and *Oscillospira* in the intestine, thereby inhibiting butyric acid production and affecting cognitive function [57,58]. However, the differential responses of *Ruminococcaceae UCG-014* and *Oscillospira* on the association between dietary sugar intake and dementia demonstrated that beyond dietary modulation of microbial abundance, the host's genetically determined microbial functional capacity represents an independent and critical determinant of neurological outcomes. This finding fundamentally advances our understanding of diet-microbiome-brain interactions by emphasizing the need to consider both environmental exposures and host genetic factors in predictive models of neurodegeneration.

To the best of our knowledge, this study represents the first exploration of the association between non-free sugar intake, genetic factors, and incident dementia within a large and representative cohort. Comprehensive subgroup and sensitivity analyses further confirmed the robustness of the findings. Besides, based on previous studies, limiting the intake of free sugars is good for health [59], while the harmful effects of non-free sugar intake on health are not clear. Our findings, therefore, have important public health value, offering new insights to prompt a re-evaluation of the potential role of non-free sugar intake. Last but not least, our findings indicate that the influence of genetic susceptibility on the association between dietary sugar intake and dementia may help identify the high-risk population and investigate the potential therapeutic target for mitigating the onset of dementia.

Several potential limitations should be acknowledged. First, reliance on self-reported data regarding dietary patterns may introduce information bias. To address this, we mitigated the issue by utilizing the average dietary assessment of each individual over a 24-hour period and further excluding implausible intake. Second, due to the inherent limitations of an observational study design, residual confounders could not be eliminated, although we did adjust for several potential confounders. Third, this study primarily focused on the impact of the types of sugar intake on dementia risk, and it remains unclear whether there are distinctions between non-free and free sugars in specific forms, such as solid and liquid. Therefore, further research is still warranted. Fourth, the UK Biobank consists largely of relatively healthy, affluent individuals who are predominantly white. As a result, our findings may not be generalizable to the entire population. Fifth, given the fact that feces are difficult to preserve and collect, the UK Biobank does not include fecal samples in the research design; the PRS we calculated could not represent the real profile of gut microbiota. Finally, our findings provide clues for slowing the development of dementia by improving the gut microbiota through diet, but given that the effects of various foods on the gut microbiota are not fully understood, we cannot make exact food recommendations.

## 5 Conclusions

In summary, both non-free sugar and free sugar intake are risk factors for the incidence of dementia, suggesting that intake of non-free sugars should also be limited. What’s more, genetic susceptibility may play a pivotal role in the association between dementia risk and dietary sugar intake.

**Abbreviations**
AD: Alzheimer’s disease
BMI: Body mass index
CI: confidence interval
CVD: cardiovascular disease
HR: hazard ratio
ICD: International Classification of Diseases
MET: metabolic equivalent
PRS: polygenic risk score
TDI: Townsend Deprivation Index

## Ethics approval and consent to participant

The study protocol of the UK Biobank has been approved by the North West Multi-Center Research’s Ethics Committee (REC reference: NW/0382) and conducted in accordance with the principles of the Declaration of Helsinki. All individuals provided written informed consent prior to recruitment.

## Consent for publication

Not applicable

## Availability of data and materials

Data are available in a public, open access repository. This research has been conducted using the UK Biobank Resource under Application Number 63,454. The UK Biobank data are available on application to the UK Biobank (www.ukbiobank.ac.uk/).

## Funding

This work was supported by the Young Elite Scientists Sponsorship Program by China Association for Science and Technology [grant number 2020QNRC001 to Yang Xia and Yu An, 2024-2026QNRC001 to Xiaopeng Zhang], the LiaoNing Revitalization Talents Program [grant number XLYC2203168 to Yang Xia], the Beijing Hospitals Authority Youth Programme [grant number QML20230301 to Yu An], and Tianjin Natural Science Foundation [grant number 23JCQNJC01830 to Limin Cao). The funders had no role in the conduct of the study; collection, management, analysis, or interpretation of the data; preparation, review, or approval of the manuscript; or decision to submit the manuscript for publication.

## Supplementary Material

**Supplementary Table 1.** Characteristics of the SNPs associated with genus-level TyG.

**Supplementary Table 2.** Characteristics of the SNPs associated with genus-level gut microbes.

**Supplementary Fig 1.** Restricted cubic splines of the associations of dementia risk with non-free sugar (A) and sugar (B) intake.

**Supplementary Table 3.** Sensitivity analysis of the associations between non-free and free sugar intake and incident dementia with including BMI as a covariate (*n* = 158,408) ^a^

**Supplementary Table 4.** Associations of non-free and free sugar intake with incident dementia by excluding participants with less than two years of follow-up (*n* = 157,541) ^a^.

**Supplementary Table 5.** Associations of non-free and free sugar intake with incident dementia by excluding participants with less than two dietary assessments (*n* = 97,821) ^a^.

**Supplementary Table 6.** Associations of absolute non-free and free sugar intake with incident dementia (*n* = 158,408) ^a^.

**Supplementary Table 7.** Associations of non-free and free sugar to carbohydrates intake ratio with incident dementia (*n* = 158,408) ^a^.

**Supplementary Table 8.** Associations of non-free and free sugar intake with incident dementia using the sub-distribution competing risk model (*n* = 158,408) ^a^.

**Supplementary Table 9.** Subgroup analyses for the associations of non-free sugar and free sugars intakes ( % energy intake) with incident dementia (*n* = 158,408) ^a^.

**Supplementary Fig 2.** Associations of sugar intake with incident dementia according to the PRS of TyG (*n* = 158,408).

**Supplementary Fig 3.** Joint associations of sugar intake and the PRS of TyG with incident dementia (*n* = 158,408).

**Supplementary Table 10.** Associations between genetic risk of gut microbial and incident dementia (*n* = 158,408)

**Supplementary Table 11.** Associations between genetic risk of dementia and incident dementia (*n* = 158,408).

**Supplementary Fig 4.** Associations of sugar intake with incident dementia according to genetic risk (*n* = 158,408).

**Supplementary Fig 5.** Joint associations of sugar intake and genetic risk with incident dementia (*n* = 158,408).

## CRediT authorship contribution statement

**Yu An:** Project administration, Investigation, Funding acquisition. **Limin Cao:** Writing – original draft, Software, Funding acquisition, Conceptualization. **Gang Zheng:** Resources, Methodology, Formal analysis. **Yashu Liu:** Visualization, Methodology, Investigation. **Honghao Yang:** Methodology, Investigation. **Liangkai Chen:** Resources, Data curation. **Yuhong Zhao:** Resources, Conceptualization. **Xiaopeng Zhang:** Writing – review & editing, Funding acquisition. **Yang Xia:** Writing – review & editing, Validation, Funding acquisition, Data curation, Conceptualization.

## Declaration of competing interest

The authors declare that they have no known competing financial interests or personal relationships that could have appeared to influence the work reported in this paper.

## Appendix Supplementary materials

Image, application 1
