# Supplementary material for: Dietary sugar intake, genetic susceptibility, and risk of dementia: A prospective cohort study
Source: J Prev Alzheimers Dis. 2025 Jul 26;12(9):100312. doi: 10.1016/j.tjpad.2025.100312 (PMC12501323; doi:10.1016/j.tjpad.2025.100312)
Supplement: Supplementary file 1 [file mmc1.docx]

**Dietary sugar intake, gut microbiota, and risk of dementia: a prospective cohort study**

**Running title:** sugar, gut microbiota and dementia

Yu An, Ph.D. ^1#^, Limin Cao, Ph.D. ^2#^, Gang Zheng, Ph.D. ^3, 4#^, Yashu Liu, Ph.D. ^5^, Honghao Yang, Ph.D. ^3, 4^, Liangkai Chen, Ph.D. ^6^, Yuhong Zhao, Ph.D. ^3, 4^, Xiaopeng Zhang, Ph.D. ^7*^, Yang Xia, Ph.D. ^3, 4*^

1. Medical Research Center, Beijing Institute of Respiratory Medicine and Beijing Chao-Yang Hospital, Capital Medical University, 100020, Beijing, China.

2. The Third Central Hospital of Tianjin, No. 83, Jin Tang Road, Tianjin, 300070, China.

3. Department of Clinical Epidemiology, Shengjing Hospital of China Medical University, Shenyang, No. 36, San Hao Street, Shenyang, Liaoning, 110004, China.

4. Liaoning Key Laboratory of Precision Medical Research on Major Chronic Disease, Shenyang, No. 36, San Hao Street, Shenyang, Liaoning, 110004, China.

5. Department of General Surgery, Shengjing Hospital of China Medical University, Shenyang, No. 36, San Hao Street, Shenyang, Liaoning, 110004, China.

6. Department of Nutrition and Food Hygiene, Hubei Key Laboratory of Food Nutrition and Safety, School of Public Health, Tongji Medical College, Huazhong University of Science and Technology, No. 13, Hang Kong Road, 430030, Wuhan, China.

7. Key Laboratory of Carcinogenesis and Translational Research (Ministry of Education/Beijing), Gastrointestinal Cancer Center, Peking University Cancer Hospital and Institute, Beijing 100142, China.

^#^ These three authors contributed equally to this work.

**^*^ Address for correspondence to: Xiaopeng Zhang or Yang Xia**

**Corresponding author information:**

1. Yang Xia, Ph.D., Department of Clinical Epidemiology, Shengjing Hospital of China Medical University, Shenyang, No. 36, San Hao Street, Shenyang, Liaoning, 110004, China. Email: xytmu507@126.com

2. Xiaopeng Zhang, PhD., MD., Key Laboratory of Carcinogenesis and Translational Research (Ministry of Education/Beijing), Gastrointestinal Cancer Center, Peking University Cancer Hospital and Institute, Beijing 100142, China. Tel: +86-010-88196606. E-mail: [xiaopeng.zhang@bjmu.edu.cn](mailto:xiaopeng.zhang@bjmu.edu.cn)

Number of characters in the title and running head: 86 and 34 characters.

Number of words in the Abstract, Introduction, Discussion and the body of the manuscript: 240, 443, 1340, and 4255 words.

Number of figures, color figures, and tables: 3, 0, and 2.

**Supplementary Table 1. Characteristics of the SNPs associated with genus-level TyG.**

| **SNP** | **chr** | **A1** | **A2** | **beta** | **se** | **P** |
| --- | --- | --- | --- | --- | --- | --- |
| rs114165349 | 1 | C | G | 0.0445298 | 0.00465259 | 1.07E-21 |
| rs72904790 | 1 | C | T | -0.0137147 | 0.00240632 | 1.20E-08 |
| rs213498 | 1 | A | T | -0.00802754 | 0.00146386 | 4.17E-08 |
| rs10889332 | 1 | T | C | -0.0388967 | 0.00143046 | 1.34E-162 |
| rs72669514 | 1 | T | C | 0.018609 | 0.00320686 | 6.53E-09 |
| rs17656269 | 1 | T | C | 0.00874937 | 0.00147002 | 2.65E-09 |
| rs16836630 | 1 | C | G | -0.0173377 | 0.00252122 | 6.14E-12 |
| rs1760801 | 1 | A | G | -0.00895698 | 0.0015125 | 3.19E-09 |
| rs340836 | 1 | C | T | -0.00869811 | 0.00139646 | 4.71E-10 |
| rs76172548 | 1 | C | A | 0.0228139 | 0.00383242 | 2.64E-09 |
| rs3120619 | 1 | A | G | 0.0118809 | 0.0018033 | 4.45E-11 |
| rs11118610 | 1 | C | A | -0.00902819 | 0.00138913 | 8.09E-11 |
| rs4846922 | 1 | T | C | 0.0221545 | 0.00146286 | 8.63E-52 |
| rs907866 | 2 | A | G | -0.00927654 | 0.00138756 | 2.31E-11 |
| rs111585158 | 2 | T | C | 0.0121133 | 0.00211397 | 1.00E-08 |
| rs144470864 | 2 | C | A | 0.0172612 | 0.00307793 | 2.05E-08 |
| rs76384951 | 2 | C | A | -0.0295509 | 0.0025237 | 1.16E-31 |
| rs533617 | 2 | C | T | -0.0464535 | 0.00346743 | 6.48E-41 |
| rs35750610 | 2 | C | T | 0.0185128 | 0.00234709 | 3.09E-15 |
| rs34921778 | 2 | G | A | 0.00842606 | 0.00144997 | 6.21E-09 |
| rs12617848 | 2 | T | C | 0.0142794 | 0.00200585 | 1.09E-12 |
| rs80216311 | 2 | T | C | -0.0141686 | 0.00238957 | 3.05E-09 |
| rs61737373 | 2 | A | G | -0.028607 | 0.00292878 | 1.57E-22 |
| rs6547692 | 2 | G | A | 0.0375073 | 0.00138448 | 2.05E-161 |
| rs10206462 | 2 | C | T | -0.00893744 | 0.00142769 | 3.85E-10 |
| rs6760053 | 2 | G | C | -0.00778921 | 0.00137713 | 1.55E-08 |
| rs6710938 | 2 | C | A | -0.00898973 | 0.00161797 | 2.76E-08 |
| rs79953491 | 2 | G | A | -0.0237048 | 0.00210804 | 2.49E-29 |
| rs115128825 | 2 | A | C | 0.026901 | 0.00487631 | 3.46E-08 |
| rs484066 | 2 | A | T | -0.0159198 | 0.00141892 | 3.31E-29 |
| rs17694506 | 2 | C | T | 0.00900471 | 0.00141859 | 2.19E-10 |
| rs2943645 | 2 | C | T | -0.0209209 | 0.0014316 | 2.40E-48 |
| rs6437249 | 2 | T | C | 0.00838469 | 0.00149284 | 1.95E-08 |
| rs147764624 | 3 | C | G | -0.03019 | 0.00550582 | 4.18E-08 |
| rs390802 | 3 | A | G | -0.0153354 | 0.00176462 | 3.63E-18 |
| rs62271373 | 3 | A | T | 0.0253586 | 0.00303878 | 7.15E-17 |
| rs13074711 | 3 | C | T | 0.0120107 | 0.00218488 | 3.86E-08 |
| rs13108218 | 4 | A | G | 0.0156395 | 0.00143815 | 1.54E-27 |
| rs71603401 | 4 | G | A | 0.0125089 | 0.00205483 | 1.15E-09 |
| rs6448429 | 4 | T | C | 0.0126753 | 0.00187808 | 1.49E-11 |
| rs1471251 | 4 | T | A | 0.0164471 | 0.00140761 | 1.56E-31 |
| rs4134363 | 4 | A | G | -0.00950005 | 0.00170166 | 2.37E-08 |
| rs3822076 | 4 | A | T | 0.00867089 | 0.0013818 | 3.50E-10 |
| rs2035816 | 4 | G | A | -0.0159447 | 0.00249077 | 1.54E-10 |
| rs78025076 | 4 | T | C | 0.0271124 | 0.00482777 | 1.96E-08 |
| rs390556 | 5 | C | T | -0.0132704 | 0.00220378 | 1.73E-09 |
| rs72754154 | 5 | A | G | -0.0213999 | 0.00314107 | 9.58E-12 |
| rs3936511 | 5 | G | A | 0.0216126 | 0.00174659 | 3.69E-35 |
| rs151913 | 5 | A | G | 0.00786984 | 0.00141384 | 2.60E-08 |
| rs7703744 | 5 | G | C | -0.0109345 | 0.00155172 | 1.84E-12 |
| rs72801474 | 5 | A | G | -0.0146422 | 0.00235286 | 4.88E-10 |
| rs12173130 | 5 | C | T | 0.00971075 | 0.00176945 | 4.07E-08 |
| rs11134475 | 5 | A | G | -0.0169417 | 0.00142319 | 1.15E-32 |
| rs2963476 | 5 | G | A | 0.0133691 | 0.00169886 | 3.58E-15 |
| rs6923241 | 6 | T | C | -0.0109234 | 0.00154721 | 1.67E-12 |
| rs2745400 | 6 | A | G | 0.00826032 | 0.00137239 | 1.76E-09 |
| rs2894211 | 6 | A | C | 0.0174652 | 0.00218851 | 1.46E-15 |
| rs7758790 | 6 | C | T | 0.0142803 | 0.00164795 | 4.52E-18 |
| rs55697600 | 6 | G | A | 0.0351072 | 0.00360432 | 2.05E-22 |
| rs185139895 | 6 | A | G | 0.0207564 | 0.00338431 | 8.63E-10 |
| rs3025053 | 6 | A | G | -0.0134581 | 0.00212481 | 2.40E-10 |
| rs4715317 | 6 | T | G | 0.00974329 | 0.00143947 | 1.30E-11 |
| rs1967685 | 6 | C | G | -0.0142769 | 0.0013714 | 2.25E-25 |
| rs632057 | 6 | T | G | 0.0153665 | 0.00142077 | 2.94E-27 |
| rs12208357 | 6 | T | C | 0.0219558 | 0.00272455 | 7.75E-16 |
| rs77009508 | 6 | G | A | 0.0224178 | 0.00259593 | 5.86E-18 |
| rs55730499 | 6 | T | C | -0.0182481 | 0.0025524 | 8.74E-13 |
| rs186696265 | 6 | T | C | -0.0472632 | 0.00592501 | 1.51E-15 |
| rs4709746 | 6 | T | C | -0.0112466 | 0.00203182 | 3.11E-08 |
| rs852424 | 7 | T | C | 0.00852818 | 0.0014628 | 5.55E-09 |
| rs38205 | 7 | A | C | 0.00791466 | 0.00143983 | 3.87E-08 |
| rs2106727 | 7 | A | G | -0.0108194 | 0.00142823 | 3.59E-14 |
| rs4722551 | 7 | C | T | -0.0185803 | 0.00188091 | 5.21E-23 |
| rs1534696 | 7 | C | A | 0.0107368 | 0.00137638 | 6.18E-15 |
| rs2971676 | 7 | A | G | 0.0133494 | 0.00239848 | 2.61E-08 |
| rs878521 | 7 | A | G | 0.0217619 | 0.00158662 | 8.43E-43 |
| rs62459110 | 7 | C | G | -0.0213924 | 0.00364332 | 4.32E-09 |
| rs799157 | 7 | T | C | 0.0407906 | 0.00340184 | 4.05E-33 |
| rs17145750 | 7 | T | C | -0.0560614 | 0.00185642 | 5.28E-200 |
| rs10260148 | 7 | T | C | 0.0149589 | 0.00154174 | 2.96E-22 |
| rs73198299 | 8 | C | T | 0.0122709 | 0.00222876 | 3.68E-08 |
| rs7821812 | 8 | C | G | 0.0163357 | 0.00169741 | 6.39E-22 |
| rs904009 | 8 | C | A | 0.0159306 | 0.00162585 | 1.16E-22 |
| rs4921914 | 8 | C | T | 0.0194386 | 0.00165914 | 1.07E-31 |
| rs2975424 | 8 | C | T | 0.0106046 | 0.0017554 | 1.53E-09 |
| rs1388941 | 8 | A | G | 0.0143536 | 0.00145941 | 8.01E-23 |
| rs268 | 8 | G | A | 0.10865 | 0.00515243 | 1.25E-98 |
| rs117026536 | 8 | T | G | -0.0951759 | 0.00226332 | 0 |
| rs57295072 | 8 | C | G | -0.0304863 | 0.00469748 | 8.60E-11 |
| rs17091881 | 8 | C | T | 0.0785949 | 0.00427426 | 1.82E-75 |
| rs74444445 | 8 | C | T | 0.034928 | 0.00488632 | 8.82E-13 |
| rs117805502 | 8 | T | C | -0.0321654 | 0.00439412 | 2.48E-13 |
| rs28550053 | 8 | G | A | -0.0177064 | 0.00182104 | 2.42E-22 |
| rs75662196 | 8 | C | G | -0.0279294 | 0.00434519 | 1.30E-10 |
| rs17092008 | 8 | T | C | 0.0208253 | 0.00285324 | 2.91E-13 |
| rs11781356 | 8 | A | T | 0.00993171 | 0.00176553 | 1.85E-08 |
| rs2081687 | 8 | T | C | 0.011677 | 0.00145382 | 9.63E-16 |
| rs71525127 | 8 | G | C | 0.019603 | 0.00254745 | 1.42E-14 |
| rs11558471 | 8 | G | A | -0.011475 | 0.00147145 | 6.29E-15 |
| rs17321515 | 8 | G | A | -0.0439 | 0.00137141 | 2.01E-224 |
| rs62521590 | 8 | G | T | 0.0146537 | 0.00155763 | 5.11E-21 |
| rs10811661 | 9 | C | T | -0.00987338 | 0.0018054 | 4.53E-08 |
| rs13289566 | 9 | T | C | -0.0118284 | 0.00166888 | 1.37E-12 |
| rs2244278 | 9 | A | C | -0.0133902 | 0.00211876 | 2.62E-10 |
| rs3750571 | 10 | A | C | -0.0124087 | 0.00189812 | 6.27E-11 |
| rs11006681 | 10 | A | G | -0.0110061 | 0.00184368 | 2.38E-09 |
| rs142164605 | 10 | A | T | -0.0177614 | 0.00278129 | 1.71E-10 |
| rs10786069 | 10 | C | T | 0.0130977 | 0.00137832 | 2.06E-21 |
| rs113344423 | 10 | A | G | 0.0212993 | 0.00301795 | 1.70E-12 |
| rs2792736 | 10 | T | A | -0.0100505 | 0.00153811 | 6.40E-11 |
| rs10832027 | 11 | G | A | -0.0122569 | 0.00148245 | 1.37E-16 |
| rs3808976 | 11 | G | A | 0.00981886 | 0.00170108 | 7.84E-09 |
| rs99780 | 11 | T | C | 0.020203 | 0.00143553 | 5.73E-45 |
| rs35169799 | 11 | T | C | 0.0247241 | 0.00283283 | 2.61E-18 |
| rs678614 | 11 | A | C | 0.00935072 | 0.00153217 | 1.04E-09 |
| rs2302883 | 11 | C | T | 0.00886478 | 0.00162296 | 4.71E-08 |
| rs187217942 | 11 | A | G | 0.031159 | 0.00540272 | 8.06E-09 |
| rs17119701 | 11 | G | A | 0.0370675 | 0.00375036 | 4.94E-23 |
| rs61362984 | 11 | G | A | -0.0139461 | 0.00142207 | 1.06E-22 |
| rs61904855 | 11 | A | C | 0.0233784 | 0.00409645 | 1.15E-08 |
| rs11216122 | 11 | T | G | -0.0181908 | 0.00310342 | 4.59E-09 |
| rs7930786 | 11 | C | G | 0.124688 | 0.00278666 | 0 |
| rs56225305 | 11 | A | G | 0.108415 | 0.00278601 | 0 |
| rs2075294 | 11 | T | G | 0.0388118 | 0.00579905 | 2.19E-11 |
| rs75919952 | 11 | T | C | -0.046805 | 0.0031699 | 2.56E-49 |
| rs11600380 | 11 | C | T | -0.03673 | 0.00254055 | 2.34E-47 |
| rs5110 | 11 | A | C | -0.0185398 | 0.00248476 | 8.59E-14 |
| rs12721078 | 11 | A | C | -0.0322797 | 0.0039376 | 2.46E-16 |
| rs71480323 | 11 | A | G | -0.0195603 | 0.00211444 | 2.24E-20 |
| rs11216236 | 11 | T | C | 0.0240392 | 0.00342403 | 2.21E-12 |
| rs187929675 | 11 | T | C | -0.0767868 | 0.00606752 | 1.07E-36 |
| rs11045171 | 12 | G | A | -0.0116623 | 0.00173721 | 1.91E-11 |
| rs67981690 | 12 | G | A | 0.0148562 | 0.00207488 | 8.09E-13 |
| rs10783828 | 12 | A | G | 0.00903509 | 0.00147493 | 9.04E-10 |
| rs7296326 | 12 | C | T | -0.0118561 | 0.00217251 | 4.84E-08 |
| rs1585705 | 12 | C | A | 0.00876614 | 0.001496 | 4.64E-09 |
| rs10861679 | 12 | C | T | 0.00935439 | 0.00150663 | 5.35E-10 |
| rs1882491 | 12 | C | T | -0.0134975 | 0.00148108 | 8.04E-20 |
| rs1716407 | 12 | G | A | -0.0150613 | 0.00139861 | 4.90E-27 |
| rs7140110 | 13 | C | T | 0.0143695 | 0.00150803 | 1.61E-21 |
| rs112740904 | 13 | G | T | -0.0149023 | 0.00195835 | 2.76E-14 |
| rs12885801 | 14 | A | C | 0.00908515 | 0.00162151 | 2.11E-08 |
| rs34820917 | 14 | A | G | -0.0157999 | 0.00285011 | 2.97E-08 |
| rs35477346 | 15 | C | T | 0.00929684 | 0.00149721 | 5.32E-10 |
| rs139974673 | 15 | C | T | 0.0717689 | 0.00443008 | 5.34E-59 |
| rs72739147 | 15 | T | A | -0.0121232 | 0.00206079 | 4.04E-09 |
| rs1532085 | 15 | A | G | 0.0180035 | 0.0014104 | 2.64E-37 |
| rs261334 | 15 | G | C | 0.0261448 | 0.00167767 | 9.88E-55 |
| rs11636087 | 15 | C | T | 0.0116533 | 0.00154515 | 4.65E-14 |
| rs8028620 | 15 | C | T | -0.00897866 | 0.00137468 | 6.53E-11 |
| rs7175132 | 15 | G | A | -0.00811532 | 0.00141367 | 9.44E-09 |
| rs8025505 | 15 | T | C | 0.00964703 | 0.00158419 | 1.13E-09 |
| rs9935836 | 16 | C | A | 0.00988172 | 0.00177279 | 2.49E-08 |
| rs11075253 | 16 | A | C | -0.0141141 | 0.00150196 | 5.65E-21 |
| rs12446515 | 16 | T | C | -0.0187602 | 0.00147498 | 4.76E-37 |
| rs5880 | 16 | C | G | 0.0221143 | 0.00298074 | 1.18E-13 |
| rs12934528 | 16 | C | T | 0.0135148 | 0.00195818 | 5.15E-12 |
| rs2925979 | 16 | T | C | 0.0154375 | 0.00149811 | 6.79E-25 |
| rs11651957 | 17 | A | G | 0.0186479 | 0.00293615 | 2.14E-10 |
| rs12937081 | 17 | G | A | 0.0108836 | 0.00189096 | 8.64E-09 |
| rs72836561 | 17 | T | C | 0.0682178 | 0.00389919 | 1.69E-68 |
| rs231539 | 17 | T | C | 0.0130645 | 0.00188077 | 3.76E-12 |
| rs11657238 | 17 | A | G | -0.00784868 | 0.00138378 | 1.41E-08 |
| rs1801689 | 17 | C | A | -0.0293183 | 0.00408097 | 6.78E-13 |
| rs77244849 | 17 | C | T | -0.00875261 | 0.00148835 | 4.09E-09 |
| rs9891030 | 17 | A | G | 0.00993826 | 0.00159406 | 4.54E-10 |
| rs71352934 | 18 | C | A | -0.0163114 | 0.00273609 | 2.50E-09 |
| rs8092347 | 18 | G | A | 0.00812063 | 0.00141115 | 8.69E-09 |
| rs197156 | 19 | G | A | -0.00925016 | 0.00145381 | 1.99E-10 |
| rs1035941 | 19 | A | G | 0.0110148 | 0.00153309 | 6.75E-13 |
| rs4804413 | 19 | T | C | 0.00948499 | 0.00138516 | 7.53E-12 |
| rs116843064 | 19 | A | G | -0.108878 | 0.00491157 | 8.79E-109 |
| rs57192995 | 19 | C | G | -0.019903 | 0.00302387 | 4.65E-11 |
| rs58542926 | 19 | T | C | -0.0520456 | 0.00257716 | 1.26E-90 |
| rs188247550 | 19 | T | C | -0.0644712 | 0.00642107 | 1.02E-23 |
| rs62102718 | 19 | T | A | 0.0115887 | 0.00152691 | 3.22E-14 |
| rs58895965 | 19 | A | C | 0.0127238 | 0.00180395 | 1.75E-12 |
| rs541012177 | 19 | T | G | 0.0242543 | 0.00353606 | 6.94E-12 |
| rs41290102 | 19 | T | C | -0.0331177 | 0.00588779 | 1.86E-08 |
| rs419925 | 19 | C | G | -0.0130424 | 0.00149941 | 3.39E-18 |
| rs483082 | 19 | T | G | 0.0446753 | 0.00161686 | 8.09E-168 |
| rs79429216 | 19 | A | G | 0.0378212 | 0.0062985 | 1.92E-09 |
| rs146390218 | 19 | G | A | 0.0355235 | 0.00435052 | 3.22E-16 |
| rs62132802 | 19 | T | C | -0.00911832 | 0.00150245 | 1.29E-09 |
| rs12610709 | 19 | A | G | 0.013994 | 0.00183258 | 2.24E-14 |
| rs2207132 | 20 | A | G | 0.0283225 | 0.00387832 | 2.83E-13 |
| rs2250900 | 20 | T | C | 0.00899617 | 0.00163964 | 4.10E-08 |
| rs6073958 | 20 | C | T | 0.0274094 | 0.00172428 | 7.14E-57 |
| rs4812995 | 20 | C | T | 0.0091331 | 0.00161739 | 1.64E-08 |
| rs6066138 | 20 | A | G | -0.00850564 | 0.00152344 | 2.36E-08 |
| rs6090040 | 20 | A | C | 0.0089174 | 0.00138822 | 1.33E-10 |
| rs2277844 | 22 | G | A | -0.00908433 | 0.00138503 | 5.43E-11 |

**Supplementary Table 2. Characteristics of the SNPs associated with genus-level gut microbes.**

| Bacterium | SNP | Effect allele | Other allele | EAF | Beta | SE | *P*-value |
| --- | --- | --- | --- | --- | --- | --- | --- |
| *Clostridium innocuum* | rs10074000 | T | C | 0.403 | -0.103 | 0.023 | 7.00E-06 |
| *Clostridium innocuum* | rs10506058 | A | G | 0.406 | 0.1 | 0.022 | 8.92E-06 |
| *Clostridium innocuum* | rs1942371 | G | A | 0.121 | -0.158 | 0.034 | 4.06E-06 |
| *Clostridium innocuum* | rs40656 | C | T | 0.166 | 0.143 | 0.031 | 8.62E-06 |
| *Clostridium innocuum* | rs4869133 | G | A | 0.083 | -0.181 | 0.041 | 7.24E-06 |
| *Clostridium innocuum* | rs61267978 | T | C | 0.133 | 0.147 | 0.032 | 5.59E-06 |
| *Clostridium innocuum* | rs6577484 | G | A | 0.133 | 0.16 | 0.036 | 8.41E-06 |
| *Clostridium innocuum* | rs6890185 | T | C | 0.328 | 0.113 | 0.023 | 1.12E-06 |
| *Clostridium innocuum* | rs77845139 | A | G | 0.262 | -0.115 | 0.026 | 8.41E-06 |
| *Eubacterium brachy* | rs112617308 | T | C | 0.1 | -0.171 | 0.036 | 2.38E-06 |
| *Eubacterium brachy* | rs13139592 | T | C | 0.153 | -0.146 | 0.033 | 7.97E-06 |
| *Eubacterium brachy* | rs1384962 | A | G | 0.224 | 0.121 | 0.027 | 6.99E-06 |
| *Eubacterium brachy* | rs2913110 | C | T | 0.414 | 0.105 | 0.023 | 4.56E-06 |
| *Eubacterium brachy* | rs4862235 | G | A | 0.415 | 0.105 | 0.023 | 3.73E-06 |
| *Eubacterium brachy* | rs62348779 | T | C | 0.076 | -0.201 | 0.043 | 3.78E-06 |
| *Eubacterium brachy* | rs6591893 | G | A | 0.313 | 0.108 | 0.024 | 7.34E-06 |
| *Eubacterium brachy* | rs720439 | A | G | 0.259 | -0.112 | 0.025 | 7.03E-06 |
| *Eubacterium brachy* | rs73199919 | T | C | 0.066 | -0.237 | 0.053 | 8.16E-06 |
| *Eubacterium coprostanoligenes* | rs1020520 | T | G | 0.192 | -0.059 | 0.013 | 8.89E-06 |
| *Eubacterium coprostanoligenes* | rs10444197 | A | G | 0.329 | -0.051 | 0.011 | 5.98E-06 |
| *Eubacterium coprostanoligenes* | rs11052069 | T | C | 0.436 | 0.048 | 0.011 | 9.38E-06 |
| *Eubacterium coprostanoligenes* | rs11720857 | C | T | 0.178 | 0.063 | 0.014 | 9.26E-06 |
| *Eubacterium coprostanoligenes* | rs12906958 | C | T | 0.317 | -0.053 | 0.012 | 4.35E-06 |
| *Eubacterium coprostanoligenes* | rs17159861 | C | T | 0.119 | 0.096 | 0.017 | 1.04E-08 |
| *Eubacterium coprostanoligenes* | rs2644213 | G | A | 0.236 | 0.054 | 0.012 | 9.86E-06 |
| *Eubacterium coprostanoligenes* | rs4076415 | T | G | 0.443 | 0.052 | 0.011 | 1.99E-06 |
| *Eubacterium coprostanoligenes* | rs62024432 | C | T | 0.101 | -0.077 | 0.017 | 7.50E-06 |
| *Eubacterium coprostanoligenes* | rs6762473 | C | A | 0.342 | 0.052 | 0.011 | 4.26E-06 |
| *Eubacterium coprostanoligenes* | rs76898927 | G | A | 0.049 | 0.123 | 0.027 | 4.79E-06 |
| *Eubacterium coprostanoligenes* | rs79895140 | T | C | 0.207 | -0.064 | 0.014 | 8.62E-06 |
| *Eubacterium coprostanoligenes* | rs9648214 | T | C | 0.131 | -0.083 | 0.016 | 2.52E-07 |
| *Eubacterium eligens* | rs12719051 | A | G | 0.112 | 0.092 | 0.021 | 7.12E-06 |
| *Eubacterium eligens* | rs182318 | G | A | 0.081 | -0.082 | 0.02 | 8.40E-06 |
| *Eubacterium eligens* | rs2200429 | A | G | 0.088 | -0.089 | 0.02 | 5.30E-06 |
| *Eubacterium eligens* | rs265534 | T | G | 0.436 | -0.056 | 0.012 | 2.27E-06 |
| *Eubacterium eligens* | rs4583233 | A | C | 0.345 | 0.067 | 0.013 | 2.84E-07 |
| *Eubacterium eligens* | rs56080211 | C | T | 0.069 | 0.123 | 0.028 | 9.14E-06 |
| *Eubacterium eligens* | rs6923695 | T | G | 0.079 | 0.103 | 0.023 | 4.87E-06 |
| *Eubacterium fissicatena* | rs10147907 | T | G | 0.087 | 0.172 | 0.04 | 8.27E-06 |
| *Eubacterium fissicatena* | rs11818408 | G | A | 0.457 | 0.106 | 0.024 | 8.20E-06 |
| *Eubacterium fissicatena* | rs11876297 | T | C | 0.263 | 0.131 | 0.028 | 2.67E-06 |
| *Eubacterium fissicatena* | rs151257695 | A | G | 0.071 | 0.21 | 0.045 | 3.10E-06 |
| *Eubacterium fissicatena* | rs1768152 | T | C | 0.148 | 0.139 | 0.032 | 8.70E-06 |
| *Eubacterium fissicatena* | rs2733072 | G | A | 0.436 | 0.11 | 0.023 | 1.49E-06 |
| *Eubacterium fissicatena* | rs3771393 | C | T | 0.264 | 0.131 | 0.027 | 7.38E-07 |
| *Eubacterium fissicatena* | rs6934739 | A | G | 0.286 | 0.111 | 0.025 | 9.75E-06 |
| *Eubacterium fissicatena* | rs7104872 | G | A | 0.186 | 0.139 | 0.029 | 2.73E-06 |
| *Eubacterium hallii* | rs10501370 | C | T | 0.049 | -0.116 | 0.025 | 5.42E-06 |
| *Eubacterium hallii* | rs10798999 | C | T | 0.223 | 0.06 | 0.013 | 2.61E-06 |
| *Eubacterium hallii* | rs10808115 | A | C | 0.469 | -0.05 | 0.011 | 4.42E-06 |
| *Eubacterium hallii* | rs117748144 | T | C | 0.056 | -0.127 | 0.029 | 7.86E-06 |
| *Eubacterium hallii* | rs13116360 | T | C | 0.056 | 0.154 | 0.03 | 2.94E-07 |
| *Eubacterium hallii* | rs138531890 | A | G | 0.055 | 0.153 | 0.035 | 5.43E-06 |
| *Eubacterium hallii* | rs17074066 | T | C | 0.088 | -0.081 | 0.019 | 9.35E-06 |
| *Eubacterium hallii* | rs17474256 | G | A | 0.099 | 0.081 | 0.018 | 9.45E-06 |
| *Eubacterium hallii* | rs28584818 | A | G | 0.053 | 0.126 | 0.027 | 4.43E-06 |
| *Eubacterium hallii* | rs630939 | C | T | 0.405 | -0.051 | 0.011 | 9.16E-06 |
| *Eubacterium hallii* | rs6550770 | T | C | 0.055 | -0.198 | 0.044 | 4.82E-06 |
| *Eubacterium hallii* | rs74018587 | C | T | 0.033 | 0.209 | 0.044 | 3.70E-06 |
| *Eubacterium hallii* | rs78056098 | G | T | 0.353 | -0.051 | 0.011 | 8.29E-06 |
| *Eubacterium hallii* | rs949971 | T | G | 0.38 | -0.054 | 0.012 | 3.29E-06 |
| *Eubacterium nodatum* | rs10263623 | C | T | 0.103 | 0.193 | 0.044 | 8.91E-06 |
| *Eubacterium nodatum* | rs11006576 | A | G | 0.491 | -0.11 | 0.025 | 7.99E-06 |
| *Eubacterium nodatum* | rs113893692 | C | T | 0.117 | -0.185 | 0.04 | 5.76E-06 |
| *Eubacterium nodatum* | rs34297067 | A | G | 0.154 | -0.187 | 0.034 | 6.60E-08 |
| *Eubacterium nodatum* | rs61841040 | G | T | 0.153 | 0.161 | 0.034 | 3.56E-06 |
| *Eubacterium nodatum* | rs6818880 | A | G | 0.459 | -0.11 | 0.025 | 7.83E-06 |
| *Eubacterium nodatum* | rs77910827 | C | T | 0.1 | 0.202 | 0.041 | 9.05E-07 |
| *Eubacterium nodatum* | rs7827125 | C | T | 0.285 | 0.122 | 0.027 | 7.17E-06 |
| *Eubacterium nodatum* | rs7880204 | T | C | 0.265 | -0.125 | 0.028 | 6.84E-06 |
| *Eubacterium nodatum* | rs9425984 | T | C | 0.234 | -0.13 | 0.029 | 7.21E-06 |
| *Eubacterium oxidoreducens* | rs12129908 | C | A | 0.425 | 0.089 | 0.02 | 5.80E-06 |
| *Eubacterium oxidoreducens* | rs12423772 | G | T | 0.122 | 0.141 | 0.03 | 2.63E-06 |
| *Eubacterium oxidoreducens* | rs2973294 | G | T | 0.436 | 0.092 | 0.02 | 2.39E-06 |
| *Eubacterium rectale* | rs10248854 | C | A | 0.358 | -0.053 | 0.011 | 4.21E-06 |
| *Eubacterium rectale* | rs10797540 | A | G | 0.435 | 0.05 | 0.011 | 3.53E-06 |
| *Eubacterium rectale* | rs143694765 | T | C | 0.1 | 0.087 | 0.02 | 9.75E-06 |
| *Eubacterium rectale* | rs2884897 | A | G | 0.046 | -0.129 | 0.029 | 6.44E-06 |
| *Eubacterium rectale* | rs314726 | T | C | 0.43 | 0.053 | 0.011 | 1.38E-06 |
| *Eubacterium rectale* | rs35398954 | A | G | 0.111 | -0.09 | 0.017 | 5.40E-07 |
| *Eubacterium rectale* | rs59427698 | A | G | 0.251 | -0.058 | 0.013 | 5.37E-06 |
| *Eubacterium ruminantium* | rs10131724 | A | C | 0.059 | -0.2 | 0.041 | 2.39E-06 |
| *Eubacterium ruminantium* | rs10923018 | G | A | 0.45 | 0.073 | 0.016 | 6.80E-06 |
| *Eubacterium ruminantium* | rs11637981 | G | T | 0.46 | -0.073 | 0.016 | 5.44E-06 |
| *Eubacterium ruminantium* | rs13025464 | T | C | 0.376 | -0.074 | 0.016 | 6.97E-06 |
| *Eubacterium ruminantium* | rs139749 | C | T | 0.341 | -0.085 | 0.017 | 8.59E-07 |
| *Eubacterium ruminantium* | rs16891896 | G | A | 0.052 | -0.175 | 0.039 | 2.38E-06 |
| *Eubacterium ruminantium* | rs17519472 | C | T | 0.147 | 0.108 | 0.023 | 4.70E-06 |
| *Eubacterium ruminantium* | rs209813 | G | A | 0.12 | -0.103 | 0.024 | 9.23E-06 |
| *Eubacterium ruminantium* | rs2116427 | A | G | 0.244 | 0.091 | 0.018 | 4.67E-07 |
| *Eubacterium ruminantium* | rs2229917 | A | G | 0.064 | 0.154 | 0.032 | 2.16E-06 |
| *Eubacterium ruminantium* | rs2418654 | C | T | 0.48 | -0.075 | 0.017 | 6.17E-06 |
| *Eubacterium ruminantium* | rs2817174 | C | T | 0.388 | -0.073 | 0.016 | 7.87E-06 |
| *Eubacterium ruminantium* | rs57340348 | T | C | 0.189 | -0.098 | 0.021 | 4.93E-06 |
| *Eubacterium ruminantium* | rs606117 | A | G | 0.31 | 0.083 | 0.018 | 4.82E-06 |
| *Eubacterium ruminantium* | rs6676699 | G | T | 0.201 | -0.089 | 0.02 | 6.38E-06 |
| *Eubacterium ruminantium* | rs7000472 | A | G | 0.403 | -0.076 | 0.017 | 4.07E-06 |
| *Eubacterium ruminantium* | rs72836424 | C | T | 0.087 | -0.14 | 0.03 | 2.62E-06 |
| *Eubacterium ruminantium* | rs73139629 | A | C | 0.118 | -0.115 | 0.025 | 5.36E-06 |
| *Eubacterium ventriosum* | rs11617697 | A | G | 0.048 | -0.143 | 0.029 | 7.22E-07 |
| *Eubacterium ventriosum* | rs12964517 | G | A | 0.325 | 0.059 | 0.012 | 2.07E-06 |
| *Eubacterium ventriosum* | rs13082419 | C | T | 0.155 | -0.072 | 0.016 | 9.56E-06 |
| *Eubacterium ventriosum* | rs16884680 | G | T | 0.093 | -0.091 | 0.019 | 1.74E-06 |
| *Eubacterium ventriosum* | rs35179274 | C | T | 0.237 | -0.063 | 0.014 | 5.76E-06 |
| *Eubacterium ventriosum* | rs3809430 | T | C | 0.375 | -0.055 | 0.012 | 3.55E-06 |
| *Eubacterium ventriosum* | rs57199565 | T | C | 0.202 | 0.078 | 0.016 | 7.97E-07 |
| *Eubacterium ventriosum* | rs66746423 | C | T | 0.143 | 0.075 | 0.016 | 6.11E-06 |
| *Eubacterium ventriosum* | rs6704822 | A | G | 0.139 | 0.074 | 0.017 | 6.62E-06 |
| *Eubacterium ventriosum* | rs72783037 | C | A | 0.202 | 0.066 | 0.014 | 6.55E-06 |
| *Eubacterium ventriosum* | rs73615400 | T | C | 0.09 | -0.096 | 0.019 | 9.54E-07 |
| *Eubacterium ventriosum* | rs73849225 | T | C | 0.065 | 0.098 | 0.022 | 5.21E-06 |
| *Eubacterium ventriosum* | rs78250280 | G | A | 0.157 | 0.075 | 0.016 | 3.36E-06 |
| *Eubacterium ventriosum* | rs876734 | C | T | 0.255 | -0.062 | 0.013 | 2.89E-06 |
| *Eubacterium ventriosum* | rs9316536 | T | G | 0.113 | -0.082 | 0.018 | 7.84E-06 |
| *Eubacterium xylanophilum* | rs10140184 | A | C | 0.423 | 0.058 | 0.013 | 4.96E-06 |
| *Eubacterium xylanophilum* | rs112176119 | C | T | 0.083 | -0.113 | 0.025 | 3.33E-06 |
| *Eubacterium xylanophilum* | rs13239072 | G | A | 0.261 | 0.069 | 0.014 | 1.82E-06 |
| *Eubacterium xylanophilum* | rs17830032 | G | A | 0.055 | -0.161 | 0.031 | 2.39E-07 |
| *Eubacterium xylanophilum* | rs1999224 | G | T | 0.096 | -0.095 | 0.02 | 3.75E-06 |
| *Eubacterium xylanophilum* | rs2213117 | T | G | 0.116 | 0.088 | 0.019 | 4.21E-06 |
| *Eubacterium xylanophilum* | rs75586835 | A | G | 0.062 | -0.114 | 0.026 | 9.39E-06 |
| *Ruminococcus gauvreauii* | rs12079579 | A | G | 0.088 | 0.096 | 0.021 | 5.04E-06 |
| *Ruminococcus gauvreauii* | rs12539819 | C | T | 0.098 | 0.111 | 0.024 | 4.49E-06 |
| *Ruminococcus gauvreauii* | rs1391597 | C | T | 0.412 | 0.059 | 0.012 | 1.86E-06 |
| *Ruminococcus gauvreauii* | rs2047242 | A | G | 0.328 | -0.068 | 0.013 | 2.46E-07 |
| *Ruminococcus gauvreauii* | rs2166943 | A | C | 0.421 | 0.057 | 0.012 | 5.28E-06 |
| *Ruminococcus gauvreauii* | rs289410 | G | A | 0.251 | -0.065 | 0.014 | 2.27E-06 |
| *Ruminococcus gauvreauii* | rs431418 | A | G | 0.098 | -0.095 | 0.021 | 5.54E-06 |
| *Ruminococcus gauvreauii* | rs73802842 | C | A | 0.156 | 0.074 | 0.017 | 7.48E-06 |
| *Ruminococcus gnavus* | rs11597105 | A | G | 0.142 | 0.115 | 0.025 | 6.95E-06 |
| *Ruminococcus gnavus* | rs11864644 | T | C | 0.089 | -0.14 | 0.032 | 5.01E-06 |
| *Ruminococcus gnavus* | rs12136548 | C | T | 0.313 | 0.09 | 0.02 | 3.10E-06 |
| *Ruminococcus gnavus* | rs12989336 | G | A | 0.337 | -0.085 | 0.019 | 7.12E-06 |
| *Ruminococcus gnavus* | rs13163520 | G | A | 0.174 | -0.127 | 0.023 | 5.61E-08 |
| *Ruminococcus gnavus* | rs3124783 | A | G | 0.144 | -0.116 | 0.025 | 2.67E-06 |
| *Ruminococcus gnavus* | rs4388134 | C | T | 0.229 | -0.09 | 0.02 | 9.12E-06 |
| *Ruminococcus gnavus* | rs62167033 | T | C | 0.068 | 0.185 | 0.04 | 3.50E-06 |
| *Ruminococcus gnavus* | rs78399089 | T | C | 0.083 | 0.144 | 0.033 | 6.63E-06 |
| *Ruminococcus gnavus* | rs934940 | A | C | 0.171 | -0.105 | 0.023 | 2.74E-06 |
| *Ruminococcus gnavus* | rs9872758 | T | C | 0.488 | 0.085 | 0.018 | 1.66E-06 |
| *Ruminococcus torques* | rs10904297 | A | G | 0.033 | -0.168 | 0.039 | 2.69E-06 |
| *Ruminococcus torques* | rs10967781 | C | A | 0.34 | 0.051 | 0.011 | 8.37E-06 |
| *Ruminococcus torques* | rs12434631 | A | G | 0.143 | 0.075 | 0.015 | 2.77E-06 |
| *Ruminococcus torques* | rs1475330 | T | C | 0.286 | 0.052 | 0.012 | 8.13E-06 |
| *Ruminococcus torques* | rs4073731 | T | C | 0.153 | 0.065 | 0.014 | 4.05E-06 |
| *Ruminococcus torques* | rs77034621 | T | G | 0.054 | -0.152 | 0.034 | 6.07E-06 |
| *Ruminococcus torques* | rs8080469 | G | A | 0.493 | 0.049 | 0.011 | 3.50E-06 |
| *Ruminococcus torques* | rs8141465 | A | G | 0.504 | 0.048 | 0.011 | 9.65E-06 |
| *Actinomyces* | rs34583783 | G | T | 0.107 | 0.127 | 0.027 | 4.49E-06 |
| *Actinomyces* | rs35011108 | A | G | 0.051 | 0.233 | 0.051 | 6.34E-06 |
| *Actinomyces* | rs4073240 | G | A | 0.389 | 0.075 | 0.017 | 7.94E-06 |
| *Actinomyces* | rs4146653 | G | A | 0.181 | 0.099 | 0.021 | 4.50E-06 |
| *Actinomyces* | rs71315246 | A | G | 0.158 | -0.097 | 0.022 | 9.83E-06 |
| *Actinomyces* | rs7915461 | T | C | 0.058 | 0.188 | 0.04 | 5.92E-06 |
| *Adlercreutzia* | rs11604400 | C | T | 0.115 | -0.103 | 0.023 | 9.74E-06 |
| *Adlercreutzia* | rs13231526 | C | A | 0.076 | 0.143 | 0.031 | 4.81E-06 |
| *Adlercreutzia* | rs2717140 | C | T | 0.106 | -0.119 | 0.025 | 2.05E-06 |
| *Adlercreutzia* | rs55719207 | G | A | 0.43 | -0.07 | 0.016 | 9.61E-06 |
| *Adlercreutzia* | rs6664405 | T | C | 0.168 | -0.095 | 0.021 | 5.23E-06 |
| *Adlercreutzia* | rs7680684 | C | T | 0.292 | -0.083 | 0.017 | 9.77E-07 |
| *Adlercreutzia* | rs9490822 | C | T | 0.46 | -0.073 | 0.016 | 2.54E-06 |
| *Adlercreutzia* | rs9915817 | T | C | 0.313 | 0.075 | 0.017 | 8.22E-06 |
| *Akkermansia* | rs111862613 | T | C | 0.171 | 0.091 | 0.02 | 3.39E-06 |
| *Akkermansia* | rs117107102 | A | G | 0.043 | 0.204 | 0.043 | 3.01E-06 |
| *Akkermansia* | rs11729256 | T | C | 0.258 | 0.075 | 0.015 | 6.58E-07 |
| *Akkermansia* | rs12908520 | G | A | 0.432 | 0.062 | 0.013 | 2.26E-06 |
| *Akkermansia* | rs2602429 | C | T | 0.218 | 0.075 | 0.016 | 2.72E-06 |
| *Akkermansia* | rs4242783 | G | A | 0.263 | 0.069 | 0.015 | 3.00E-06 |
| *Akkermansia* | rs61779207 | G | A | 0.183 | -0.076 | 0.017 | 6.32E-06 |
| *Akkermansia* | rs74542928 | T | C | 0.098 | 0.113 | 0.024 | 1.48E-06 |
| *Akkermansia* | rs9349825 | A | G | 0.264 | -0.07 | 0.015 | 2.60E-06 |
| *Akkermansia* | rs941682 | G | A | 0.273 | -0.063 | 0.014 | 9.17E-06 |
| *Alistipes* | rs1107244 | G | A | 0.085 | 0.076 | 0.017 | 3.59E-06 |
| *Alistipes* | rs11769002 | G | A | 0.398 | -0.053 | 0.011 | 1.45E-06 |
| *Alistipes* | rs11958296 | A | G | 0.076 | -0.098 | 0.022 | 9.30E-06 |
| *Alistipes* | rs12990744 | C | T | 0.125 | -0.078 | 0.017 | 8.21E-06 |
| *Alistipes* | rs1689282 | A | C | 0.333 | -0.052 | 0.011 | 5.28E-06 |
| *Alistipes* | rs2290844 | C | T | 0.076 | 0.081 | 0.019 | 9.10E-06 |
| *Alistipes* | rs2450745 | A | C | 0.089 | -0.081 | 0.018 | 7.12E-06 |
| *Alistipes* | rs2875322 | T | C | 0.209 | -0.058 | 0.013 | 8.78E-06 |
| *Alistipes* | rs34417064 | A | G | 0.499 | -0.048 | 0.011 | 7.01E-06 |
| *Alistipes* | rs4810359 | A | G | 0.159 | -0.065 | 0.015 | 7.50E-06 |
| *Alistipes* | rs62576416 | T | C | 0.4 | 0.049 | 0.011 | 7.50E-06 |
| *Alistipes* | rs67705352 | T | G | 0.376 | -0.053 | 0.011 | 1.65E-06 |
| *Alistipes* | rs7129639 | C | A | 0.375 | -0.052 | 0.011 | 1.78E-06 |
| *Allisonella* | rs1901739 | T | G | 0.466 | 0.116 | 0.025 | 3.59E-06 |
| *Allisonella* | rs35110698 | T | C | 0.165 | -0.146 | 0.032 | 5.72E-06 |
| *Allisonella* | rs35778461 | C | T | 0.195 | 0.147 | 0.03 | 1.21E-06 |
| *Allisonella* | rs594561 | C | T | 0.439 | 0.112 | 0.025 | 9.41E-06 |
| *Allisonella* | rs602075 | A | G | 0.261 | 0.169 | 0.03 | 3.57E-08 |
| *Allisonella* | rs6742198 | G | A | 0.206 | 0.149 | 0.032 | 3.35E-06 |
| *Allisonella* | rs76904847 | G | A | 0.188 | 0.149 | 0.033 | 6.09E-06 |
| *Allisonella* | rs7898615 | T | G | 0.126 | 0.168 | 0.037 | 8.87E-06 |
| *Alloprevotella* | rs12675596 | G | T | 0.242 | 0.146 | 0.029 | 9.64E-07 |
| *Alloprevotella* | rs2154444 | T | G | 0.198 | 0.138 | 0.031 | 8.37E-06 |
| *Alloprevotella* | rs34619204 | G | A | 0.202 | -0.156 | 0.034 | 8.84E-06 |
| *Alloprevotella* | rs4364940 | A | G | 0.262 | 0.126 | 0.028 | 8.58E-06 |
| *Alloprevotella* | rs4680035 | A | G | 0.53 | -0.12 | 0.026 | 4.99E-06 |
| *Alloprevotella* | rs58212166 | A | G | 0.137 | -0.162 | 0.036 | 7.94E-06 |
| *Anaerofilum* | rs10794359 | T | C | 0.462 | -0.095 | 0.02 | 2.23E-06 |
| *Anaerofilum* | rs1563175 | A | C | 0.415 | 0.092 | 0.02 | 5.54E-06 |
| *Anaerofilum* | rs17012738 | T | G | 0.437 | 0.09 | 0.02 | 7.24E-06 |
| *Anaerofilum* | rs17096874 | C | T | 0.168 | -0.126 | 0.027 | 2.86E-06 |
| *Anaerofilum* | rs4244069 | G | A | 0.107 | -0.147 | 0.033 | 9.81E-06 |
| *Anaerofilum* | rs4506496 | G | A | 0.33 | 0.103 | 0.021 | 1.49E-06 |
| *Anaerofilum* | rs712981 | A | C | 0.406 | 0.101 | 0.02 | 6.83E-07 |
| *Anaerofilum* | rs79598899 | C | T | 0.087 | 0.183 | 0.036 | 3.75E-07 |
| *Anaerofilum* | rs816292 | T | C | 0.266 | -0.113 | 0.022 | 2.64E-07 |
| *Anaerofilum* | rs9299345 | T | C | 0.132 | -0.136 | 0.03 | 8.04E-06 |
| *Anaerostipes* | rs10502061 | A | G | 0.084 | 0.084 | 0.019 | 7.94E-06 |
| *Anaerostipes* | rs2014785 | T | C | 0.416 | 0.052 | 0.011 | 4.68E-06 |
| *Anaerostipes* | rs2396460 | T | C | 0.46 | -0.051 | 0.011 | 2.91E-06 |
| *Anaerostipes* | rs2804244 | A | G | 0.47 | -0.053 | 0.011 | 2.04E-06 |
| *Anaerostipes* | rs3900776 | G | A | 0.071 | -0.11 | 0.024 | 2.75E-06 |
| *Anaerostipes* | rs60983350 | G | A | 0.399 | -0.054 | 0.012 | 4.42E-06 |
| *Anaerostipes* | rs62157625 | T | C | 0.115 | 0.089 | 0.019 | 1.45E-06 |
| *Anaerostipes* | rs62215703 | G | A | 0.207 | 0.064 | 0.014 | 1.98E-06 |
| *Anaerostipes* | rs6474958 | A | G | 0.415 | -0.05 | 0.011 | 6.74E-06 |
| *Anaerostipes* | rs7193624 | C | T | 0.149 | 0.075 | 0.015 | 5.35E-07 |
| *Anaerostipes* | rs78735375 | A | C | 0.055 | -0.137 | 0.031 | 5.33E-06 |
| *Anaerotruncus* | rs10150232 | A | G | 0.283 | 0.057 | 0.012 | 6.68E-06 |
| *Anaerotruncus* | rs11018566 | A | G | 0.043 | -0.156 | 0.037 | 6.14E-06 |
| *Anaerotruncus* | rs115414803 | A | C | 0.049 | -0.144 | 0.032 | 6.83E-06 |
| *Anaerotruncus* | rs1272208 | G | T | 0.239 | -0.061 | 0.013 | 4.28E-06 |
| *Anaerotruncus* | rs1431492 | C | T | 0.171 | -0.065 | 0.015 | 7.36E-06 |
| *Anaerotruncus* | rs17734739 | T | C | 0.178 | 0.066 | 0.015 | 7.43E-06 |
| *Anaerotruncus* | rs34449434 | A | C | 0.446 | -0.05 | 0.011 | 9.85E-06 |
| *Anaerotruncus* | rs4669806 | G | T | 0.27 | 0.058 | 0.012 | 2.42E-06 |
| *Anaerotruncus* | rs6494922 | A | G | 0.078 | 0.09 | 0.02 | 6.62E-06 |
| *Anaerotruncus* | rs6563550 | T | C | 0.085 | 0.088 | 0.018 | 2.35E-07 |
| *Anaerotruncus* | rs9347879 | T | C | 0.468 | 0.051 | 0.011 | 4.22E-06 |
| *Bacteroides* | rs11585893 | A | G | 0.143 | -0.074 | 0.015 | 1.80E-06 |
| *Bacteroides* | rs1340391 | T | C | 0.197 | -0.059 | 0.013 | 6.73E-06 |
| *Bacteroides* | rs17619981 | T | G | 0.104 | 0.088 | 0.019 | 2.69E-06 |
| *Bacteroides* | rs2023437 | T | C | 0.112 | -0.078 | 0.017 | 5.02E-06 |
| *Bacteroides* | rs66474973 | G | T | 0.118 | 0.081 | 0.016 | 6.81E-07 |
| *Bacteroides* | rs6795673 | C | T | 0.447 | 0.054 | 0.011 | 3.38E-07 |
| *Bacteroides* | rs9507307 | C | T | 0.197 | 0.06 | 0.013 | 2.13E-06 |
| *Barnesiella* | rs11155559 | T | C | 0.091 | 0.096 | 0.021 | 8.92E-06 |
| *Barnesiella* | rs113258194 | A | G | 0.078 | 0.099 | 0.021 | 7.31E-06 |
| *Barnesiella* | rs13242616 | T | C | 0.402 | -0.058 | 0.012 | 2.29E-06 |
| *Barnesiella* | rs199035 | G | A | 0.531 | 0.056 | 0.012 | 3.00E-06 |
| *Barnesiella* | rs2276875 | A | G | 0.261 | -0.07 | 0.014 | 4.65E-07 |
| *Barnesiella* | rs2428166 | G | A | 0.056 | -0.166 | 0.034 | 8.51E-07 |
| *Barnesiella* | rs35177866 | A | G | 0.134 | 0.092 | 0.019 | 2.95E-06 |
| *Barnesiella* | rs60316894 | C | T | 0.059 | -0.121 | 0.025 | 1.19E-06 |
| *Barnesiella* | rs62251337 | A | G | 0.222 | -0.069 | 0.015 | 4.24E-06 |
| *Barnesiella* | rs72684847 | T | C | 0.062 | -0.114 | 0.025 | 6.76E-06 |
| *Barnesiella* | rs76181748 | C | T | 0.142 | -0.078 | 0.017 | 6.78E-06 |
| *Barnesiella* | rs77455852 | T | G | 0.122 | -0.089 | 0.02 | 3.16E-06 |
| *Barnesiella* | rs79795328 | A | G | 0.135 | -0.082 | 0.018 | 4.23E-06 |
| *Bifidobacterium* | rs12022129 | G | A | 0.204 | 0.062 | 0.014 | 8.00E-06 |
| *Bifidobacterium* | rs2491158 | G | A | 0.182 | 0.071 | 0.016 | 8.05E-06 |
| *Bifidobacterium* | rs2686790 | T | C | 0.144 | 0.071 | 0.016 | 7.50E-06 |
| *Bifidobacterium* | rs540489 | T | G | 0.235 | -0.064 | 0.014 | 5.19E-06 |
| *Bifidobacterium* | rs55888705 | A | G | 0.364 | 0.055 | 0.012 | 6.67E-06 |
| *Bifidobacterium* | rs5746486 | T | C | 0.357 | -0.054 | 0.012 | 9.00E-06 |
| *Bifidobacterium* | rs62181700 | G | A | 0.28 | -0.062 | 0.013 | 2.17E-06 |
| *Bifidobacterium* | rs7322849 | T | C | 0.08 | 0.112 | 0.02 | 1.08E-08 |
| *Bifidobacterium* | rs73797465 | T | G | 0.068 | -0.095 | 0.021 | 4.38E-06 |
| *Bifidobacterium* | rs75344046 | C | T | 0.042 | 0.232 | 0.051 | 4.86E-06 |
| *Bifidobacterium* | rs857444 | C | T | 0.359 | 0.056 | 0.012 | 3.57E-06 |
| *Bilophila* | rs11069458 | T | C | 0.174 | -0.068 | 0.016 | 7.72E-06 |
| *Bilophila* | rs1241171 | G | A | 0.206 | -0.069 | 0.015 | 4.24E-06 |
| *Bilophila* | rs1969927 | G | A | 0.412 | 0.056 | 0.013 | 9.07E-06 |
| *Bilophila* | rs2728491 | G | T | 0.274 | -0.063 | 0.014 | 6.33E-06 |
| *Bilophila* | rs3827020 | C | T | 0.179 | 0.077 | 0.016 | 1.79E-06 |
| *Bilophila* | rs4798126 | G | A | 0.144 | 0.073 | 0.017 | 7.15E-06 |
| *Bilophila* | rs542415 | T | C | 0.338 | -0.061 | 0.013 | 4.71E-06 |
| *Bilophila* | rs60178956 | G | A | 0.262 | -0.062 | 0.014 | 8.06E-06 |
| *Bilophila* | rs6793291 | C | A | 0.095 | 0.113 | 0.024 | 3.11E-06 |
| *Bilophila* | rs72676854 | T | C | 0.07 | 0.123 | 0.027 | 5.62E-06 |
| *Bilophila* | rs7802841 | C | A | 0.308 | 0.067 | 0.014 | 1.77E-06 |
| *Bilophila* | rs9899990 | A | G | 0.083 | -0.103 | 0.023 | 9.07E-06 |
| *Blautia* | rs11149971 | C | T | 0.053 | 0.118 | 0.023 | 1.04E-06 |
| *Blautia* | rs117001700 | T | C | 0.053 | 0.196 | 0.044 | 8.84E-06 |
| *Blautia* | rs12453000 | C | T | 0.211 | 0.063 | 0.013 | 1.26E-06 |
| *Blautia* | rs2788271 | T | G | 0.168 | -0.058 | 0.013 | 7.16E-06 |
| *Blautia* | rs3005511 | A | G | 0.333 | 0.05 | 0.011 | 6.19E-06 |
| *Blautia* | rs4926264 | T | C | 0.072 | 0.083 | 0.018 | 5.10E-06 |
| *Blautia* | rs67794373 | C | T | 0.226 | 0.06 | 0.012 | 1.00E-06 |
| *Blautia* | rs72973581 | A | G | 0.064 | 0.125 | 0.027 | 1.74E-06 |
| *Blautia* | rs7860714 | A | G | 0.369 | -0.05 | 0.011 | 4.09E-06 |
| *Butyricicoccus* | rs10084203 | A | G | 0.249 | 0.055 | 0.012 | 8.59E-06 |
| *Butyricicoccus* | rs12585793 | T | C | 0.043 | -0.262 | 0.056 | 5.79E-06 |
| *Butyricicoccus* | rs2017189 | G | T | 0.434 | -0.051 | 0.011 | 3.87E-06 |
| *Butyricicoccus* | rs4962426 | G | T | 0.217 | 0.061 | 0.014 | 7.38E-06 |
| *Butyricicoccus* | rs56221232 | T | C | 0.118 | 0.083 | 0.017 | 7.62E-07 |
| *Butyricicoccus* | rs62478070 | T | G | 0.052 | 0.224 | 0.049 | 5.94E-06 |
| *Butyricicoccus* | rs7322368 | T | C | 0.095 | 0.082 | 0.018 | 5.52E-06 |
| *Butyricimonas* | rs11228830 | A | G | 0.072 | 0.135 | 0.03 | 6.55E-06 |
| *Butyricimonas* | rs113054641 | G | A | 0.067 | -0.145 | 0.027 | 1.74E-07 |
| *Butyricimonas* | rs12304031 | G | A | 0.134 | -0.086 | 0.02 | 6.70E-06 |
| *Butyricimonas* | rs12458763 | A | C | 0.075 | 0.122 | 0.027 | 6.37E-06 |
| *Butyricimonas* | rs1862649 | G | A | 0.087 | 0.113 | 0.025 | 4.76E-06 |
| *Butyricimonas* | rs2114713 | G | T | 0.417 | 0.063 | 0.014 | 6.88E-06 |
| *Butyricimonas* | rs62390301 | T | C | 0.195 | -0.087 | 0.017 | 7.42E-07 |
| *Butyricimonas* | rs7083431 | A | C | 0.353 | 0.07 | 0.014 | 8.85E-07 |
| *Butyricimonas* | rs71428626 | G | T | 0.068 | -0.133 | 0.029 | 4.80E-06 |
| *Butyricimonas* | rs78453362 | A | G | 0.053 | -0.149 | 0.033 | 4.06E-06 |
| *Butyricimonas* | rs9657374 | C | T | 0.303 | 0.068 | 0.015 | 4.50E-06 |
| *Butyrivibrio* | rs1007475 | G | T | 0.309 | 0.118 | 0.026 | 7.92E-06 |
| *Butyrivibrio* | rs11761679 | T | C | 0.17 | 0.155 | 0.032 | 2.20E-06 |
| *Butyrivibrio* | rs142855850 | A | G | 0.107 | 0.205 | 0.046 | 6.86E-06 |
| *Butyrivibrio* | rs16934069 | T | C | 0.203 | -0.134 | 0.03 | 8.86E-06 |
| *Butyrivibrio* | rs16941336 | C | T | 0.267 | 0.127 | 0.027 | 1.53E-06 |
| *Butyrivibrio* | rs4537857 | T | C | 0.295 | -0.125 | 0.026 | 1.80E-06 |
| *Butyrivibrio* | rs486484 | A | G | 0.487 | -0.108 | 0.024 | 6.61E-06 |
| *Butyrivibrio* | rs4928024 | A | G | 0.095 | -0.175 | 0.039 | 8.19E-06 |
| *Butyrivibrio* | rs72723662 | C | T | 0.095 | 0.224 | 0.045 | 7.86E-07 |
| *Butyrivibrio* | rs74622183 | A | G | 0.08 | -0.201 | 0.043 | 2.46E-06 |
| *Butyrivibrio* | rs77356209 | T | C | 0.064 | 0.217 | 0.048 | 6.66E-06 |
| *Butyrivibrio* | rs7752361 | A | G | 0.441 | -0.119 | 0.024 | 7.69E-07 |
| *Butyrivibrio* | rs7763512 | G | A | 0.408 | 0.12 | 0.025 | 3.11E-06 |
| *Candidatus Soleaferrea* | rs10108780 | A | G | 0.293 | -0.093 | 0.02 | 3.64E-06 |
| *Candidatus Soleaferrea* | rs10809135 | T | C | 0.516 | 0.083 | 0.018 | 5.47E-06 |
| *Candidatus Soleaferrea* | rs36155147 | C | T | 0.355 | 0.105 | 0.024 | 5.41E-06 |
| *Candidatus Soleaferrea* | rs4678258 | T | C | 0.224 | 0.099 | 0.022 | 5.53E-06 |
| *Candidatus Soleaferrea* | rs61825792 | T | C | 0.196 | 0.112 | 0.023 | 1.37E-06 |
| *Candidatus Soleaferrea* | rs6494306 | A | G | 0.225 | -0.097 | 0.021 | 5.80E-06 |
| *Candidatus Soleaferrea* | rs9973954 | A | G | 0.287 | 0.089 | 0.02 | 5.95E-06 |
| *Catenibacterium* | rs12404911 | C | T | 0.192 | 0.141 | 0.03 | 2.80E-06 |
| *Catenibacterium* | rs212393 | G | A | 0.318 | -0.135 | 0.029 | 3.62E-06 |
| *Catenibacterium* | rs77285108 | G | A | 0.163 | -0.162 | 0.035 | 3.63E-06 |
| *Catenibacterium* | rs7742829 | C | T | 0.402 | 0.114 | 0.025 | 5.61E-06 |
| *Christensenellaceae R-7 group* | rs10461257 | A | G | 0.271 | -0.055 | 0.012 | 6.51E-06 |
| *Christensenellaceae R-7 group* | rs17081797 | A | G | 0.074 | -0.09 | 0.02 | 3.34E-06 |
| *Christensenellaceae R-7 group* | rs60954665 | T | G | 0.46 | 0.05 | 0.011 | 7.13E-06 |
| *Christensenellaceae R-7 group* | rs62467127 | C | T | 0.055 | 0.114 | 0.025 | 3.25E-06 |
| *Christensenellaceae R-7 group* | rs73952017 | C | T | 0.097 | -0.086 | 0.019 | 8.46E-06 |
| *Christensenellaceae R-7 group* | rs79150079 | C | A | 0.059 | 0.122 | 0.027 | 9.42E-06 |
| *Christensenellaceae R-7 group* | rs892686 | A | G | 0.474 | 0.051 | 0.011 | 3.97E-06 |
| *Clostridiumsensustricto1* | rs11264403 | G | A | 0.047 | -0.139 | 0.033 | 7.76E-06 |
| *Clostridiumsensustricto1* | rs115807074 | A | G | 0.046 | -0.227 | 0.049 | 4.32E-06 |
| *Clostridiumsensustricto1* | rs116847295 | C | T | 0.06 | 0.11 | 0.025 | 4.58E-06 |
| *Clostridiumsensustricto1* | rs12341505 | G | A | 0.14 | 0.081 | 0.018 | 4.82E-06 |
| *Clostridiumsensustricto1* | rs2795528 | G | A | 0.057 | -0.184 | 0.039 | 2.72E-06 |
| *Clostridiumsensustricto1* | rs2817172 | C | T | 0.37 | 0.058 | 0.012 | 2.77E-06 |
| *Collinsella* | rs10890671 | T | C | 0.485 | -0.054 | 0.012 | 6.52E-06 |
| *Collinsella* | rs1496626 | T | C | 0.134 | -0.072 | 0.016 | 6.78E-06 |
| *Collinsella* | rs149807560 | C | A | 0.074 | -0.104 | 0.024 | 7.10E-06 |
| *Collinsella* | rs2103510 | G | A | 0.133 | 0.079 | 0.017 | 2.42E-06 |
| *Collinsella* | rs62448871 | C | A | 0.428 | -0.054 | 0.012 | 6.78E-06 |
| *Collinsella* | rs73052258 | G | A | 0.098 | 0.093 | 0.02 | 1.72E-06 |
| *Collinsella* | rs75672793 | A | G | 0.079 | -0.109 | 0.024 | 6.14E-06 |
| *Collinsella* | rs9541268 | C | A | 0.119 | 0.096 | 0.02 | 8.79E-07 |
| *Coprobacter* | rs11532348 | C | T | 0.21 | -0.104 | 0.023 | 5.71E-06 |
| *Coprobacter* | rs12996055 | A | C | 0.268 | 0.092 | 0.021 | 8.08E-06 |
| *Coprobacter* | rs143662916 | C | T | 0.059 | 0.253 | 0.054 | 3.07E-06 |
| *Coprobacter* | rs213863 | C | T | 0.284 | -0.089 | 0.019 | 2.35E-06 |
| *Coprobacter* | rs28402691 | T | C | 0.139 | 0.111 | 0.025 | 9.56E-06 |
| *Coprobacter* | rs3828477 | G | T | 0.249 | -0.091 | 0.02 | 2.89E-06 |
| *Coprobacter* | rs72821405 | T | C | 0.088 | -0.147 | 0.032 | 4.76E-06 |
| *Coprococcus1* | rs1010560 | C | A | 0.303 | 0.058 | 0.012 | 1.96E-06 |
| *Coprococcus1* | rs12794898 | G | T | 0.085 | 0.09 | 0.02 | 4.92E-06 |
| *Coprococcus1* | rs1519491 | T | C | 0.414 | 0.05 | 0.011 | 8.95E-06 |
| *Coprococcus1* | rs1576241 | A | G | 0.473 | -0.051 | 0.011 | 3.33E-06 |
| *Coprococcus1* | rs2907920 | A | G | 0.282 | 0.056 | 0.013 | 7.65E-06 |
| *Coprococcus1* | rs56405618 | A | G | 0.1 | -0.09 | 0.019 | 1.57E-06 |
| *Coprococcus1* | rs73031725 | T | C | 0.043 | 0.168 | 0.036 | 1.98E-06 |
| *Coprococcus1* | rs73167075 | T | C | 0.237 | 0.057 | 0.013 | 8.57E-06 |
| *Coprococcus1* | rs74101919 | T | C | 0.183 | -0.072 | 0.014 | 1.03E-06 |
| *Coprococcus1* | rs946513 | C | T | 0.063 | 0.206 | 0.046 | 8.62E-06 |
| *Coprococcus2* | rs10070053 | A | G | 0.409 | 0.059 | 0.014 | 7.65E-06 |
| *Coprococcus2* | rs12634070 | T | C | 0.215 | 0.074 | 0.016 | 9.95E-06 |
| *Coprococcus2* | rs2482516 | C | T | 0.205 | 0.075 | 0.016 | 4.72E-06 |
| *Coprococcus2* | rs35890118 | A | G | 0.28 | -0.067 | 0.015 | 8.26E-06 |
| *Coprococcus2* | rs61823518 | A | C | 0.106 | -0.096 | 0.022 | 6.68E-06 |
| *Coprococcus2* | rs6677933 | C | T | 0.22 | -0.08 | 0.016 | 1.19E-06 |
| *Coprococcus2* | rs72680320 | T | C | 0.437 | -0.065 | 0.014 | 2.27E-06 |
| *Coprococcus2* | rs9426473 | A | G | 0.214 | 0.073 | 0.016 | 6.31E-06 |
| *Coprococcus3* | rs10810043 | A | G | 0.374 | 0.052 | 0.012 | 9.27E-06 |
| *Coprococcus3* | rs11077359 | T | C | 0.209 | -0.065 | 0.015 | 9.64E-06 |
| *Coprococcus3* | rs11080344 | C | T | 0.452 | 0.052 | 0.011 | 4.79E-06 |
| *Coprococcus3* | rs13247359 | G | A | 0.491 | 0.051 | 0.011 | 7.33E-06 |
| *Coprococcus3* | rs13394391 | C | T | 0.17 | -0.071 | 0.015 | 2.20E-06 |
| *Coprococcus3* | rs178271 | T | C | 0.058 | 0.145 | 0.029 | 7.81E-07 |
| *Coprococcus3* | rs4575475 | G | A | 0.241 | 0.062 | 0.014 | 7.04E-06 |
| *Coprococcus3* | rs7521171 | G | A | 0.28 | -0.06 | 0.013 | 4.32E-06 |
| *Coprococcus3* | rs8100692 | T | C | 0.427 | 0.058 | 0.011 | 4.16E-07 |
| *Defluviitaleaceae UCG-011* | rs112893842 | T | C | 0.127 | 0.114 | 0.023 | 1.45E-06 |
| *Defluviitaleaceae UCG-011* | rs1582238 | T | C | 0.335 | 0.081 | 0.017 | 1.57E-06 |
| *Defluviitaleaceae UCG-011* | rs2892880 | G | A | 0.246 | 0.082 | 0.018 | 6.83E-06 |
| *Defluviitaleaceae UCG-011* | rs4677103 | A | G | 0.199 | 0.098 | 0.02 | 9.60E-07 |
| *Defluviitaleaceae UCG-011* | rs55658617 | T | C | 0.078 | 0.174 | 0.036 | 2.15E-06 |
| *Defluviitaleaceae UCG-011* | rs72731813 | C | T | 0.085 | -0.147 | 0.029 | 4.33E-07 |
| *Defluviitaleaceae UCG-011* | rs9608282 | T | G | 0.077 | 0.143 | 0.03 | 2.52E-06 |
| *Defluviitaleaceae UCG-011* | rs9725395 | A | G | 0.081 | -0.138 | 0.03 | 3.52E-06 |
| *Desulfovibrio* | rs12031543 | T | C | 0.062 | -0.127 | 0.028 | 6.55E-06 |
| *Desulfovibrio* | rs13066142 | G | A | 0.084 | 0.119 | 0.025 | 3.79E-06 |
| *Desulfovibrio* | rs16863365 | A | G | 0.129 | 0.109 | 0.023 | 1.79E-06 |
| *Desulfovibrio* | rs2032031 | A | G | 0.493 | -0.065 | 0.015 | 9.14E-06 |
| *Desulfovibrio* | rs2590913 | G | A | 0.081 | 0.154 | 0.034 | 6.65E-06 |
| *Desulfovibrio* | rs2853179 | C | T | 0.237 | 0.081 | 0.017 | 2.42E-06 |
| *Desulfovibrio* | rs4797774 | G | A | 0.054 | 0.213 | 0.047 | 5.64E-06 |
| *Desulfovibrio* | rs6580353 | T | C | 0.237 | 0.077 | 0.017 | 4.94E-06 |
| *Desulfovibrio* | rs72647089 | T | G | 0.098 | -0.107 | 0.024 | 8.30E-06 |
| *Dialister* | rs10138457 | T | C | 0.066 | -0.113 | 0.026 | 7.88E-06 |
| *Dialister* | rs10938938 | G | A | 0.192 | -0.077 | 0.017 | 7.37E-06 |
| *Dialister* | rs11071887 | T | C | 0.273 | 0.066 | 0.015 | 5.91E-06 |
| *Dialister* | rs11166701 | G | A | 0.508 | -0.066 | 0.013 | 5.51E-07 |
| *Dialister* | rs2314294 | T | C | 0.14 | 0.087 | 0.019 | 8.08E-06 |
| *Dialister* | rs2435610 | A | C | 0.284 | 0.065 | 0.014 | 5.93E-06 |
| *Dialister* | rs4747450 | C | A | 0.267 | 0.067 | 0.015 | 5.84E-06 |
| *Dialister* | rs4753063 | G | A | 0.449 | -0.06 | 0.013 | 4.86E-06 |
| *Dialister* | rs75416973 | A | G | 0.19 | 0.073 | 0.016 | 9.46E-06 |
| *Dialister* | rs764177 | C | A | 0.32 | -0.06 | 0.014 | 9.61E-06 |
| *Dialister* | rs76680460 | G | A | 0.057 | -0.161 | 0.036 | 8.19E-06 |
| *Dorea* | rs11150408 | T | G | 0.463 | 0.049 | 0.011 | 7.06E-06 |
| *Dorea* | rs12537781 | T | C | 0.261 | -0.056 | 0.013 | 9.15E-06 |
| *Dorea* | rs13279148 | G | A | 0.156 | 0.072 | 0.015 | 2.25E-06 |
| *Dorea* | rs1899291 | C | T | 0.129 | 0.07 | 0.015 | 4.57E-06 |
| *Dorea* | rs3005511 | A | G | 0.333 | 0.052 | 0.011 | 5.29E-06 |
| *Dorea* | rs345219 | T | G | 0.376 | -0.05 | 0.011 | 8.80E-06 |
| *Dorea* | rs3752849 | G | A | 0.039 | 0.164 | 0.037 | 7.68E-06 |
| *Dorea* | rs4793307 | C | T | 0.264 | 0.057 | 0.012 | 4.01E-06 |
| *Dorea* | rs62503162 | A | G | 0.09 | -0.097 | 0.019 | 7.47E-07 |
| *Eggerthella* | rs112205261 | T | C | 0.066 | -0.189 | 0.04 | 3.35E-06 |
| *Eggerthella* | rs13070736 | A | C | 0.142 | -0.121 | 0.027 | 7.62E-06 |
| *Eggerthella* | rs1784446 | G | A | 0.426 | 0.091 | 0.02 | 5.23E-06 |
| *Eggerthella* | rs2223081 | G | A | 0.257 | 0.103 | 0.022 | 3.89E-06 |
| *Eggerthella* | rs2240838 | A | G | 0.54 | 0.098 | 0.02 | 7.36E-07 |
| *Eggerthella* | rs2877457 | G | A | 0.32 | -0.093 | 0.021 | 9.03E-06 |
| *Eggerthella* | rs3851328 | T | G | 0.233 | -0.108 | 0.024 | 4.18E-06 |
| *Eggerthella* | rs4985746 | G | A | 0.22 | 0.111 | 0.025 | 5.71E-06 |
| *Eggerthella* | rs6430926 | C | T | 0.496 | 0.088 | 0.02 | 8.37E-06 |
| *Eggerthella* | rs67490567 | T | C | 0.198 | 0.108 | 0.025 | 8.94E-06 |
| *Eggerthella* | rs76663501 | C | T | 0.089 | 0.175 | 0.038 | 4.83E-06 |
| *Eisenbergiella* | rs11027642 | C | T | 0.124 | 0.129 | 0.028 | 4.92E-06 |
| *Eisenbergiella* | rs11079158 | T | C | 0.244 | 0.101 | 0.023 | 7.35E-06 |
| *Eisenbergiella* | rs11938607 | T | C | 0.227 | 0.098 | 0.022 | 8.22E-06 |
| *Eisenbergiella* | rs12257723 | A | C | 0.278 | -0.095 | 0.021 | 8.85E-06 |
| *Eisenbergiella* | rs12710729 | C | A | 0.347 | 0.089 | 0.02 | 9.84E-06 |
| *Eisenbergiella* | rs13258851 | A | G | 0.103 | 0.137 | 0.03 | 7.75E-06 |
| *Eisenbergiella* | rs1508033 | A | C | 0.362 | 0.092 | 0.02 | 3.23E-06 |
| *Eisenbergiella* | rs1553971 | T | G | 0.154 | 0.121 | 0.026 | 5.27E-06 |
| *Eisenbergiella* | rs2683098 | C | T | 0.215 | 0.107 | 0.023 | 2.24E-06 |
| *Eisenbergiella* | rs3812426 | G | A | 0.229 | 0.106 | 0.022 | 2.72E-06 |
| *Eisenbergiella* | rs4462860 | G | A | 0.34 | 0.094 | 0.02 | 4.16E-06 |
| *Enterorhabdus* | rs10098492 | T | C | 0.09 | 0.132 | 0.029 | 6.41E-06 |
| *Enterorhabdus* | rs114731706 | T | G | 0.058 | 0.182 | 0.038 | 2.17E-06 |
| *Enterorhabdus* | rs2051957 | C | T | 0.256 | 0.084 | 0.019 | 8.90E-06 |
| *Enterorhabdus* | rs3017103 | A | G | 0.181 | 0.098 | 0.021 | 2.94E-06 |
| *Enterorhabdus* | rs73331712 | T | C | 0.047 | 0.262 | 0.055 | 4.85E-06 |
| *Enterorhabdus* | rs77655283 | G | A | 0.075 | 0.133 | 0.03 | 5.88E-06 |
| *Erysipelatoclostridium* | rs1434153 | G | A | 0.316 | -0.068 | 0.015 | 6.85E-06 |
| *Erysipelatoclostridium* | rs16936671 | C | T | 0.131 | -0.097 | 0.022 | 6.04E-06 |
| *Erysipelatoclostridium* | rs2901723 | C | A | 0.51 | 0.064 | 0.014 | 8.79E-06 |
| *Erysipelatoclostridium* | rs340991 | A | G | 0.299 | -0.074 | 0.016 | 3.75E-06 |
| *Erysipelatoclostridium* | rs45480394 | T | G | 0.374 | -0.069 | 0.015 | 7.66E-06 |
| *Erysipelatoclostridium* | rs4697572 | A | G | 0.259 | -0.081 | 0.016 | 7.59E-07 |
| *Erysipelatoclostridium* | rs58236560 | G | T | 0.101 | -0.111 | 0.023 | 2.16E-06 |
| *Erysipelatoclostridium* | rs61806970 | C | T | 0.061 | 0.143 | 0.032 | 9.09E-06 |
| *Erysipelatoclostridium* | rs622418 | A | G | 0.497 | -0.067 | 0.014 | 3.68E-06 |
| *Erysipelatoclostridium* | rs6474512 | A | C | 0.459 | 0.067 | 0.014 | 3.02E-06 |
| *Erysipelatoclostridium* | rs710230 | T | C | 0.074 | 0.143 | 0.028 | 6.33E-07 |
| *Erysipelatoclostridium* | rs7221249 | A | G | 0.526 | 0.084 | 0.014 | 4.31E-09 |
| *Erysipelatoclostridium* | rs9590927 | G | A | 0.483 | -0.065 | 0.014 | 6.39E-06 |
| *Erysipelotrichaceae UCG-003* | rs11666127 | A | G | 0.167 | -0.072 | 0.016 | 7.90E-06 |
| *Erysipelotrichaceae UCG-003* | rs11994308 | C | T | 0.063 | 0.115 | 0.024 | 1.33E-06 |
| *Erysipelotrichaceae UCG-003* | rs17798136 | G | A | 0.05 | 0.159 | 0.035 | 3.24E-06 |
| *Erysipelotrichaceae UCG-003* | rs28568391 | A | G | 0.43 | -0.058 | 0.012 | 6.42E-07 |
| *Erysipelotrichaceae UCG-003* | rs4758231 | G | T | 0.313 | -0.055 | 0.012 | 6.55E-06 |
| *Erysipelotrichaceae UCG-003* | rs59068084 | T | G | 0.41 | 0.056 | 0.012 | 3.12E-06 |
| *Erysipelotrichaceae UCG-003* | rs59104037 | A | G | 0.09 | -0.095 | 0.02 | 4.48E-06 |
| *Erysipelotrichaceae UCG-003* | rs62403464 | T | C | 0.165 | -0.073 | 0.016 | 3.44E-06 |
| *Erysipelotrichaceae UCG-003* | rs6875357 | C | T | 0.045 | 0.166 | 0.035 | 6.70E-06 |
| *Erysipelotrichaceae UCG-003* | rs73074432 | C | T | 0.154 | 0.072 | 0.016 | 9.99E-06 |
| *Erysipelotrichaceae UCG-003* | rs74988980 | G | A | 0.046 | -0.133 | 0.035 | 8.64E-06 |
| *Erysipelotrichaceae UCG-003* | rs75949021 | T | C | 0.053 | -0.17 | 0.037 | 3.58E-06 |
| *Erysipelotrichaceae UCG-003* | rs76502207 | T | C | 0.06 | 0.145 | 0.029 | 6.41E-07 |
| *Erysipelotrichaceae UCG-003* | rs8053479 | A | G | 0.122 | -0.084 | 0.019 | 5.83E-06 |
| *Escherichia Shigella* | rs112767262 | T | C | 0.19 | 0.073 | 0.016 | 8.21E-06 |
| *Escherichia Shigella* | rs113127095 | A | G | 0.063 | 0.151 | 0.032 | 3.33E-06 |
| *Escherichia Shigella* | rs113513883 | A | G | 0.06 | 0.172 | 0.038 | 5.28E-06 |
| *Escherichia Shigella* | rs1154904 | A | G | 0.463 | -0.061 | 0.013 | 3.04E-06 |
| *Escherichia Shigella* | rs118526 | C | A | 0.407 | -0.059 | 0.014 | 8.00E-06 |
| *Escherichia Shigella* | rs2798105 | A | G | 0.095 | -0.101 | 0.022 | 8.25E-06 |
| *Escherichia Shigella* | rs4731451 | G | A | 0.357 | -0.061 | 0.014 | 7.47E-06 |
| *Escherichia Shigella* | rs57024273 | T | C | 0.318 | 0.063 | 0.014 | 9.70E-06 |
| *Escherichia Shigella* | rs73208162 | A | G | 0.072 | -0.119 | 0.025 | 2.19E-06 |
| *Faecalibacterium* | rs10927394 | G | T | 0.031 | -0.232 | 0.051 | 7.02E-06 |
| *Faecalibacterium* | rs114946999 | C | T | 0.098 | -0.086 | 0.019 | 5.70E-06 |
| *Faecalibacterium* | rs11776390 | T | C | 0.113 | -0.078 | 0.017 | 6.40E-06 |
| *Faecalibacterium* | rs1271565 | C | T | 0.27 | -0.058 | 0.012 | 1.30E-06 |
| *Faecalibacterium* | rs12753492 | A | C | 0.161 | 0.064 | 0.015 | 8.80E-06 |
| *Faecalibacterium* | rs2835874 | T | C | 0.073 | -0.087 | 0.02 | 7.54E-06 |
| *Faecalibacterium* | rs6910935 | A | G | 0.059 | 0.135 | 0.028 | 1.38E-06 |
| *Faecalibacterium* | rs75499067 | C | T | 0.053 | 0.228 | 0.047 | 1.76E-06 |
| *Faecalibacterium* | rs79656633 | T | C | 0.053 | 0.146 | 0.032 | 8.14E-06 |
| *Faecalibacterium* | rs9536330 | T | C | 0.392 | -0.048 | 0.011 | 5.33E-06 |
| *Family XIII AD3011* | rs11126423 | C | T | 0.104 | 0.09 | 0.02 | 5.91E-06 |
| *Family XIII AD3011* | rs12812672 | T | C | 0.092 | -0.096 | 0.021 | 2.56E-06 |
| *Family XIII AD3011* | rs149302 | T | C | 0.212 | -0.065 | 0.014 | 7.48E-06 |
| *Family XIII AD3011* | rs16840310 | A | G | 0.359 | -0.061 | 0.012 | 6.75E-07 |
| *Family XIII AD3011* | rs17156849 | G | A | 0.073 | -0.113 | 0.025 | 4.19E-06 |
| *Family XIII AD3011* | rs62029761 | A | G | 0.068 | 0.129 | 0.028 | 3.89E-06 |
| *Family XIII AD3011* | rs62200412 | C | T | 0.165 | -0.08 | 0.016 | 5.80E-07 |
| *Family XIII AD3011* | rs72730932 | C | A | 0.157 | -0.09 | 0.018 | 6.89E-07 |
| *Family XIII AD3011* | rs739451 | C | T | 0.202 | 0.065 | 0.015 | 7.88E-06 |
| *Family XIII AD3011* | rs9837139 | A | G | 0.068 | 0.108 | 0.024 | 8.71E-06 |
| *Family XIII UCG001* | rs1426266 | T | C | 0.297 | -0.067 | 0.014 | 1.25E-06 |
| *Family XIII UCG001* | rs3842897 | G | A | 0.076 | -0.113 | 0.024 | 5.20E-06 |
| *Family XIII UCG001* | rs62414802 | C | T | 0.34 | -0.061 | 0.013 | 4.29E-06 |
| *Family XIII UCG001* | rs7119679 | G | A | 0.145 | -0.081 | 0.017 | 3.52E-06 |
| *Family XIII UCG001* | rs76463770 | A | G | 0.048 | 0.193 | 0.042 | 3.77E-06 |
| *Family XIII UCG001* | rs8076666 | A | G | 0.107 | 0.089 | 0.02 | 8.02E-06 |
| *Flavonifractor* | rs114873521 | C | T | 0.07 | -0.13 | 0.029 | 7.13E-06 |
| *Flavonifractor* | rs11811696 | T | C | 0.09 | -0.116 | 0.024 | 2.07E-06 |
| *Flavonifractor* | rs12030302 | A | G | 0.447 | -0.069 | 0.014 | 5.61E-07 |
| *Flavonifractor* | rs34066017 | A | G | 0.266 | 0.076 | 0.016 | 1.52E-06 |
| *Fusicatenibacter* | rs10439674 | A | G | 0.2 | -0.057 | 0.013 | 7.68E-06 |
| *Fusicatenibacter* | rs1864685 | A | C | 0.477 | -0.049 | 0.011 | 4.96E-06 |
| *Fusicatenibacter* | rs2025938 | G | A | 0.084 | -0.097 | 0.021 | 2.99E-06 |
| *Fusicatenibacter* | rs206581 | A | G | 0.228 | -0.057 | 0.013 | 8.96E-06 |
| *Fusicatenibacter* | rs2132128 | G | A | 0.131 | -0.077 | 0.016 | 1.08E-06 |
| *Fusicatenibacter* | rs4378146 | A | C | 0.256 | -0.062 | 0.013 | 7.20E-07 |
| *Fusicatenibacter* | rs62187631 | T | C | 0.154 | -0.071 | 0.016 | 4.55E-06 |
| *Fusicatenibacter* | rs6515626 | G | A | 0.042 | 0.142 | 0.031 | 7.29E-06 |
| *Fusicatenibacter* | rs704418 | T | C | 0.173 | 0.074 | 0.015 | 7.77E-07 |
| *Fusicatenibacter* | rs73103914 | A | G | 0.212 | -0.06 | 0.013 | 8.30E-06 |
| *Fusicatenibacter* | rs792108 | T | C | 0.355 | -0.051 | 0.011 | 8.50E-06 |
| *Fusicatenibacter* | rs8028026 | A | G | 0.113 | -0.079 | 0.018 | 8.06E-06 |
| *Fusicatenibacter* | rs8063430 | T | C | 0.057 | -0.104 | 0.022 | 4.93E-06 |
| *Fusicatenibacter* | rs9905659 | G | A | 0.184 | -0.062 | 0.014 | 7.31E-06 |
| *Gordonibacter* | rs13412653 | A | C | 0.352 | 0.108 | 0.024 | 8.61E-06 |
| *Gordonibacter* | rs16955299 | G | A | 0.075 | -0.196 | 0.043 | 6.37E-06 |
| *Gordonibacter* | rs322296 | G | A | 0.117 | 0.179 | 0.038 | 4.02E-06 |
| *Gordonibacter* | rs35042269 | C | A | 0.097 | -0.18 | 0.04 | 8.11E-06 |
| *Gordonibacter* | rs3765837 | T | G | 0.091 | -0.191 | 0.043 | 7.17E-06 |
| *Gordonibacter* | rs4596722 | A | G | 0.485 | 0.103 | 0.023 | 9.06E-06 |
| *Gordonibacter* | rs61934597 | C | T | 0.095 | -0.172 | 0.039 | 8.37E-06 |
| *Gordonibacter* | rs71545975 | A | G | 0.139 | -0.154 | 0.034 | 7.04E-06 |
| *Gordonibacter* | rs72714787 | C | A | 0.1 | 0.181 | 0.038 | 1.43E-06 |
| *Gordonibacter* | rs72939513 | A | G | 0.057 | -0.214 | 0.049 | 7.98E-06 |
| *Gordonibacter* | rs7294633 | C | T | 0.295 | 0.129 | 0.025 | 3.44E-07 |
| *Gordonibacter* | rs768830 | G | A | 0.134 | 0.15 | 0.033 | 7.76E-06 |
| *Haemophilus* | rs10781340 | G | A | 0.169 | 0.095 | 0.02 | 4.32E-06 |
| *Haemophilus* | rs111582866 | G | A | 0.106 | -0.124 | 0.026 | 1.27E-06 |
| *Haemophilus* | rs35509 | G | A | 0.094 | 0.128 | 0.027 | 2.01E-06 |
| *Haemophilus* | rs4822728 | T | C | 0.383 | 0.071 | 0.015 | 3.48E-06 |
| *Haemophilus* | rs76022354 | C | T | 0.046 | 0.245 | 0.051 | 1.83E-06 |
| *Haemophilus* | rs78909003 | T | C | 0.053 | -0.246 | 0.05 | 1.67E-06 |
| *Haemophilus* | rs9382510 | C | T | 0.233 | -0.094 | 0.017 | 7.12E-08 |
| *Haemophilus* | rs9895850 | T | C | 0.051 | -0.193 | 0.042 | 2.14E-06 |
| *Holdemanella* | rs12513188 | G | A | 0.196 | 0.09 | 0.02 | 4.65E-06 |
| *Holdemanella* | rs1926302 | G | A | 0.128 | -0.108 | 0.023 | 7.50E-06 |
| *Holdemanella* | rs34187114 | C | A | 0.156 | -0.105 | 0.023 | 5.13E-06 |
| *Holdemanella* | rs35228298 | G | A | 0.214 | 0.093 | 0.02 | 7.30E-06 |
| *Holdemanella* | rs4541991 | T | C | 0.221 | -0.093 | 0.019 | 2.10E-06 |
| *Holdemanella* | rs607782 | T | C | 0.374 | -0.085 | 0.017 | 7.19E-07 |
| *Holdemanella* | rs62113381 | T | C | 0.151 | -0.105 | 0.023 | 5.54E-06 |
| *Holdemanella* | rs73011279 | T | C | 0.224 | -0.096 | 0.02 | 1.36E-06 |
| *Holdemanella* | rs75764681 | T | C | 0.052 | -0.283 | 0.06 | 1.94E-06 |
| *Holdemanella* | rs8113760 | G | A | 0.351 | 0.079 | 0.017 | 4.62E-06 |
| *Holdemania* | rs10885477 | T | C | 0.063 | -0.135 | 0.03 | 8.60E-06 |
| *Holdemania* | rs11080063 | G | A | 0.494 | -0.067 | 0.015 | 6.67E-06 |
| *Holdemania* | rs111745969 | A | G | 0.099 | 0.121 | 0.027 | 3.71E-06 |
| *Holdemania* | rs113593397 | A | G | 0.088 | -0.129 | 0.028 | 9.36E-06 |
| *Holdemania* | rs116500994 | G | T | 0.071 | -0.138 | 0.029 | 2.34E-06 |
| *Holdemania* | rs12701617 | A | G | 0.429 | -0.066 | 0.015 | 9.52E-06 |
| *Holdemania* | rs1867876 | T | C | 0.283 | 0.084 | 0.016 | 2.74E-07 |
| *Holdemania* | rs4146507 | C | T | 0.22 | 0.079 | 0.018 | 7.23E-06 |
| *Holdemania* | rs6133067 | T | C | 0.245 | 0.091 | 0.018 | 5.17E-07 |
| *Holdemania* | rs73139538 | G | A | 0.062 | -0.149 | 0.033 | 7.77E-06 |
| *Holdemania* | rs80149660 | C | T | 0.06 | -0.233 | 0.052 | 6.04E-06 |
| *Holdemania* | rs9500080 | C | T | 0.222 | 0.093 | 0.018 | 4.09E-07 |
| *Holdemania* | rs9529719 | T | C | 0.306 | 0.074 | 0.016 | 5.97E-06 |
| *Holdemania* | rs967319 | T | C | 0.244 | 0.079 | 0.018 | 8.38E-06 |
| *Howardella* | rs10048062 | C | T | 0.126 | -0.147 | 0.034 | 8.59E-06 |
| *Howardella* | rs12452946 | A | G | 0.483 | -0.106 | 0.023 | 3.80E-06 |
| *Howardella* | rs1484873 | A | G | 0.054 | -0.228 | 0.046 | 2.56E-06 |
| *Howardella* | rs17167098 | G | A | 0.145 | -0.169 | 0.035 | 1.12E-06 |
| *Howardella* | rs2154047 | C | A | 0.084 | -0.193 | 0.042 | 9.97E-06 |
| *Howardella* | rs36081916 | T | C | 0.107 | -0.181 | 0.04 | 4.70E-06 |
| *Howardella* | rs3791893 | A | G | 0.119 | 0.147 | 0.034 | 9.50E-06 |
| *Howardella* | rs609430 | T | G | 0.34 | -0.112 | 0.024 | 3.34E-06 |
| *Howardella* | rs901099 | T | G | 0.309 | -0.127 | 0.025 | 6.53E-07 |
| *Hungatella* | rs10044993 | C | A | 0.147 | 0.14 | 0.032 | 8.07E-06 |
| *Hungatella* | rs13128780 | T | C | 0.147 | -0.15 | 0.031 | 1.75E-06 |
| *Hungatella* | rs13249325 | T | G | 0.382 | -0.1 | 0.023 | 9.69E-06 |
| *Hungatella* | rs17092615 | G | A | 0.133 | 0.152 | 0.034 | 7.38E-06 |
| *Hungatella* | rs72759041 | G | T | 0.237 | -0.126 | 0.028 | 3.86E-06 |
| *Intestinibacter* | rs16938435 | T | C | 0.075 | -0.112 | 0.024 | 1.80E-06 |
| *Intestinibacter* | rs2702387 | A | G | 0.339 | 0.061 | 0.013 | 4.26E-06 |
| *Intestinibacter* | rs4327025 | G | A | 0.217 | -0.081 | 0.015 | 1.64E-07 |
| *Intestinibacter* | rs447950 | A | G | 0.312 | 0.063 | 0.014 | 5.64E-06 |
| *Intestinibacter* | rs478972 | T | C | 0.049 | -0.143 | 0.03 | 1.82E-06 |
| *Intestinibacter* | rs6062862 | A | G | 0.121 | 0.092 | 0.02 | 6.68E-06 |
| *Intestinibacter* | rs62430350 | T | C | 0.052 | 0.151 | 0.035 | 6.84E-06 |
| *Intestinibacter* | rs6875660 | C | T | 0.111 | 0.089 | 0.019 | 3.06E-06 |
| *Intestinibacter* | rs9348442 | C | T | 0.076 | 0.099 | 0.022 | 6.26E-06 |
| *Intestinimonas* | rs10262702 | T | C | 0.126 | 0.092 | 0.019 | 2.06E-06 |
| *Intestinimonas* | rs11258178 | A | G | 0.373 | 0.066 | 0.013 | 6.98E-07 |
| *Intestinimonas* | rs12226153 | A | G | 0.058 | -0.151 | 0.031 | 5.12E-07 |
| *Intestinimonas* | rs17067892 | C | T | 0.071 | 0.107 | 0.025 | 6.38E-06 |
| *Intestinimonas* | rs1859797 | G | A | 0.471 | 0.06 | 0.013 | 4.12E-06 |
| *Intestinimonas* | rs2276760 | A | G | 0.216 | -0.069 | 0.015 | 7.84E-06 |
| *Intestinimonas* | rs2731794 | C | T | 0.069 | 0.121 | 0.026 | 1.92E-06 |
| *Intestinimonas* | rs4113676 | A | C | 0.052 | -0.219 | 0.049 | 7.42E-06 |
| *Intestinimonas* | rs4784055 | T | C | 0.041 | -0.175 | 0.039 | 8.72E-07 |
| *Intestinimonas* | rs62240188 | G | A | 0.08 | 0.13 | 0.027 | 2.20E-06 |
| *Intestinimonas* | rs62427239 | C | A | 0.055 | 0.163 | 0.037 | 9.41E-06 |
| *Intestinimonas* | rs6934519 | C | T | 0.254 | 0.069 | 0.015 | 8.57E-06 |
| *Intestinimonas* | rs716604 | A | G | 0.18 | 0.082 | 0.017 | 8.57E-07 |
| *Intestinimonas* | rs7170984 | T | C | 0.31 | -0.066 | 0.014 | 2.98E-06 |
| *Intestinimonas* | rs72982915 | C | T | 0.048 | 0.183 | 0.04 | 4.91E-06 |
| *Intestinimonas* | rs9823439 | T | C | 0.469 | -0.058 | 0.013 | 9.86E-06 |
| *Lachnoclostridium* | rs1031599 | G | T | 0.081 | -0.079 | 0.018 | 6.31E-06 |
| *Lachnoclostridium* | rs12566975 | T | C | 0.482 | -0.047 | 0.011 | 9.57E-06 |
| *Lachnoclostridium* | rs1528479 | G | A | 0.338 | -0.05 | 0.011 | 9.64E-06 |
| *Lachnoclostridium* | rs1997204 | T | C | 0.057 | -0.108 | 0.024 | 5.97E-06 |
| *Lachnoclostridium* | rs2385421 | A | G | 0.077 | 0.075 | 0.018 | 7.14E-06 |
| *Lachnoclostridium* | rs615997 | T | C | 0.478 | 0.051 | 0.011 | 2.03E-06 |
| *Lachnoclostridium* | rs62285313 | A | G | 0.107 | 0.086 | 0.018 | 1.58E-06 |
| *Lachnoclostridium* | rs72829893 | G | T | 0.053 | 0.117 | 0.027 | 5.58E-06 |
| *Lachnoclostridium* | rs78068103 | A | G | 0.072 | 0.089 | 0.019 | 3.67E-06 |
| *Lachnoclostridium* | rs789029 | C | T | 0.171 | -0.064 | 0.014 | 3.75E-06 |
| *Lachnospira* | rs13157098 | A | G | 0.154 | -0.077 | 0.016 | 5.99E-07 |
| *Lachnospira* | rs159484 | G | A | 0.092 | 0.079 | 0.018 | 6.68E-06 |
| *Lachnospira* | rs2520509 | A | G | 0.332 | 0.052 | 0.012 | 7.42E-06 |
| *Lachnospira* | rs4686798 | T | C | 0.372 | 0.053 | 0.011 | 2.74E-06 |
| *Lachnospira* | rs4923324 | G | A | 0.236 | -0.062 | 0.013 | 2.44E-06 |
| *Lachnospira* | rs56791201 | T | C | 0.482 | 0.052 | 0.011 | 2.93E-06 |
| *Lachnospiraceae FCS020* | rs10093861 | G | A | 0.492 | -0.057 | 0.012 | 3.06E-06 |
| *Lachnospiraceae FCS020* | rs1254846 | G | A | 0.075 | 0.106 | 0.023 | 5.60E-06 |
| *Lachnospiraceae FCS020* | rs1363769 | T | C | 0.052 | -0.201 | 0.045 | 1.58E-06 |
| *Lachnospiraceae FCS020* | rs2322265 | C | T | 0.246 | -0.067 | 0.014 | 5.21E-06 |
| *Lachnospiraceae FCS020* | rs2862811 | T | C | 0.446 | 0.056 | 0.012 | 3.92E-06 |
| *Lachnospiraceae FCS020* | rs4452603 | T | G | 0.265 | 0.06 | 0.014 | 8.98E-06 |
| *Lachnospiraceae FCS020* | rs7249113 | G | A | 0.288 | 0.068 | 0.013 | 3.72E-07 |
| *Lachnospiraceae FCS020* | rs72793667 | A | G | 0.082 | -0.117 | 0.025 | 1.63E-06 |
| *Lachnospiraceae FCS020* | rs9308097 | A | G | 0.412 | 0.055 | 0.012 | 7.47E-06 |
| *Lachnospiraceae FCS020* | rs9788306 | C | T | 0.299 | -0.063 | 0.013 | 1.39E-06 |
| *Lachnospiraceae NC2004* | rs117467633 | T | C | 0.072 | -0.17 | 0.038 | 9.13E-06 |
| *Lachnospiraceae NC2004* | rs12127733 | G | A | 0.173 | 0.115 | 0.025 | 3.11E-06 |
| *Lachnospiraceae NC2004* | rs12208226 | C | A | 0.082 | -0.155 | 0.034 | 9.75E-06 |
| *Lachnospiraceae NC2004* | rs12863463 | G | A | 0.076 | -0.156 | 0.035 | 6.04E-06 |
| *Lachnospiraceae NC2004* | rs1928659 | T | C | 0.249 | 0.103 | 0.023 | 6.17E-06 |
| *Lachnospiraceae NC2004* | rs1929743 | T | C | 0.342 | 0.084 | 0.019 | 9.06E-06 |
| *Lachnospiraceae NC2004* | rs3756315 | A | G | 0.324 | -0.088 | 0.019 | 3.33E-06 |
| *Lachnospiraceae NC2004* | rs6116753 | G | A | 0.257 | 0.099 | 0.021 | 2.92E-06 |
| *Lachnospiraceae ND3007* | rs2861203 | G | A | 0.295 | 0.057 | 0.013 | 7.37E-06 |
| *Lachnospiraceae ND3007* | rs72776675 | T | C | 0.184 | -0.065 | 0.015 | 8.72E-06 |
| *Lachnospiraceae ND3007* | rs9932954 | A | G | 0.454 | -0.056 | 0.012 | 1.25E-06 |
| *Lachnospiraceae NK4A136* | rs10952110 | G | T | 0.455 | 0.049 | 0.011 | 9.08E-06 |
| *Lachnospiraceae NK4A136* | rs11263806 | A | G | 0.32 | -0.052 | 0.012 | 5.07E-06 |
| *Lachnospiraceae NK4A136* | rs12611395 | A | G | 0.098 | -0.09 | 0.02 | 5.83E-06 |
| *Lachnospiraceae NK4A136* | rs160061 | A | G | 0.499 | 0.051 | 0.011 | 2.12E-06 |
| *Lachnospiraceae NK4A136* | rs28540839 | A | C | 0.488 | 0.051 | 0.011 | 9.34E-06 |
| *Lachnospiraceae NK4A136* | rs2880566 | T | C | 0.202 | 0.06 | 0.013 | 5.61E-06 |
| *Lachnospiraceae NK4A136* | rs4955932 | T | C | 0.405 | -0.049 | 0.011 | 7.05E-06 |
| *Lachnospiraceae NK4A136* | rs59805249 | T | C | 0.058 | 0.094 | 0.021 | 9.45E-06 |
| *Lachnospiraceae NK4A136* | rs68104925 | T | C | 0.354 | -0.055 | 0.012 | 2.37E-06 |
| *Lachnospiraceae NK4A136* | rs73044693 | A | G | 0.086 | -0.108 | 0.023 | 3.57E-06 |
| *Lachnospiraceae NK4A136* | rs7616165 | G | T | 0.048 | -0.231 | 0.048 | 2.77E-06 |
| *Lachnospiraceae NK4A136* | rs76193507 | A | G | 0.045 | -0.23 | 0.05 | 2.93E-06 |
| *Lachnospiraceae NK4A136* | rs7832116 | A | G | 0.145 | -0.071 | 0.015 | 3.57E-06 |
| *Lachnospiraceae NK4A136* | rs954878 | A | G | 0.45 | -0.052 | 0.011 | 1.78E-06 |
| *Lachnospiraceae UCG001* | rs12131224 | C | T | 0.094 | 0.117 | 0.026 | 7.40E-06 |
| *Lachnospiraceae UCG001* | rs2050911 | G | A | 0.295 | 0.075 | 0.015 | 1.11E-06 |
| *Lachnospiraceae UCG001* | rs2371284 | T | C | 0.222 | -0.076 | 0.017 | 7.56E-06 |
| *Lachnospiraceae UCG001* | rs437876 | T | C | 0.376 | 0.078 | 0.014 | 7.17E-08 |
| *Lachnospiraceae UCG001* | rs4981345 | T | C | 0.336 | -0.068 | 0.015 | 6.09E-06 |
| *Lachnospiraceae UCG001* | rs573933 | T | C | 0.091 | -0.108 | 0.023 | 3.11E-06 |
| *Lachnospiraceae UCG001* | rs62496417 | T | G | 0.236 | -0.075 | 0.017 | 5.88E-06 |
| *Lachnospiraceae UCG001* | rs7341608 | T | C | 0.184 | -0.078 | 0.018 | 9.48E-06 |
| *Lachnospiraceae UCG001* | rs74034332 | G | A | 0.049 | 0.168 | 0.038 | 3.33E-06 |
| *Lachnospiraceae UCG001* | rs78848836 | A | G | 0.092 | -0.119 | 0.026 | 3.38E-06 |
| *Lachnospiraceae UCG001* | rs8104225 | A | G | 0.153 | 0.089 | 0.02 | 8.04E-06 |
| *Lachnospiraceae UCG001* | rs9403580 | C | T | 0.102 | 0.108 | 0.023 | 3.47E-06 |
| *Lachnospiraceae UCG001* | rs985416 | C | T | 0.194 | 0.097 | 0.018 | 1.46E-07 |
| *Lachnospiraceae UCG004* | rs11128180 | A | G | 0.235 | 0.065 | 0.014 | 4.52E-06 |
| *Lachnospiraceae UCG004* | rs12072562 | T | C | 0.059 | 0.133 | 0.03 | 7.07E-06 |
| *Lachnospiraceae UCG004* | rs12673420 | G | A | 0.424 | 0.055 | 0.012 | 2.98E-06 |
| *Lachnospiraceae UCG004* | rs12747809 | G | A | 0.317 | -0.062 | 0.013 | 8.65E-07 |
| *Lachnospiraceae UCG004* | rs12894272 | A | G | 0.326 | 0.058 | 0.013 | 4.34E-06 |
| *Lachnospiraceae UCG004* | rs233486 | A | G | 0.137 | -0.08 | 0.018 | 6.28E-06 |
| *Lachnospiraceae UCG004* | rs2726805 | A | G | 0.391 | 0.055 | 0.012 | 6.30E-06 |
| *Lachnospiraceae UCG004* | rs2882478 | G | A | 0.447 | -0.058 | 0.012 | 1.21E-06 |
| *Lachnospiraceae UCG004* | rs35182105 | A | G | 0.071 | -0.11 | 0.024 | 4.87E-06 |
| *Lachnospiraceae UCG004* | rs7629954 | A | G | 0.071 | 0.108 | 0.024 | 5.77E-06 |
| *Lachnospiraceae UCG008* | rs10741777 | T | C | 0.238 | -0.097 | 0.019 | 7.69E-07 |
| *Lachnospiraceae UCG008* | rs10793103 | C | T | 0.312 | 0.097 | 0.018 | 9.35E-08 |
| *Lachnospiraceae UCG008* | rs10801803 | G | A | 0.131 | -0.117 | 0.024 | 1.40E-06 |
| *Lachnospiraceae UCG008* | rs13024781 | T | C | 0.458 | -0.08 | 0.017 | 2.29E-06 |
| *Lachnospiraceae UCG008* | rs57254474 | G | A | 0.239 | 0.089 | 0.02 | 6.92E-06 |
| *Lachnospiraceae UCG008* | rs61944774 | A | G | 0.062 | 0.18 | 0.039 | 6.34E-06 |
| *Lachnospiraceae UCG008* | rs62277846 | C | T | 0.2 | 0.102 | 0.021 | 1.59E-06 |
| *Lachnospiraceae UCG008* | rs67078837 | T | C | 0.48 | -0.085 | 0.017 | 7.68E-07 |
| *Lachnospiraceae UCG008* | rs75356640 | G | A | 0.087 | 0.137 | 0.03 | 9.83E-06 |
| *Lachnospiraceae UCG008* | rs955844 | A | C | 0.166 | 0.112 | 0.023 | 1.81E-06 |
| *Lachnospiraceae UCG010* | rs10414815 | T | C | 0.12 | 0.105 | 0.023 | 4.24E-06 |
| *Lachnospiraceae UCG010* | rs11192447 | A | G | 0.062 | 0.127 | 0.024 | 4.69E-07 |
| *Lachnospiraceae UCG010* | rs12346653 | C | T | 0.243 | 0.066 | 0.014 | 2.70E-06 |
| *Lachnospiraceae UCG010* | rs17730011 | G | A | 0.18 | -0.07 | 0.016 | 7.85E-06 |
| *Lachnospiraceae UCG010* | rs2833528 | C | T | 0.37 | -0.056 | 0.013 | 9.92E-06 |
| *Lachnospiraceae UCG010* | rs336138 | G | T | 0.164 | 0.078 | 0.017 | 7.48E-06 |
| *Lachnospiraceae UCG010* | rs4576377 | A | C | 0.391 | -0.057 | 0.013 | 7.63E-06 |
| *Lachnospiraceae UCG010* | rs72894957 | G | A | 0.046 | 0.222 | 0.049 | 5.68E-06 |
| *Lachnospiraceae UCG010* | rs74315802 | G | T | 0.149 | 0.087 | 0.018 | 3.19E-06 |
| *Lachnospiraceae UCG010* | rs9981767 | A | C | 0.321 | 0.066 | 0.013 | 9.96E-07 |
| *Lactobacillus* | rs11674854 | C | T | 0.416 | -0.085 | 0.018 | 1.59E-06 |
| *Lactobacillus* | rs12693845 | C | T | 0.391 | -0.081 | 0.018 | 8.96E-06 |
| *Lactobacillus* | rs16861661 | G | A | 0.07 | -0.183 | 0.038 | 1.28E-06 |
| *Lactobacillus* | rs62314653 | C | A | 0.053 | 0.188 | 0.039 | 2.24E-06 |
| *Lactobacillus* | rs7399658 | G | A | 0.21 | -0.107 | 0.022 | 3.12E-06 |
| *Lactobacillus* | rs75127669 | C | A | 0.081 | 0.14 | 0.031 | 6.83E-06 |
| *Lactobacillus* | rs768253 | T | G | 0.391 | -0.079 | 0.017 | 4.25E-06 |
| *Lactobacillus* | rs77478751 | A | G | 0.065 | -0.22 | 0.048 | 7.33E-06 |
| *Lactobacillus* | rs921925 | A | C | 0.209 | 0.099 | 0.02 | 9.72E-07 |
| *Lactococcus* | rs10417872 | T | G | 0.275 | 0.118 | 0.025 | 1.29E-06 |
| *Lactococcus* | rs123059 | T | C | 0.229 | -0.137 | 0.027 | 1.27E-06 |
| *Lactococcus* | rs12621813 | G | A | 0.301 | 0.108 | 0.024 | 6.61E-06 |
| *Lactococcus* | rs17168302 | G | A | 0.068 | 0.192 | 0.042 | 6.29E-06 |
| *Lactococcus* | rs2293361 | C | T | 0.061 | -0.199 | 0.043 | 1.40E-06 |
| *Lactococcus* | rs4766997 | C | T | 0.31 | 0.115 | 0.024 | 2.06E-06 |
| *Lactococcus* | rs55910161 | C | T | 0.161 | 0.146 | 0.031 | 2.36E-06 |
| *Lactococcus* | rs6674304 | C | T | 0.07 | 0.201 | 0.044 | 6.18E-06 |
| *Lactococcus* | rs7992246 | T | C | 0.365 | 0.104 | 0.023 | 4.45E-06 |
| *Marvinbryantia* | rs11620597 | T | C | 0.066 | 0.119 | 0.027 | 7.80E-06 |
| *Marvinbryantia* | rs1187983 | C | T | 0.137 | -0.094 | 0.019 | 2.02E-06 |
| *Marvinbryantia* | rs146541147 | G | A | 0.066 | 0.119 | 0.027 | 6.86E-06 |
| *Marvinbryantia* | rs2724813 | A | G | 0.177 | -0.084 | 0.017 | 6.28E-07 |
| *Marvinbryantia* | rs2842896 | C | T | 0.504 | -0.065 | 0.013 | 7.25E-07 |
| *Marvinbryantia* | rs2863363 | A | G | 0.361 | 0.063 | 0.014 | 3.11E-06 |
| *Marvinbryantia* | rs3125832 | A | C | 0.248 | 0.068 | 0.015 | 5.03E-06 |
| *Marvinbryantia* | rs61884471 | G | A | 0.076 | 0.124 | 0.025 | 1.01E-06 |
| *Marvinbryantia* | rs72948274 | A | C | 0.058 | -0.126 | 0.027 | 3.26E-06 |
| *Marvinbryantia* | rs8006832 | G | T | 0.108 | -0.095 | 0.022 | 6.58E-06 |
| *Methanobrevibacter* | rs10202904 | T | G | 0.413 | -0.113 | 0.024 | 3.09E-06 |
| *Methanobrevibacter* | rs1334944 | T | C | 0.329 | 0.115 | 0.026 | 7.61E-06 |
| *Methanobrevibacter* | rs4802933 | A | G | 0.162 | -0.136 | 0.031 | 9.74E-06 |
| *Methanobrevibacter* | rs6776814 | T | C | 0.095 | -0.189 | 0.042 | 8.05E-06 |
| *Methanobrevibacter* | rs76029318 | T | C | 0.073 | 0.223 | 0.045 | 1.08E-06 |
| *Methanobrevibacter* | rs894996 | C | A | 0.076 | 0.214 | 0.046 | 3.82E-06 |
| *Odoribacter* | rs10093869 | A | G | 0.34 | -0.058 | 0.013 | 3.67E-06 |
| *Odoribacter* | rs10423795 | C | T | 0.409 | 0.055 | 0.012 | 6.58E-06 |
| *Odoribacter* | rs28417404 | A | G | 0.16 | -0.073 | 0.016 | 3.68E-06 |
| *Odoribacter* | rs4793970 | A | G | 0.291 | -0.058 | 0.013 | 6.03E-06 |
| *Odoribacter* | rs6856150 | G | A | 0.098 | 0.088 | 0.019 | 6.06E-06 |
| *Odoribacter* | rs74553962 | T | G | 0.068 | 0.121 | 0.026 | 9.49E-06 |
| *Odoribacter* | rs77779484 | G | A | 0.056 | -0.133 | 0.027 | 6.56E-07 |
| *Olsenella* | rs1035588 | A | G | 0.367 | -0.108 | 0.024 | 4.86E-06 |
| *Olsenella* | rs17148768 | G | A | 0.191 | 0.14 | 0.03 | 2.20E-06 |
| *Olsenella* | rs35225860 | A | G | 0.064 | -0.224 | 0.048 | 3.87E-06 |
| *Olsenella* | rs6046522 | C | T | 0.252 | 0.123 | 0.027 | 4.48E-06 |
| *Olsenella* | rs61090148 | A | G | 0.46 | -0.105 | 0.023 | 6.44E-06 |
| *Olsenella* | rs72691585 | C | A | 0.054 | -0.249 | 0.052 | 2.95E-06 |
| *Olsenella* | rs7540303 | C | T | 0.398 | 0.108 | 0.024 | 5.32E-06 |
| *Olsenella* | rs9460691 | C | A | 0.232 | 0.12 | 0.027 | 7.28E-06 |
| *Oscillibacter* | rs11627628 | T | C | 0.078 | 0.144 | 0.029 | 1.01E-06 |
| *Oscillibacter* | rs12649930 | T | G | 0.085 | 0.122 | 0.026 | 4.09E-06 |
| *Oscillibacter* | rs133832 | A | C | 0.326 | -0.08 | 0.016 | 1.15E-06 |
| *Oscillibacter* | rs16866406 | A | G | 0.137 | 0.099 | 0.021 | 3.08E-06 |
| *Oscillibacter* | rs16934185 | A | G | 0.09 | -0.13 | 0.028 | 4.38E-06 |
| *Oscillibacter* | rs234108 | A | G | 0.477 | 0.075 | 0.015 | 9.16E-07 |
| *Oscillibacter* | rs36095275 | C | T | 0.374 | -0.075 | 0.016 | 1.40E-06 |
| *Oscillibacter* | rs4506202 | A | G | 0.535 | -0.071 | 0.015 | 3.21E-06 |
| *Oscillibacter* | rs61883564 | A | G | 0.135 | -0.101 | 0.022 | 3.39E-06 |
| *Oscillibacter* | rs62206502 | C | A | 0.455 | -0.068 | 0.015 | 6.60E-06 |
| *Oscillibacter* | rs75453768 | G | T | 0.094 | 0.122 | 0.027 | 5.35E-06 |
| *Oscillibacter* | rs761240 | T | G | 0.075 | -0.177 | 0.039 | 2.04E-06 |
| *Oscillospira* | rs12206468 | G | A | 0.078 | -0.133 | 0.027 | 1.04E-06 |
| *Oscillospira* | rs1954532 | T | C | 0.206 | -0.083 | 0.018 | 2.27E-06 |
| *Oscillospira* | rs28889936 | A | C | 0.073 | 0.114 | 0.025 | 3.37E-06 |
| *Oscillospira* | rs62422654 | C | T | 0.16 | 0.09 | 0.02 | 6.47E-06 |
| *Oscillospira* | rs72866977 | A | C | 0.079 | -0.131 | 0.028 | 5.63E-06 |
| *Oscillospira* | rs751183 | T | C | 0.202 | -0.077 | 0.017 | 6.85E-06 |
| *Oscillospira* | rs8076323 | A | G | 0.258 | 0.072 | 0.016 | 5.61E-06 |
| *Oxalobacter* | rs10464997 | G | A | 0.135 | 0.138 | 0.029 | 3.30E-06 |
| *Oxalobacter* | rs11108500 | A | G | 0.068 | -0.199 | 0.043 | 3.74E-06 |
| *Oxalobacter* | rs111966731 | T | C | 0.062 | 0.213 | 0.047 | 7.30E-06 |
| *Oxalobacter* | rs12002250 | A | C | 0.056 | 0.217 | 0.047 | 1.42E-06 |
| *Oxalobacter* | rs1569853 | T | C | 0.153 | -0.138 | 0.03 | 3.65E-06 |
| *Oxalobacter* | rs36057338 | G | T | 0.069 | 0.208 | 0.042 | 8.80E-07 |
| *Oxalobacter* | rs3862635 | C | T | 0.067 | -0.172 | 0.039 | 9.19E-06 |
| *Oxalobacter* | rs6993398 | G | A | 0.142 | 0.127 | 0.028 | 7.13E-06 |
| *Oxalobacter* | rs736744 | C | T | 0.396 | 0.118 | 0.021 | 2.57E-08 |
| *Parabacteroides* | rs114567323 | T | C | 0.048 | 0.186 | 0.041 | 5.65E-06 |
| *Parabacteroides* | rs115602804 | G | A | 0.057 | 0.103 | 0.022 | 1.93E-06 |
| *Parabacteroides* | rs4236095 | G | A | 0.141 | 0.076 | 0.016 | 1.93E-06 |
| *Parabacteroides* | rs60884758 | C | T | 0.174 | -0.07 | 0.014 | 5.71E-07 |
| *Parabacteroides* | rs6657302 | T | C | 0.058 | -0.105 | 0.023 | 9.76E-06 |
| *Parabacteroides* | rs7298818 | C | T | 0.086 | 0.089 | 0.02 | 8.54E-06 |
| *Paraprevotella* | rs10842464 | T | C | 0.425 | -0.076 | 0.017 | 6.60E-06 |
| *Paraprevotella* | rs140997932 | T | C | 0.071 | -0.162 | 0.035 | 2.11E-06 |
| *Paraprevotella* | rs145020347 | A | G | 0.118 | -0.125 | 0.026 | 4.03E-06 |
| *Paraprevotella* | rs17109926 | A | G | 0.188 | -0.099 | 0.022 | 6.75E-06 |
| *Paraprevotella* | rs17785622 | A | G | 0.062 | 0.248 | 0.052 | 1.93E-06 |
| *Paraprevotella* | rs2081023 | A | G | 0.121 | -0.123 | 0.024 | 2.64E-07 |
| *Paraprevotella* | rs3008582 | T | C | 0.152 | 0.106 | 0.023 | 4.36E-06 |
| *Paraprevotella* | rs3801748 | G | A | 0.37 | 0.078 | 0.017 | 5.20E-06 |
| *Paraprevotella* | rs4756632 | G | T | 0.077 | -0.139 | 0.029 | 3.82E-06 |
| *Paraprevotella* | rs4767113 | C | T | 0.3 | 0.088 | 0.018 | 2.14E-06 |
| *Paraprevotella* | rs7240324 | T | G | 0.185 | -0.102 | 0.023 | 5.96E-06 |
| *Paraprevotella* | rs9602779 | A | C | 0.189 | -0.107 | 0.022 | 6.93E-07 |
| *Parasutterella* | rs10899911 | A | G | 0.281 | -0.072 | 0.015 | 1.15E-06 |
| *Parasutterella* | rs11715853 | G | A | 0.283 | -0.066 | 0.015 | 6.23E-06 |
| *Parasutterella* | rs2090816 | A | C | 0.169 | 0.084 | 0.018 | 2.90E-06 |
| *Parasutterella* | rs2387977 | T | C | 0.414 | -0.068 | 0.013 | 5.38E-07 |
| *Parasutterella* | rs35055552 | T | C | 0.103 | 0.11 | 0.024 | 3.35E-06 |
| *Parasutterella* | rs55877868 | A | C | 0.105 | -0.104 | 0.023 | 2.87E-06 |
| *Parasutterella* | rs6809952 | G | A | 0.304 | -0.068 | 0.015 | 8.13E-06 |
| *Parasutterella* | rs6828768 | C | T | 0.496 | 0.064 | 0.013 | 1.78E-06 |
| *Parasutterella* | rs7303158 | C | T | 0.438 | 0.065 | 0.013 | 1.33E-06 |
| *Parasutterella* | rs7311004 | T | C | 0.401 | -0.062 | 0.014 | 5.92E-06 |
| *Parasutterella* | rs7572229 | G | A | 0.484 | 0.066 | 0.013 | 6.32E-07 |
| *Parasutterella* | rs78383039 | T | C | 0.062 | -0.146 | 0.03 | 1.57E-06 |
| *Parasutterella* | rs823424 | G | A | 0.222 | -0.071 | 0.016 | 4.95E-06 |
| *Peptococcus* | rs10031059 | T | C | 0.229 | -0.121 | 0.023 | 1.24E-07 |
| *Peptococcus* | rs11001941 | G | A | 0.067 | -0.196 | 0.039 | 1.33E-06 |
| *Peptococcus* | rs12069354 | C | T | 0.064 | 0.168 | 0.038 | 9.28E-06 |
| *Peptococcus* | rs2054133 | G | A | 0.513 | 0.09 | 0.019 | 2.14E-06 |
| *Peptococcus* | rs36121075 | A | G | 0.111 | -0.141 | 0.031 | 6.99E-06 |
| *Peptococcus* | rs413827 | G | A | 0.206 | 0.11 | 0.024 | 3.30E-06 |
| *Peptococcus* | rs62424012 | G | A | 0.114 | 0.137 | 0.029 | 1.15E-06 |
| *Peptococcus* | rs7033353 | T | G | 0.401 | 0.09 | 0.019 | 2.22E-06 |
| *Peptococcus* | rs72850165 | T | C | 0.11 | -0.134 | 0.03 | 5.74E-06 |
| *Peptococcus* | rs74592222 | G | A | 0.108 | 0.138 | 0.03 | 8.55E-06 |
| *Peptococcus* | rs77681628 | C | T | 0.065 | 0.2 | 0.039 | 2.69E-07 |
| *Phascolarctobacterium* | rs11929846 | T | C | 0.21 | -0.07 | 0.016 | 8.88E-06 |
| *Phascolarctobacterium* | rs12618201 | A | G | 0.365 | 0.064 | 0.014 | 3.38E-06 |
| *Phascolarctobacterium* | rs1264476 | T | G | 0.21 | 0.077 | 0.017 | 4.30E-06 |
| *Phascolarctobacterium* | rs56069061 | G | A | 0.107 | -0.111 | 0.023 | 1.87E-06 |
| *Phascolarctobacterium* | rs56157888 | A | C | 0.161 | 0.095 | 0.019 | 1.09E-06 |
| *Phascolarctobacterium* | rs74540770 | G | A | 0.089 | -0.121 | 0.026 | 3.60E-06 |
| *Phascolarctobacterium* | rs74847270 | A | G | 0.106 | -0.105 | 0.023 | 5.73E-06 |
| *Phascolarctobacterium* | rs75882962 | T | C | 0.147 | 0.097 | 0.019 | 3.19E-07 |
| *Phascolarctobacterium* | rs7982713 | G | A | 0.229 | 0.073 | 0.016 | 9.72E-06 |
| *Prevotella7* | rs118038478 | A | G | 0.079 | 0.206 | 0.047 | 7.85E-06 |
| *Prevotella7* | rs12124567 | A | G | 0.287 | -0.121 | 0.028 | 9.49E-06 |
| *Prevotella7* | rs12195431 | T | C | 0.075 | 0.197 | 0.044 | 8.73E-06 |
| *Prevotella7* | rs2240542 | C | T | 0.281 | 0.121 | 0.026 | 4.84E-06 |
| *Prevotella7* | rs2918132 | C | T | 0.361 | -0.115 | 0.025 | 6.42E-06 |
| *Prevotella7* | rs430270 | A | C | 0.2 | 0.139 | 0.03 | 2.87E-06 |
| *Prevotella7* | rs57404562 | C | A | 0.19 | 0.155 | 0.032 | 6.22E-07 |
| *Prevotella7* | rs79263163 | A | C | 0.169 | -0.144 | 0.032 | 7.51E-06 |
| *Prevotella7* | rs9426434 | T | C | 0.268 | -0.124 | 0.028 | 9.72E-06 |
| *Prevotella7* | rs9608249 | A | G | 0.148 | -0.158 | 0.034 | 2.07E-06 |
| *Prevotella7* | rs9959718 | G | A | 0.257 | 0.133 | 0.028 | 1.90E-06 |
| *Prevotella9* | rs111509883 | T | C | 0.067 | 0.171 | 0.035 | 1.24E-06 |
| *Prevotella9* | rs11685699 | C | T | 0.078 | -0.141 | 0.03 | 2.03E-06 |
| *Prevotella9* | rs117271932 | A | G | 0.055 | 0.208 | 0.044 | 2.82E-06 |
| *Prevotella9* | rs12648235 | T | C | 0.146 | 0.079 | 0.018 | 7.39E-06 |
| *Prevotella9* | rs1304512 | G | A | 0.241 | 0.076 | 0.017 | 5.29E-06 |
| *Prevotella9* | rs2104588 | T | C | 0.107 | 0.106 | 0.024 | 8.13E-06 |
| *Prevotella9* | rs2495052 | A | G | 0.172 | 0.084 | 0.019 | 8.97E-06 |
| *Prevotella9* | rs2683313 | A | G | 0.326 | -0.072 | 0.015 | 1.69E-06 |
| *Prevotella9* | rs4968431 | G | T | 0.383 | 0.064 | 0.014 | 8.58E-06 |
| *Prevotella9* | rs7237249 | C | T | 0.185 | -0.082 | 0.018 | 8.93E-06 |
| *Prevotella9* | rs72815774 | T | C | 0.078 | -0.176 | 0.039 | 8.78E-06 |
| *Prevotella9* | rs746764 | T | C | 0.181 | -0.092 | 0.019 | 2.04E-06 |
| *Prevotella9* | rs7976209 | T | C | 0.146 | -0.087 | 0.02 | 7.28E-06 |
| *Prevotella9* | rs9613013 | G | A | 0.133 | 0.092 | 0.02 | 6.10E-06 |
| *Rikenellaceae RC9 gut group* | rs12501673 | A | G | 0.35 | 0.116 | 0.026 | 6.29E-06 |
| *Rikenellaceae RC9 gut group* | rs17032291 | T | C | 0.142 | -0.17 | 0.037 | 6.61E-06 |
| *Rikenellaceae RC9 gut group* | rs17582787 | A | G | 0.146 | -0.158 | 0.034 | 3.55E-06 |
| *Rikenellaceae RC9 gut group* | rs2900503 | G | T | 0.16 | -0.172 | 0.033 | 1.55E-07 |
| *Rikenellaceae RC9 gut group* | rs2998141 | T | C | 0.206 | -0.136 | 0.029 | 4.42E-06 |
| *Rikenellaceae RC9 gut group* | rs4270579 | G | A | 0.279 | -0.118 | 0.027 | 5.46E-06 |
| *Rikenellaceae RC9 gut group* | rs4717843 | G | T | 0.349 | -0.119 | 0.026 | 4.72E-06 |
| *Rikenellaceae RC9 gut group* | rs7712231 | A | G | 0.147 | 0.156 | 0.035 | 7.97E-06 |
| *Rikenellaceae RC9 gut group* | rs80309088 | G | A | 0.121 | 0.174 | 0.038 | 4.56E-06 |
| *Rikenellaceae RC9 gut group* | rs9887954 | G | A | 0.418 | -0.115 | 0.025 | 4.81E-06 |
| *Romboutsia* | rs10279978 | A | G | 0.313 | -0.062 | 0.013 | 1.17E-06 |
| *Romboutsia* | rs11221428 | T | C | 0.173 | -0.073 | 0.016 | 6.49E-06 |
| *Romboutsia* | rs16843578 | C | T | 0.09 | -0.088 | 0.02 | 5.08E-06 |
| *Romboutsia* | rs28603357 | T | C | 0.046 | -0.215 | 0.047 | 8.52E-06 |
| *Romboutsia* | rs34302036 | A | G | 0.419 | 0.055 | 0.012 | 5.88E-06 |
| *Romboutsia* | rs61841503 | G | A | 0.168 | 0.093 | 0.017 | 4.00E-08 |
| *Romboutsia* | rs62504452 | A | G | 0.176 | -0.071 | 0.016 | 4.66E-06 |
| *Romboutsia* | rs7109293 | A | G | 0.078 | 0.092 | 0.021 | 6.98E-06 |
| *Romboutsia* | rs75200530 | T | G | 0.051 | -0.191 | 0.042 | 5.07E-06 |
| *Romboutsia* | rs75987356 | G | A | 0.068 | -0.13 | 0.028 | 6.71E-06 |
| *Romboutsia* | rs77702691 | A | G | 0.103 | -0.094 | 0.021 | 7.37E-06 |
| *Romboutsia* | rs9567264 | C | T | 0.305 | 0.058 | 0.013 | 5.76E-06 |
| *Roseburia* | rs12740451 | T | C | 0.141 | 0.07 | 0.015 | 7.34E-06 |
| *Roseburia* | rs16910295 | T | C | 0.073 | -0.098 | 0.021 | 2.91E-06 |
| *Roseburia* | rs302266 | T | C | 0.111 | -0.078 | 0.017 | 8.13E-06 |
| *Roseburia* | rs329182 | T | C | 0.14 | 0.069 | 0.015 | 5.90E-06 |
| *Roseburia* | rs55858165 | A | C | 0.054 | 0.179 | 0.04 | 9.99E-06 |
| *Roseburia* | rs57466170 | C | T | 0.09 | 0.074 | 0.017 | 8.30E-06 |
| *Roseburia* | rs6930661 | C | T | 0.075 | -0.096 | 0.02 | 2.48E-06 |
| *Roseburia* | rs75326254 | C | T | 0.064 | -0.105 | 0.023 | 7.50E-06 |
| *Roseburia* | rs78753150 | A | C | 0.061 | 0.097 | 0.021 | 9.98E-06 |
| *Roseburia* | rs9300744 | C | T | 0.238 | -0.059 | 0.013 | 4.75E-06 |
| *Ruminiclostridium 5* | rs10827477 | A | G | 0.322 | -0.055 | 0.012 | 2.19E-06 |
| *Ruminiclostridium 5* | rs1223978 | T | C | 0.449 | 0.048 | 0.011 | 8.16E-06 |
| *Ruminiclostridium 5* | rs1492620 | T | C | 0.095 | -0.083 | 0.018 | 3.53E-06 |
| *Ruminiclostridium 5* | rs4955951 | A | G | 0.111 | -0.071 | 0.017 | 9.96E-06 |
| *Ruminiclostridium 5* | rs6121460 | G | A | 0.073 | 0.093 | 0.02 | 2.64E-06 |
| *Ruminiclostridium 5* | rs79968837 | A | G | 0.084 | -0.095 | 0.019 | 1.15E-06 |
| *Ruminiclostridium 5* | rs8053158 | A | G | 0.121 | -0.074 | 0.016 | 5.90E-06 |
| *Ruminiclostridium 6* | rs10829821 | T | C | 0.102 | -0.098 | 0.022 | 3.47E-06 |
| *Ruminiclostridium 6* | rs116969552 | A | G | 0.048 | -0.167 | 0.038 | 9.16E-06 |
| *Ruminiclostridium 6* | rs11992182 | A | C | 0.273 | 0.063 | 0.014 | 4.65E-06 |
| *Ruminiclostridium 6* | rs1756364 | G | T | 0.112 | 0.1 | 0.02 | 2.54E-07 |
| *Ruminiclostridium 6* | rs35362464 | C | A | 0.17 | 0.072 | 0.017 | 8.99E-06 |
| *Ruminiclostridium 6* | rs61060922 | T | G | 0.054 | 0.159 | 0.032 | 1.09E-06 |
| *Ruminiclostridium 6* | rs663262 | T | C | 0.052 | -0.135 | 0.031 | 3.39E-06 |
| *Ruminiclostridium 6* | rs67479537 | T | C | 0.076 | 0.119 | 0.026 | 9.30E-06 |
| *Ruminiclostridium 6* | rs71414120 | T | G | 0.059 | 0.201 | 0.041 | 1.08E-06 |
| *Ruminiclostridium 6* | rs72991535 | T | G | 0.058 | 0.136 | 0.03 | 4.95E-06 |
| *Ruminiclostridium 6* | rs73176030 | T | C | 0.328 | 0.059 | 0.013 | 7.29E-06 |
| *Ruminiclostridium 6* | rs77193512 | A | G | 0.189 | 0.074 | 0.015 | 1.30E-06 |
| *Ruminiclostridium 6* | rs792058 | G | A | 0.44 | 0.055 | 0.013 | 8.58E-06 |
| *Ruminiclostridium 6* | rs79968172 | G | A | 0.059 | 0.116 | 0.024 | 1.66E-06 |
| *Ruminiclostridium 6* | rs9555756 | A | C | 0.138 | -0.08 | 0.018 | 7.10E-06 |
| *Ruminiclostridium 9* | rs115044523 | G | A | 0.086 | -0.098 | 0.02 | 2.37E-06 |
| *Ruminiclostridium 9* | rs6082461 | A | C | 0.213 | 0.059 | 0.013 | 4.87E-06 |
| *Ruminiclostridium 9* | rs7137760 | C | T | 0.405 | 0.051 | 0.011 | 7.07E-06 |
| *Ruminiclostridium 9* | rs74303178 | T | C | 0.293 | 0.053 | 0.012 | 7.92E-06 |
| *Ruminiclostridium 9* | rs78191726 | T | C | 0.089 | 0.094 | 0.021 | 7.58E-06 |
| *Ruminiclostridium 9* | rs918449 | A | G | 0.071 | -0.095 | 0.02 | 2.56E-06 |
| *Ruminiclostridium 9* | rs9522712 | T | C | 0.157 | 0.07 | 0.015 | 4.66E-06 |
| *Ruminiclostridium 9* | rs9809789 | C | T | 0.146 | -0.072 | 0.016 | 8.72E-06 |
| *Ruminococcaceae NK4A214* | rs11586410 | G | A | 0.125 | -0.086 | 0.017 | 3.66E-07 |
| *Ruminococcaceae NK4A214* | rs13087692 | T | G | 0.311 | 0.057 | 0.013 | 8.69E-06 |
| *Ruminococcaceae NK4A214* | rs136761 | G | A | 0.392 | -0.059 | 0.012 | 8.15E-07 |
| *Ruminococcaceae NK4A214* | rs147475196 | A | G | 0.061 | -0.134 | 0.03 | 4.72E-06 |
| *Ruminococcaceae NK4A214* | rs4814689 | C | T | 0.083 | -0.108 | 0.023 | 4.55E-06 |
| *Ruminococcaceae NK4A214* | rs5994253 | A | G | 0.164 | -0.081 | 0.016 | 2.35E-07 |
| *Ruminococcaceae NK4A214* | rs62027366 | T | C | 0.229 | 0.062 | 0.014 | 6.58E-06 |
| *Ruminococcaceae NK4A214* | rs6681678 | C | T | 0.053 | -0.1 | 0.024 | 9.05E-06 |
| *Ruminococcaceae NK4A214* | rs7573569 | T | C | 0.058 | 0.108 | 0.023 | 3.23E-06 |
| *Ruminococcaceae UCG-002* | rs10916131 | C | T | 0.156 | -0.069 | 0.015 | 2.87E-06 |
| *Ruminococcaceae UCG-002* | rs10927423 | C | A | 0.158 | -0.071 | 0.015 | 8.50E-07 |
| *Ruminococcaceae UCG-002* | rs10964441 | G | A | 0.051 | -0.149 | 0.034 | 7.45E-06 |
| *Ruminococcaceae UCG-002* | rs11607472 | A | G | 0.111 | -0.078 | 0.018 | 7.19E-06 |
| *Ruminococcaceae UCG-002* | rs116974815 | C | A | 0.057 | -0.19 | 0.04 | 2.03E-06 |
| *Ruminococcaceae UCG-002* | rs11750293 | G | T | 0.276 | -0.058 | 0.012 | 1.76E-06 |
| *Ruminococcaceae UCG-002* | rs12463378 | A | G | 0.382 | -0.052 | 0.011 | 2.96E-06 |
| *Ruminococcaceae UCG-002* | rs15256 | C | T | 0.095 | 0.073 | 0.017 | 9.46E-06 |
| *Ruminococcaceae UCG-002* | rs56030423 | G | A | 0.084 | -0.098 | 0.022 | 6.30E-06 |
| *Ruminococcaceae UCG-002* | rs57079348 | T | G | 0.11 | -0.077 | 0.017 | 7.22E-06 |
| *Ruminococcaceae UCG-002* | rs6793778 | C | T | 0.242 | -0.056 | 0.013 | 9.81E-06 |
| *Ruminococcaceae UCG-002* | rs7120052 | A | C | 0.173 | 0.062 | 0.014 | 1.97E-06 |
| *Ruminococcaceae UCG-002* | rs76847269 | A | G | 0.057 | 0.164 | 0.036 | 5.17E-06 |
| *Ruminococcaceae UCG-002* | rs77564310 | A | C | 0.199 | -0.071 | 0.014 | 3.29E-07 |
| *Ruminococcaceae UCG-002* | rs79016051 | C | T | 0.094 | -0.089 | 0.019 | 2.34E-06 |
| *Ruminococcaceae UCG-002* | rs882348 | A | G | 0.097 | -0.08 | 0.018 | 5.45E-06 |
| *Ruminococcaceae UCG-003* | rs10490280 | C | T | 0.243 | -0.067 | 0.014 | 4.16E-06 |
| *Ruminococcaceae UCG-003* | rs11243416 | T | C | 0.099 | -0.093 | 0.019 | 1.67E-06 |
| *Ruminococcaceae UCG-003* | rs16959793 | A | C | 0.277 | -0.063 | 0.013 | 2.22E-06 |
| *Ruminococcaceae UCG-003* | rs2523124 | T | C | 0.375 | -0.055 | 0.012 | 5.78E-06 |
| *Ruminococcaceae UCG-003* | rs3013089 | G | A | 0.404 | -0.055 | 0.012 | 4.38E-06 |
| *Ruminococcaceae UCG-003* | rs4452755 | A | C | 0.279 | -0.063 | 0.013 | 3.29E-06 |
| *Ruminococcaceae UCG-003* | rs4532474 | G | A | 0.127 | 0.077 | 0.017 | 4.82E-06 |
| *Ruminococcaceae UCG-003* | rs6759615 | A | G | 0.077 | 0.103 | 0.02 | 7.86E-07 |
| *Ruminococcaceae UCG-004* | rs10976229 | T | G | 0.135 | 0.096 | 0.021 | 7.04E-06 |
| *Ruminococcaceae UCG-004* | rs11961899 | G | A | 0.29 | -0.071 | 0.016 | 9.18E-06 |
| *Ruminococcaceae UCG-004* | rs2248146 | T | C | 0.326 | 0.069 | 0.015 | 8.20E-06 |
| *Ruminococcaceae UCG-004* | rs511258 | G | A | 0.272 | -0.076 | 0.016 | 4.52E-06 |
| *Ruminococcaceae UCG-004* | rs550351 | A | C | 0.221 | 0.079 | 0.018 | 9.43E-06 |
| *Ruminococcaceae UCG-004* | rs6769553 | A | G | 0.292 | 0.085 | 0.016 | 7.91E-08 |
| *Ruminococcaceae UCG-004* | rs7569771 | A | G | 0.222 | -0.076 | 0.017 | 8.12E-06 |
| *Ruminococcaceae UCG-004* | rs872501 | G | A | 0.115 | 0.116 | 0.026 | 5.81E-06 |
| *Ruminococcaceae UCG-004* | rs9818949 | G | T | 0.197 | 0.086 | 0.019 | 5.39E-06 |
| *Ruminococcaceae UCG-005* | rs10873449 | T | C | 0.164 | 0.065 | 0.014 | 4.11E-06 |
| *Ruminococcaceae UCG-005* | rs10937802 | G | A | 0.104 | 0.076 | 0.017 | 8.17E-06 |
| *Ruminococcaceae UCG-005* | rs10950694 | T | C | 0.415 | 0.058 | 0.011 | 4.30E-07 |
| *Ruminococcaceae UCG-005* | rs114279581 | A | G | 0.061 | -0.147 | 0.032 | 3.22E-06 |
| *Ruminococcaceae UCG-005* | rs12458218 | T | C | 0.171 | 0.068 | 0.014 | 2.41E-06 |
| *Ruminococcaceae UCG-005* | rs2893871 | G | A | 0.153 | -0.074 | 0.016 | 3.54E-06 |
| *Ruminococcaceae UCG-005* | rs34781347 | G | A | 0.055 | 0.189 | 0.039 | 6.05E-07 |
| *Ruminococcaceae UCG-005* | rs72776570 | C | A | 0.096 | 0.087 | 0.02 | 5.36E-06 |
| *Ruminococcaceae UCG-005* | rs7449320 | C | A | 0.221 | 0.06 | 0.013 | 4.81E-06 |
| *Ruminococcaceae UCG-005* | rs7555878 | A | G | 0.261 | 0.059 | 0.013 | 2.81E-06 |
| *Ruminococcaceae UCG-005* | rs898577 | T | C | 0.061 | -0.123 | 0.029 | 7.46E-06 |
| *Ruminococcaceae UCG-009* | rs113006825 | T | C | 0.214 | -0.093 | 0.021 | 7.98E-06 |
| *Ruminococcaceae UCG-009* | rs12508214 | C | T | 0.379 | -0.077 | 0.017 | 4.75E-06 |
| *Ruminococcaceae UCG-009* | rs138460696 | A | G | 0.08 | 0.139 | 0.032 | 9.81E-06 |
| *Ruminococcaceae UCG-009* | rs1550196 | G | A | 0.097 | 0.131 | 0.026 | 1.13E-06 |
| *Ruminococcaceae UCG-009* | rs2058609 | A | G | 0.346 | 0.082 | 0.017 | 3.12E-06 |
| *Ruminococcaceae UCG-009* | rs2192926 | A | G | 0.234 | -0.089 | 0.019 | 4.88E-06 |
| *Ruminococcaceae UCG-009* | rs4079028 | C | T | 0.208 | 0.092 | 0.02 | 3.28E-06 |
| *Ruminococcaceae UCG-009* | rs4708333 | T | G | 0.313 | -0.084 | 0.017 | 1.56E-06 |
| *Ruminococcaceae UCG-009* | rs6952765 | G | A | 0.442 | 0.073 | 0.017 | 8.13E-06 |
| *Ruminococcaceae UCG-009* | rs758191 | T | G | 0.056 | 0.177 | 0.038 | 9.01E-06 |
| *Ruminococcaceae UCG-009* | rs78410648 | A | G | 0.09 | 0.121 | 0.028 | 9.67E-06 |
| *Ruminococcaceae UCG-009* | rs9558661 | T | C | 0.201 | -0.09 | 0.02 | 7.01E-06 |
| *Ruminococcaceae UCG-010* | rs12597105 | G | A | 0.272 | 0.067 | 0.014 | 4.87E-06 |
| *Ruminococcaceae UCG-010* | rs2820282 | A | C | 0.463 | -0.059 | 0.013 | 2.85E-06 |
| *Ruminococcaceae UCG-010* | rs682403 | A | G | 0.497 | -0.059 | 0.012 | 2.37E-06 |
| *Ruminococcaceae UCG-010* | rs6958419 | C | T | 0.477 | -0.059 | 0.012 | 2.84E-06 |
| *Ruminococcaceae UCG-010* | rs73218807 | G | A | 0.049 | -0.166 | 0.037 | 6.43E-06 |
| *Ruminococcaceae UCG-010* | rs7441445 | C | T | 0.419 | -0.057 | 0.013 | 6.80E-06 |
| *Ruminococcaceae UCG-011* | rs12724320 | C | T | 0.315 | -0.121 | 0.025 | 1.52E-06 |
| *Ruminococcaceae UCG-011* | rs1416041 | A | C | 0.131 | -0.182 | 0.034 | 7.04E-08 |
| *Ruminococcaceae UCG-011* | rs2729556 | C | T | 0.528 | -0.109 | 0.023 | 3.19E-06 |
| *Ruminococcaceae UCG-011* | rs9729514 | A | G | 0.091 | 0.185 | 0.039 | 2.37E-06 |
| *Ruminococcaceae UCG-013* | rs12189346 | G | A | 0.148 | 0.068 | 0.015 | 1.68E-06 |
| *Ruminococcaceae UCG-013* | rs12336782 | T | C | 0.099 | -0.086 | 0.019 | 8.60E-06 |
| *Ruminococcaceae UCG-013* | rs12485353 | G | A | 0.21 | -0.061 | 0.013 | 4.19E-06 |
| *Ruminococcaceae UCG-013* | rs12781711 | C | T | 0.346 | -0.066 | 0.012 | 2.55E-08 |
| *Ruminococcaceae UCG-013* | rs16918863 | A | C | 0.06 | 0.111 | 0.024 | 4.16E-06 |
| *Ruminococcaceae UCG-013* | rs2730183 | G | A | 0.469 | -0.049 | 0.011 | 8.44E-06 |
| *Ruminococcaceae UCG-013* | rs4385846 | G | T | 0.232 | 0.06 | 0.013 | 6.46E-06 |
| *Ruminococcaceae UCG-013* | rs75088940 | T | C | 0.09 | -0.094 | 0.02 | 2.55E-06 |
| *Ruminococcaceae UCG-013* | rs76973485 | G | T | 0.056 | 0.195 | 0.042 | 3.35E-06 |
| *Ruminococcaceae UCG-013* | rs7784330 | G | A | 0.387 | -0.05 | 0.011 | 8.16E-06 |
| *Ruminococcaceae UCG-013* | rs9313055 | T | C | 0.057 | 0.105 | 0.023 | 9.55E-06 |
| *Ruminococcaceae UCG-014* | rs10495392 | C | T | 0.115 | -0.082 | 0.019 | 9.96E-06 |
| *Ruminococcaceae UCG-014* | rs10791168 | A | G | 0.179 | -0.066 | 0.015 | 9.76E-06 |
| *Ruminococcaceae UCG-014* | rs10941294 | C | T | 0.07 | -0.122 | 0.026 | 2.40E-06 |
| *Ruminococcaceae UCG-014* | rs115777838 | T | C | 0.051 | -0.188 | 0.039 | 4.62E-07 |
| *Ruminococcaceae UCG-014* | rs12638134 | T | G | 0.405 | 0.058 | 0.012 | 1.21E-06 |
| *Ruminococcaceae UCG-014* | rs34402072 | C | T | 0.188 | -0.069 | 0.016 | 9.80E-06 |
| *Ruminococcaceae UCG-014* | rs56105232 | G | A | 0.06 | 0.139 | 0.03 | 2.91E-06 |
| *Ruminococcaceae UCG-014* | rs72809222 | T | C | 0.262 | 0.067 | 0.014 | 2.41E-06 |
| *Ruminococcaceae UCG-014* | rs73186226 | G | A | 0.082 | -0.099 | 0.022 | 6.72E-06 |
| *Ruminococcaceae UCG-014* | rs853612 | A | G | 0.403 | -0.053 | 0.012 | 9.75E-06 |
| *Ruminococcaceae UCG-014* | rs995642 | C | T | 0.3 | 0.06 | 0.013 | 1.90E-06 |
| *Ruminococcus 1* | rs11783695 | G | T | 0.134 | -0.073 | 0.016 | 4.73E-06 |
| *Ruminococcus 1* | rs17781867 | C | T | 0.093 | 0.1 | 0.021 | 1.96E-06 |
| *Ruminococcus 1* | rs3819978 | C | T | 0.054 | -0.115 | 0.026 | 8.74E-06 |
| *Ruminococcus 1* | rs6105066 | T | C | 0.235 | -0.061 | 0.013 | 5.06E-06 |
| *Ruminococcus 1* | rs6493760 | C | T | 0.386 | 0.054 | 0.012 | 3.38E-06 |
| *Ruminococcus 1* | rs7117576 | A | G | 0.12 | 0.083 | 0.017 | 6.48E-07 |
| *Ruminococcus 1* | rs7583465 | C | T | 0.403 | 0.053 | 0.011 | 2.56E-06 |
| *Ruminococcus 1* | rs78572139 | G | A | 0.06 | 0.125 | 0.028 | 5.23E-06 |
| *Ruminococcus 1* | rs78613526 | G | A | 0.054 | 0.167 | 0.037 | 5.11E-06 |
| *Ruminococcus 2* | rs12406309 | A | C | 0.202 | -0.063 | 0.014 | 9.79E-06 |
| *Ruminococcus 2* | rs12986628 | C | T | 0.227 | 0.067 | 0.014 | 2.14E-06 |
| *Ruminococcus 2* | rs1819812 | G | T | 0.117 | 0.084 | 0.018 | 5.28E-06 |
| *Ruminococcus 2* | rs2368224 | T | G | 0.031 | 0.2 | 0.044 | 3.63E-06 |
| *Ruminococcus 2* | rs2846589 | G | T | 0.467 | 0.052 | 0.012 | 7.59E-06 |
| *Ruminococcus 2* | rs2997412 | A | G | 0.338 | -0.057 | 0.012 | 4.22E-06 |
| *Ruminococcus 2* | rs4400279 | A | G | 0.367 | 0.055 | 0.012 | 5.80E-06 |
| *Ruminococcus 2* | rs4799823 | C | T | 0.111 | 0.084 | 0.018 | 5.40E-06 |
| *Ruminococcus 2* | rs55707116 | C | A | 0.102 | 0.087 | 0.019 | 8.01E-06 |
| *Ruminococcus 2* | rs58681734 | A | G | 0.146 | 0.072 | 0.016 | 4.18E-06 |
| *Ruminococcus 2* | rs61791565 | T | C | 0.419 | -0.052 | 0.012 | 6.79E-06 |
| *Ruminococcus 2* | rs75140805 | T | G | 0.134 | 0.084 | 0.018 | 3.95E-06 |
| *Ruminococcus 2* | rs7635831 | G | A | 0.297 | 0.062 | 0.013 | 1.98E-06 |
| *Ruminococcus 2* | rs7693984 | G | A | 0.061 | -0.103 | 0.024 | 9.42E-06 |
| *Ruminococcus 2* | rs78120384 | A | G | 0.048 | -0.193 | 0.039 | 3.31E-07 |
| *Sellimonas* | rs13417181 | T | C | 0.205 | 0.167 | 0.034 | 7.62E-07 |
| *Sellimonas* | rs2187447 | A | C | 0.069 | 0.243 | 0.053 | 3.98E-06 |
| *Sellimonas* | rs2371572 | A | C | 0.529 | 0.127 | 0.025 | 4.46E-07 |
| *Sellimonas* | rs41816 | A | G | 0.257 | 0.132 | 0.029 | 8.39E-06 |
| *Sellimonas* | rs4600608 | A | G | 0.223 | -0.137 | 0.03 | 4.95E-06 |
| *Sellimonas* | rs553697 | T | C | 0.172 | -0.154 | 0.034 | 6.13E-06 |
| *Sellimonas* | rs56203279 | T | C | 0.32 | -0.124 | 0.027 | 3.72E-06 |
| *Senegalimassilia* | rs10036909 | C | T | 0.07 | 0.186 | 0.04 | 8.05E-06 |
| *Senegalimassilia* | rs11787826 | C | A | 0.454 | 0.081 | 0.017 | 2.63E-06 |
| *Senegalimassilia* | rs1990708 | A | C | 0.136 | -0.11 | 0.025 | 8.91E-06 |
| *Senegalimassilia* | rs2017373 | C | T | 0.37 | 0.078 | 0.018 | 9.50E-06 |
| *Senegalimassilia* | rs7225245 | G | A | 0.472 | 0.079 | 0.017 | 4.18E-06 |
| *Slackia* | rs10409783 | A | G | 0.268 | 0.095 | 0.021 | 7.70E-06 |
| *Slackia* | rs12440440 | A | G | 0.338 | 0.09 | 0.019 | 2.63E-06 |
| *Slackia* | rs16894137 | C | T | 0.134 | -0.123 | 0.026 | 2.71E-06 |
| *Slackia* | rs35156985 | T | C | 0.064 | -0.156 | 0.035 | 8.06E-06 |
| *Slackia* | rs4492265 | A | G | 0.339 | -0.091 | 0.019 | 2.41E-06 |
| *Slackia* | rs8901 | C | T | 0.384 | 0.093 | 0.019 | 6.07E-07 |
| *Streptococcus* | rs10028567 | C | T | 0.093 | -0.092 | 0.019 | 7.30E-06 |
| *Streptococcus* | rs10448310 | A | G | 0.435 | -0.052 | 0.011 | 3.31E-06 |
| *Streptococcus* | rs11110281 | T | C | 0.063 | -0.138 | 0.023 | 2.58E-09 |
| *Streptococcus* | rs11720390 | G | A | 0.078 | 0.107 | 0.023 | 3.59E-06 |
| *Streptococcus* | rs11764382 | A | G | 0.181 | -0.07 | 0.014 | 1.29E-06 |
| *Streptococcus* | rs1918540 | G | A | 0.262 | 0.06 | 0.013 | 2.44E-06 |
| *Streptococcus* | rs2370083 | G | T | 0.103 | -0.082 | 0.019 | 9.75E-06 |
| *Streptococcus* | rs4968759 | A | G | 0.432 | -0.052 | 0.011 | 3.78E-06 |
| *Streptococcus* | rs57646748 | G | A | 0.07 | -0.091 | 0.02 | 5.48E-06 |
| *Streptococcus* | rs6806351 | T | C | 0.232 | -0.063 | 0.014 | 4.94E-06 |
| *Streptococcus* | rs71481756 | T | G | 0.092 | 0.093 | 0.021 | 6.51E-06 |
| *Streptococcus* | rs7916711 | A | G | 0.058 | 0.103 | 0.022 | 2.72E-06 |
| *Streptococcus* | rs9903102 | C | A | 0.142 | -0.071 | 0.016 | 4.18E-06 |
| *Subdoligranulum* | rs10065321 | T | C | 0.417 | -0.051 | 0.011 | 2.10E-06 |
| *Subdoligranulum* | rs10497836 | C | T | 0.267 | -0.052 | 0.012 | 8.38E-06 |
| *Subdoligranulum* | rs1667315 | G | A | 0.43 | 0.049 | 0.011 | 6.72E-06 |
| *Subdoligranulum* | rs2114677 | C | T | 0.056 | -0.104 | 0.023 | 2.72E-06 |
| *Subdoligranulum* | rs2171249 | C | T | 0.079 | 0.107 | 0.023 | 4.51E-06 |
| *Subdoligranulum* | rs4347804 | A | G | 0.044 | 0.166 | 0.036 | 2.18E-06 |
| *Subdoligranulum* | rs6555306 | T | C | 0.124 | -0.074 | 0.016 | 2.81E-06 |
| *Subdoligranulum* | rs75158211 | T | C | 0.13 | -0.072 | 0.016 | 7.52E-06 |
| *Subdoligranulum* | rs76528319 | G | T | 0.045 | -0.143 | 0.031 | 7.41E-06 |
| *Sutterella* | rs1145877 | A | G | 0.149 | -0.074 | 0.016 | 7.20E-06 |
| *Sutterella* | rs11591622 | T | G | 0.245 | -0.069 | 0.015 | 6.50E-06 |
| *Sutterella* | rs13173038 | A | G | 0.208 | -0.072 | 0.015 | 2.73E-06 |
| *Sutterella* | rs143438747 | T | C | 0.063 | -0.146 | 0.031 | 3.28E-06 |
| *Sutterella* | rs2050185 | G | A | 0.372 | 0.058 | 0.013 | 7.97E-06 |
| *Sutterella* | rs2613606 | C | T | 0.434 | -0.056 | 0.012 | 7.20E-06 |
| *Sutterella* | rs607327 | C | T | 0.371 | 0.058 | 0.013 | 6.63E-06 |
| *Sutterella* | rs62501473 | G | A | 0.247 | 0.069 | 0.015 | 5.52E-06 |
| *Sutterella* | rs7499539 | A | G | 0.302 | 0.062 | 0.013 | 2.36E-06 |
| *Sutterella* | rs7638039 | T | C | 0.245 | 0.065 | 0.014 | 8.66E-06 |
| *Sutterella* | rs9350083 | T | G | 0.305 | -0.059 | 0.013 | 8.23E-06 |
| *Terrisporobacter* | rs1883097 | C | T | 0.059 | 0.226 | 0.045 | 4.16E-07 |
| *Terrisporobacter* | rs2569953 | A | C | 0.45 | -0.078 | 0.017 | 8.95E-06 |
| *Terrisporobacter* | rs2872237 | C | A | 0.408 | -0.081 | 0.018 | 3.97E-06 |
| *Terrisporobacter* | rs58405430 | G | T | 0.105 | 0.135 | 0.03 | 7.94E-06 |
| *Turicibacter* | rs11054680 | T | C | 0.122 | -0.105 | 0.023 | 2.31E-06 |
| *Turicibacter* | rs11666533 | C | T | 0.114 | -0.112 | 0.025 | 7.37E-06 |
| *Turicibacter* | rs12603364 | T | C | 0.12 | 0.111 | 0.023 | 8.67E-07 |
| *Turicibacter* | rs149744580 | A | G | 0.059 | 0.17 | 0.032 | 7.01E-08 |
| *Turicibacter* | rs2834977 | T | C | 0.151 | -0.096 | 0.021 | 3.96E-06 |
| *Turicibacter* | rs2952020 | G | A | 0.277 | -0.076 | 0.017 | 5.63E-06 |
| *Turicibacter* | rs3734633 | G | A | 0.085 | -0.121 | 0.027 | 5.32E-06 |
| *Turicibacter* | rs4869133 | G | A | 0.083 | 0.131 | 0.027 | 2.55E-06 |
| *Turicibacter* | rs55756211 | T | C | 0.105 | -0.115 | 0.024 | 2.81E-06 |
| *Turicibacter* | rs7199484 | G | A | 0.309 | -0.073 | 0.016 | 5.77E-06 |
| *Tyzzerella 3* | rs10898797 | C | T | 0.152 | 0.122 | 0.027 | 8.85E-06 |
| *Tyzzerella 3* | rs1232220 | G | T | 0.131 | -0.144 | 0.032 | 7.91E-06 |
| *Tyzzerella 3* | rs17706273 | T | C | 0.138 | -0.14 | 0.027 | 5.88E-07 |
| *Tyzzerella 3* | rs191093 | G | A | 0.088 | 0.159 | 0.035 | 6.76E-06 |
| *Tyzzerella 3* | rs4904512 | T | C | 0.181 | -0.117 | 0.025 | 3.09E-06 |
| *Tyzzerella 3* | rs55799124 | A | G | 0.229 | -0.114 | 0.024 | 1.34E-06 |
| *Tyzzerella 3* | rs67476743 | T | G | 0.301 | 0.132 | 0.022 | 3.74E-09 |
| *Tyzzerella 3* | rs6920448 | C | T | 0.122 | -0.141 | 0.031 | 4.15E-06 |
| *Tyzzerella 3* | rs7019909 | T | C | 0.103 | 0.144 | 0.03 | 1.76E-06 |
| *Tyzzerella 3* | rs7333521 | T | C | 0.055 | -0.207 | 0.045 | 4.88E-06 |
| *Tyzzerella 3* | rs75091807 | G | T | 0.086 | -0.185 | 0.038 | 1.71E-06 |
| *Veillonella* | rs11141494 | G | A | 0.234 | -0.078 | 0.017 | 9.75E-06 |
| *Veillonella* | rs1882878 | A | G | 0.263 | -0.077 | 0.016 | 2.98E-06 |
| *Veillonella* | rs2013594 | T | C | 0.33 | -0.072 | 0.016 | 3.42E-06 |
| *Veillonella* | rs55807413 | A | G | 0.127 | 0.107 | 0.024 | 5.51E-06 |
| *Veillonella* | rs62376424 | C | T | 0.342 | -0.076 | 0.016 | 3.65E-06 |
| *Veillonella* | rs6656807 | A | G | 0.404 | 0.07 | 0.015 | 5.50E-06 |
| *Veillonella* | rs7359080 | C | A | 0.123 | -0.135 | 0.03 | 7.40E-06 |
| *Veillonella* | rs742016 | A | G | 0.417 | -0.069 | 0.015 | 4.66E-06 |
| *Victivallis* | rs11899949 | G | A | 0.255 | 0.131 | 0.028 | 2.77E-06 |
| *Victivallis* | rs12512543 | A | C | 0.118 | -0.178 | 0.037 | 2.54E-06 |
| *Victivallis* | rs173120 | T | C | 0.244 | 0.134 | 0.029 | 7.65E-06 |
| *Victivallis* | rs1882775 | A | G | 0.205 | -0.138 | 0.031 | 8.73E-06 |
| *Victivallis* | rs2546432 | T | C | 0.471 | -0.111 | 0.025 | 9.93E-06 |
| *Victivallis* | rs342302 | A | G | 0.153 | -0.153 | 0.035 | 8.16E-06 |
| *Victivallis* | rs4764863 | G | A | 0.505 | 0.122 | 0.025 | 8.22E-07 |
| *Victivallis* | rs4895919 | T | C | 0.469 | -0.117 | 0.025 | 2.75E-06 |
| *Victivallis* | rs56349194 | A | G | 0.17 | -0.159 | 0.032 | 6.26E-07 |
| *Victivallis* | rs911666 | T | C | 0.327 | -0.119 | 0.026 | 7.65E-06 |


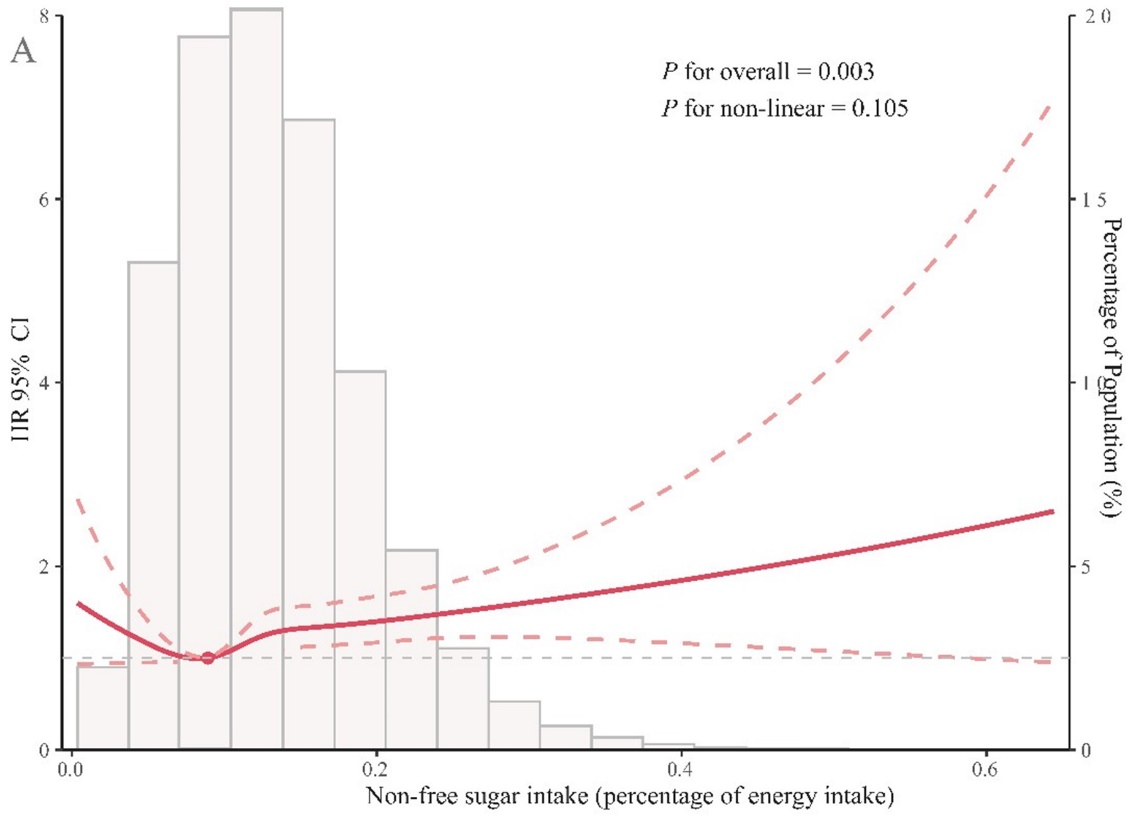

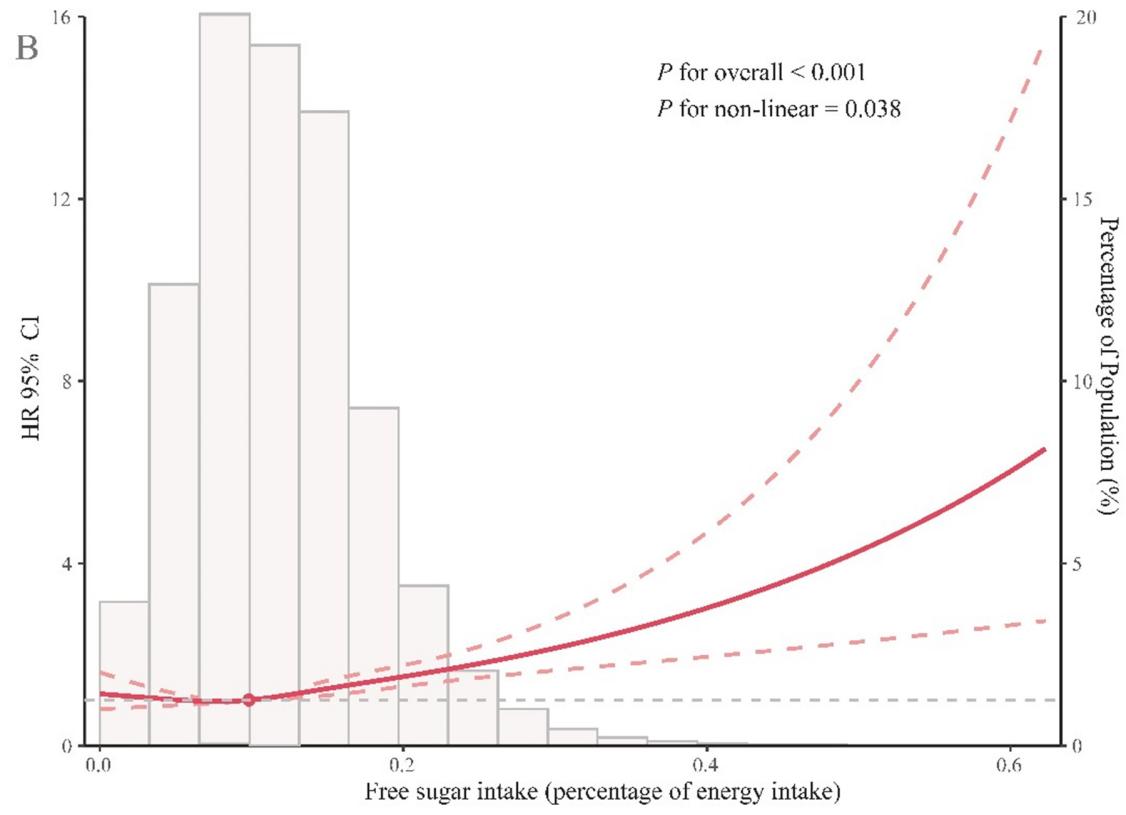


**Supplementary Fig 1. Restricted cubic splines of the associations of dementia risk with non-free sugar (A) and sugar (B) intake.** Knots were placed at the 5th, 35th, 65th 95th percentiles of the independent variables’ distributions, and the reference value was set at the 5th percentile. The model adjusted for age at recruitment, sex, race, education level, average total household income before tax, Townsend Deprivation Index, physical activity, alcohol consumption status, smoking status, intakes of protein and fat (percentage of energy intake), total energy intake, history of hypertension, diabetes, and cardiovascular disease, and family history of dementia (free sugar intake or non-free sugar intake were mutually adjusted for each other) (n = 158,408).

*Abbreviations:* CIs, confidence intervals; HRs, hazard ratios.

**Supplementary Table 3. Sensitivity analysis of the associations between non-free and free sugar inake and incident dementia with including BMI as a covariate (n = 158,408) ^a^**

| **Sugar intake** | **Cases (%)** | **HR (95% CI)** |
| --- | --- | --- |
| **Quartile** |  |  |
| <8.62% | 241 (0.61) | Reference |
| ≥8.62% and <12.11% | 279 (0.70) | 1.02 (0.85, 1.21) |
| ≥12.11% and <16.32% | 330 (0.83) | 1.14 (0.96, 1.36) |
| ≥16.32% | 369 (0.93) | 1.25 (1.03, 1.52) |
| ***P*-trend ^b^** |  | 0.009 |
| **Continuous (per 5%** **energy increase)** |  | 1.10 (1.04, 1.16) |
| **Free sugar intakes (% energy intake)** |  |  |
| **Quartile** |  |  |
| <7.87% | 280 (0.71) | Reference |
| ≥7.87% and <11.08% | 279 (0.70) | 1.06 (0.90. 1.26) |
| ≥11.08% and <14.78% | 294 (0.74) | 1.11 (0.93, 1.32) |
| ≥14.78% | 366 (0.92) | 1.43 (1.20, 1.70) |
| ***P*-trend ^b^** |  | <0.0001 |
| **Continuous (per 5% energy increase)** |  | 1.17 (1.10, 1.23) |

*Abbreviations:* BMI, body mass index; CIs, confidence intervals; HRs, hazard ratios. ^a^ HRs and 95% CIs were calculated by the cause-specific competing risk model. ^b^ Test for trend based on variables containing the median value for each quartile. The model was the age at recruitment, sex, race, education level, average total household income before tax, Townsend Deprivation Index, physical activity, alcohol consumption status, smoking status, intakes of protein and fat (percentage of energy intake), total energy intake, history of hypertension, diabetes, and cardiovascular diseases, family history of dementia and BMI (free sugar intake or non-free sugar intake were mutually adjusted for each other).

**Supplementary Table 4. Associations of** **non-free** **and free sugar intake with incident dementia by excluding participants with less than two years of follow-up (n = 157,541) ^a^**

| **Sugar intake** | **Cases (%)** | **HR (95% CI)** | | |
| --- | --- | --- | --- | --- |
|  |  | **Model 1 ^c^** | **Model 2 ^d^** | **Model 3 ^e^** |
| **Non-free sugar intakes (% energy intake)** | | | | |
| **Quartile** |  |  |  |  |
| <8.63% | 237 (0.60) | Reference | Reference | Reference |
| ≥8.63% and <12.11% | 271 (0.69) | 1.14 (0.95, 1.35) | 0.99 (0.83, 1.18) | 1.01 (0.84, 1.20) |
| ≥12.11% and <16.32% | 318 (0.81) | 1.33 (1.12, 1.57) | 1.10 (0.92, 1.31) | 1.12 (0.94, 1.34) |
| ≥16.32% | 356 (0.90) | 1.47 (1.25, 1.74) | 1.20 (1.00, 1.46) | 1.24 (1.02, 1.50) |
| ***P*-trend ^b^** |  | < 0.0001 | 0.03 | 0.01 |
| **Continuous (per 5% energy increase)** |  | 1.12 (1.07, 1.17) | 1.08 (1.02, 1.14) | 1.09 (1.03, 1.16) |
| **Free sugar intakes** **(%** **energy intake)** |  |  |  |  |
| **Quartile** |  |  |  |  |
| <7.87% | 271 (0.69) | Reference | Reference | Reference |
| ≥7.87% and <11.07% | 272 (0.69) | 1.00 (0.85, 1.19) | 1.04 (0.88, 1.24) | 1.07 (0.90, 1.27) |
| ≥11.07% and <14.77% | 286 (0.73) | 1.06 (0.90, 1.25) | 1.07 (0.90, 1.28) | 1.12 (0.94, 1.33) |
| ≥14.77% | 353 (0.90) | 1.31 (1.11, 1.53) | 1.36 (1.13, 1.62) | 1.42 (1.19, 1.70) |
| ***P*-trend ^b^** |  | < 0.001 | < 0.001 | < 0.0001 |
| **Continuous (per 5% energy increase)** |  | 1.10 (1.05, 1.15) | 1.14 (1.08, 1.20) | 1.16 (1.09, 1.22) |

*Abbreviations:* CIs, confidence intervals; HRs, hazard ratios. ^a^ HRs and 95% CIs were calculated by the cause-specific competing risk model. ^b^ Test for trend based on variables containing the median value for each quartile. ^c^ Model 1 was the crude model. ^d^ Model 2 was adjusted for age at recruitment, sex, ethnicity, education level, average total household income before tax, Townsend Deprivation Index, physical activity, alcohol consumption status, smoking status, intakes of protein and fat (percentage of energy intake), and total energy intake. ^e^ Model 3 was adjusted for Model 2 + history of hypertension, diabetes, and cardiovascular diseases, and family history of dementia (free sugar intake or non-free sugar intake were mutually adjusted for each other).

**Supplementary Table 5. Associations of non-free and free sugar intake with incident dementia by excluding participants with less than two dietary assessments (n = 97,821) ^a^**

| **Sugar intake** | **Cases (%)** | **HR (95% CI)** | | |
| --- | --- | --- | --- | --- |
|  |  | **Model 1 ^c^** | **Model 2 ^d^** | **Model 3 ^e^** |
| **Non-free sugar intakes (% energy intake)** | | | | |
| **Quartile** |  |  |  |  |
| <8.95% | 123 (0.50) | Reference | Reference | Reference |
| ≥8.95% and <12.14% | 144 (0.59) | 1.15 (0.91, 1.47) | 1.02 (0.80, 1.31) | 1.04 (0.81, 1.32) |
| ≥12.14% and <16.00% | 184 (0.75) | 1.47 (1.17, 1.85) | 1.26 (0.99, 1.61) | 1.28 (1.01, 1.64) |
| ≥16.00% | 188 (0.77) | 1.50 (1.19, 1.88) | 1.32 (1.01, 1.72) | 1.38 (1.05, 1.80) |
| ***P*-trend ^b^** |  | < 0.001 | 0.02 | < 0.01 |
| **Continuous (per 5% energy increase)** |  | 1.13 (1.06, 1.21) | 1.12 (1.03, 1.22) | 1.14 (1.04, 1.24) |
| **Free sugar intakes (% energy intake)** |  |  |  |  |
| **Quartile** |  |  |  |  |
| <8.17% | 137 (0.56) | Reference | Reference | Reference |
| ≥8.17% and <11.07% | 153 (0.63) | 1.12 (0.89, 1.41) | 1.15 (0.91, 1.46) | 1.20 (0.95, 1.53) |
| ≥11.07% and <14.42% | 157 (0.64) | 1.15 (0.91, 1.44) | 1.18 (0.93, 1.50) | 1.25 (0.98, 1.59) |
| ≥14.42% | 192 (0.79) | 1.41 (1.13, 1.76) | 1.51 (1.18, 1.94) | 1.63 (1.27, 2.10) |
| ***P*-trend ^b^** |  | < 0.01 | < 0.01 | < 0.001 |
| **Continuous (per 5% energy increase)** |  | 1.17 (1.08, 1.25) | 1.25 (1.14, 1.36) | 1.28 (1.17, 1.40) |

*Abbreviations:* CIs, confidence intervals; HRs, hazard ratios. ^a^ HRs and 95% CIs were calculated by the cause-specific competing risk model. ^b^ Test for trend based on variables containing the median value for each quartile. ^c^ Model 1 was the crude model. ^d^ Model 2 was adjusted for age at recruitment, sex, ethnicity, education level, average total household income before tax, Townsend Deprivation Index, physical activity, alcohol consumption status, smoking status, intakes of protein and fat (percentage of energy intake), and total energy intake. ^e^ Model 3 was adjusted for Model 2 + history of hypertension, diabetes, and cardiovascular diseases, and family history of dementia (free sugar intake or non-free sugar intake were mutually adjusted for each other).

**Supplementary Table 6. Associations of absolute non-free and free sugar intake with incident dementia (n = 158,408) ^a^**

| **Sugar intake** | **Cases (%)** | **HR (95% CI)** | | |
| --- | --- | --- | --- | --- |
|  |  | **Model 1 ^c^** | **Model 2 ^d^** | **Model 3 ^e^** |
| **Non-free sugar intakes (g/day)** | | | | |
| **Quartile** |  |  |  |  |
| <42.66 | 257 (0.65) | Reference | Reference | Reference |
| ≥42.66 and <60.05 | 256 (0.65) | 0.99 (0.83, 1.18) | 0.86 (0.72, 1.03) | 0.88 (0.74, 1.05) |
| ≥60.05 and <80.33 | 295 (0.74) | 1.13 (0.96, 1.34) | 0.90 (0.76, 1.08) | 0.93 (0.77, 1.11) |
| ≥80.33 | 411 (1.04) | 1.57 (1.35, 1.84) | 1.16 (0.96, 1.39) | 1.19 (1.00, 1.44) |
| ***P*-trend ^b^** |  | < 0.0001 | 0.02 | 0.01 |
| **Continuous (per 5g increase)** |  | 1.03 (1.02, 1.04) | 1.02 (1.01, 1.03) | 1.02 (1.01, 1.03) |
| **Free sugar intakes (g/day)** |  |  |  |  |
| **Quartile** |  |  |  |  |
| <36.22 | 288 (0.73) | Reference | Reference | Reference |
| ≥36.22 and <54.86 | 237 (0.60) | 0.83 (0.70, 0.98) | 0.86 (0.72, 1.03) | 0.88 (0.74, 1.06) |
| ≥54.86 and <78.36 | 317 (0.80) | 1.11 (0.94, 1.30) | 1.15 (0.96, 1.37) | 1.20 (1.01, 1.43) |
| ≥78.36 | 377 (0.95) | 1.32 (1.13, 1.53) | 1.38 (1.13, 1.69) | 1.47 (1.20, 1.79) |
| ***P*-trend ^b^** |  | < 0.0001 | < 0.0001 | < 0.0001 |
| **Continuous (per 5g increase)** |  | 1.02 (1.01, 1.03) | 1.03 (1.01, 1.04) | 1.03 (1.02, 1.04) |

*Abbreviations:* CIs, confidence intervals; HRs, hazard ratios. ^a^ HRs and 95% CIs were calculated by the cause-specific competing risk model. ^b^ Test for trend based on variables containing the median value for each quartile. ^c^ Model 1 was the crude model. ^d^ Model 2 was adjusted for age at recruitment, sex, ethnicity, education level, average total household income before tax, Townsend Deprivation Index, physical activity, alcohol consumption status, smoking status, intakes of protein and fat (percentage of energy intake), and total energy intake. ^e^ Model 3 was adjusted for Model 2 + history of hypertension, diabetes, and cardiovascular diseases, and family history of dementia (free sugar intake or non-free sugar intake were mutually adjusted for each other).

**Supplementary Table 7. Associations of non-free and free sugar to carbohydrates intake ratio with incident dementia (n = 158,408) ^a^**

| **Sugar intake** | **Cases (%)** | **HR (95% CI)** | | |
| --- | --- | --- | --- | --- |
|  |  | **Model 1 ^c^** | **Model 2 ^d^** | **Model 3 ^e^** |
| **Non-free sugars to carbohydrates ratio** | | | | |
| **Quartile** |  |  |  |  |
| <18.17% | 250 (0.63) | Reference | Reference | Reference |
| ≥18.17% and <24.46% | 266 (0.67) | 1.06 (0.89, 1.26) | 0.94 (0.79, 1.12) | 0.95 (0.80, 1.13) |
| ≥24.46% and <31.79% | 354 (0.89) | 1.40 (1.19, 1.65) | 1.20 (1.01, 1.42) | 1.22 (1.03, 1.45) |
| ≥31.79% | 349 (0.88) | 1.37 (1.17, 1.62) | 1.20 (1.00, 1.45) | 1.23 (1.02, 1.48) |
| ***P*-trend ^b^** |  | < 0.0001 | < 0.01 | < 0.01 |
| **Continuous (per 5% increase)** |  | 1.06 (1.03, 1.08) | 1.05 (1.01, 1.08) | 1.05 (1.02, 1.08) |
| **Free sugars to carbohydrates ratio** |  |  |  |  |
| **Quartile** |  |  |  |  |
| <16.24% | 276 (0.70) | Reference | Reference | Reference |
| ≥16.24% and <22.50% | 297 (0.75) | 1.08 (0.91, 1.27) | 1.12 (0.95, 1.33) | 1.16 (0.98, 1.37) |
| ≥22.50% and <29.49% | 311 (0.79) | 1.13 (0.96, 1.33) | 1.20 (1.01, 1.43) | 1.26 (1.06, 1.49) |
| ≥29.49% | 335 (0.85) | 1.23 (1.05, 1.44) | 1.38 (1.16, 1.65) | 1.45 (1.21, 1.74) |
| ***P*-trend ^b^** |  | < 0.01 | < 0.001 | < 0.0001 |
| **Continuous (per 5% increase)** |  | 1.03 (1.01, 1.06) | 1.06 (1.03, 1.10) | 1.07 (1.04, 1.11) |

*Abbreviations:* CIs, confidence intervals; HRs, hazard ratios. ^a^ HRs and 95% CIs were calculated by the cause-specific competing risk model. ^b^ Test for trend based on variables containing the median value for each quartile. ^c^ Model 1 was the crude model. ^d^ Model 2 was adjusted for age at recruitment, sex, ethnicity, education level, average total household income before tax, Townsend Deprivation Index, physical activity, alcohol consumption status, smoking status, intakes of protein and fat (percentage of energy intake), and total energy intake. ^e^ Model 3 was adjusted for Model 2 + history of hypertension, diabetes, and cardiovascular diseases, and family history of dementia (free sugar intake or non-free sugar intake were mutually adjusted for each other).

**Supplementary Table 8. Associations of non-free and free sugar intake with incident dementia using the sub-distribution competing risk model (n = 158,408) ^a^**

| **Sugar intake** | **Cases (%)** | **HR (95% CI)** | | |
| --- | --- | --- | --- | --- |
|  |  | **Model 1 ^c^** | **Model 2 ^d^** | **Model 3 ^e^** |
| **Non-free sugar intakes (% energy intake)** | | | | |
| **Quartile** |  |  |  |  |
| <8.62% | 241 (0.61) | Reference | Reference | Reference |
| ≥8.62% and <12.11% | 279 (0.70) | 1.15 (0.97, 1.37) | 1.01 (0.85, 1.20) | 1.02 (0.86, 1.22) |
| ≥12.11% and <16.32% | 330 (0.83) | 1.36 (1.15, 1.60) | 1.14 (0.95, 1.36) | 1.16 (0.97, 1.39) |
| ≥16.32% | 369 (0.93) | 1.51 (1.28, 1.77) | 1.23 (1.01, 1.51) | 1.27 (1.04, 1.55) |
| ***P*-trend ^b^** |  | < 0.0001 | 0.02 | < 0.01 |
| **Continuous (per 5% energy increase)** |  | 1.12 (1.08, 1.17) | 1.09 (1.03, 1.16) | 1.10 (1.04, 1.16) |
| **Free sugar intakes (% energy intake)** |  |  |  |  |
| **Quartile** |  |  |  |  |
| <7.87% | 280 (0.71) | Reference | Reference | Reference |
| ≥7.87% and <11.08% | 279 (0.70) | 1.00 (0.85, 1.18) | 1.04 (0.88, 1.23) | 1.06 (0.90, 1.26) |
| ≥11.08% and <14.78% | 294 (0.74) | 1.05 (0.89, 1.24) | 1.07 (0.90, 1.27) | 1.11 (0.93, 1.32) |
| ≥14.78% | 366 (0.92) | 1.30 (1.12, 1.52) | 1.35 (1.13, 1.62) | 1.41 (1.18, 1.69) |
| ***P*-trend ^b^** |  | < 0.001 | < 0.001 | < 0.001 |
| **Continuous (per 5% energy increase)** |  | 1.11 (1.06, 1.16) | 1.14 (1.08, 1.21) | 1.16 (1.09, 1.22) |

*Abbreviations:* CIs, confidence intervals; HRs, hazard ratios. ^a^ HRs and 95% CIs were calculated by the sub-distribution competing risk model (Fine and Gray’s model). ^b^ Test for trend based on variables containing the median value for each quartile. ^c^ Model 1 was the crude model. ^d^ Model 2 was adjusted for age at recruitment, sex, ethnicity, education level, average total household income before tax, Townsend Deprivation Index, physical activity, alcohol consumption status, smoking status, intakes of protein and fat (percentage of energy intake), and total energy intake. ^e^ Model 3 was adjusted for Model 2 + history of hypertension, diabetes, and cardiovascular diseases, and family history of dementia (free sugar intake or non-free sugar intake were mutually adjusted for each other).

**Supplementary Table 9.** **Subgroup analyses for the associations of non-free sugar and free sugars intakes (% energy intake) with incident** **dementia (n = 158,408) ^a^**

| **Subgroups** | Quartile 1 | Quartile 2 | Quartile 3 | Quartile 4 | Per 5% energy increase | *P* for interaction ^b^ |
| --- | --- | --- | --- | --- | --- | --- |
| **Sex** |  |  |  |  |  |  |
| Men |  |  |  |  |  |  |
| Non-free sugar | Reference | 0.91 (0.74, 1.11) | 0.96 (0.78, 1.19) | 1.22 (0.97, 1.54) | 1.09 (1.01, 1.17) | 0.96 |
| Free sugar | Reference | 1.10 (0.88, 1.38) ^a^ | 1.07 (0.85, 1.34) | 1.40 (1.11, 1.76) | 1.15 (1.07, 1.23) | 0.20 |
| Women |  |  |  |  |  |  |
| Non-free sugar | Reference | 1.49 (1.03, 2.16) | 1.77 (1.24, 2.53) | 1.65 (1.13, 2.39) | 1.10 (1.02, 1.20) |  |
| Free sugar | Reference | 1.00 (0.77, 1.30) | 1.19 (0.92, 1.55) | 1.50 (1.14, 1.98) | 1.19 (1.09, 1.30) |  |
| **Age** |  |  |  |  |  |  |
| ≤ 60 years |  |  |  |  |  |  |
| Non-free sugar | Reference | 1.34 (0.92, 1.94) | 1.66 (1.13, 2.43) | 1.56 (1.01, 2.41) | 1.14 (1.01, 1.29) | 0.21 |
| Free sugar | Reference | 0.91 (0.61, 1.37) | 1.30 (0.88, 1.92) | 1.47 (0.98, 2.20) | 1.23 (1.10, 1.38) | 0.04 |
| > 60 years |  |  |  |  |  |  |
| Non-free sugar | Reference | 1.00 (0.82, 1.22) | 1.14 (0.94, 1.39) | 1.32 (1.07, 1.64) | 1.12 (1.05, 1.19) |  |
| Free sugar | Reference | 1.10 (0.91, 1.32) | 1.09 (0.90, 1.32) | 1.43 (1.18, 1.74) | 1.14 (1.08, 1.22) |  |
| **Body mass index** |  |  |  |  |  |  |
| < 25 kg/m^2^ |  |  |  |  |  |  |
| Non-free sugar | Reference | 1.23 (0.89, 1.70) | 1.34 (0.97, 1.86) | 1.31 (0.93, 1.87) | 1.09 (1.00, 1.20) | 0.21 |
| Free sugar | Reference | 1.21 (0.91, 1.62) | 1.28 (0.96, 1.72) | 1.27 (0.92, 1.75) | 1.10 (1.00, 1.22) | 0.55 |
| ≥ 25 kg/m^2^ |  |  |  |  |  |  |
| Non-free sugar | Reference | 0.93 (0.75, 1.14) | 1.04 (0.85, 1.29) | 1.23 (0.98, 1.54) | 1.09 (1.02, 1.17) |  |
| Free sugar | Reference | 0.99 (0.80, 1.22) | 1.02 (0.82, 1.26) | 1.51 (1.22, 1.86) | 1.19 (1.12, 1.27) |  |
| **Physical activity** |  |  |  |  |  |  |
| < median value |  |  |  |  |  |  |
| Non-free sugar | Reference | 1.12 (0.86, 1.44) | 1.22 (0.94, 1.58) | 1.24 (0.93, 1.66) | 1.06 (0.97, 1.15) | 0.96 |
| Free sugar | Reference | 0.97 (0.76, 1.26) | 1.02 (0.79, 1.32) | 1.27 (0.98, 1.66) | 1.10 (1.02, 1.20) | 0.19 |
| ≥ median value |  |  |  |  |  |  |
| Non-free sugar | Reference | 0.94 (0.74, 1.19) | 1.10 (0.87, 1.39) | 1.29 (1.01, 1.66) | 1.14 (1.06, 1.22) |  |
| Free sugar | Reference | 1.14 (0.90, 1.43) | 1.19 (0.95, 1.50) | 1.57 (1.23, 1.99) | 1.21 (1.13, 1.31) |  |
| **Diabetes** |  |  |  |  |  |  |
| No |  |  |  |  |  |  |
| Non-free sugar | Reference | 1.02 (0.85, 1.24) | 1.16 (0.96, 1.40) | 1.30 (1.06, 1.59) | 1.11 (1.05, 1.18) | 0.10 |
| Free sugar | Reference | 1.04 (0.86, 1.24) | 1.12 (0.93, 1.34) | 1.43 (1.19, 1.73) | 1.17 (1.11, 1.24) | 0.80 |
| Yes |  |  |  |  |  |  |
| Non-free sugar | Reference | 0.96 (0.59, 1.58) | 1.07 (0.65, 1.75) | 1.00 (0.57, 1.73) | 0.98 (0.82, 1.15) |  |
| Free sugar | Reference | 1.22 (0.78, 1.90) | 1.09 (0.67, 1.78) | 1.38 (0.83, 2.31) | 1.10 (0.94, 1.30) |  |
| **Hypertension** |  |  |  |  |  |  |
| No |  |  |  |  |  |  |
| Non-free sugar | Reference | 1.08 (0.76, 1.53) | 1.33 (0.95, 1.87) | 1.44 (0.99, 2.08) | 1.15 (1.04, 1.28) | 0.23 |
| Free sugar | Reference | 1.31 (0.95, 1.79) | 1.19 (0.86, 1.67) | 1.47 (1.04, 2.07) | 1.19 (1.08, 1.33) | 0.97 |
| Yes |  |  |  |  |  |  |
| Non-free sugar | Reference | 1.00 (0.82, 1.23) | 1.09 (0.89, 1.34) | 1.22 (0.98, 1.52) | 1.08 (1.02, 1.16) |  |
| Free sugar | Reference | 0.98 (0.80, 1.20) | 1.09 (0.89, 1.33) | 1.42 (1.16, 1.75) | 1.16 (1.09, 1.23) |  |

^a^ Hazard ratio (95% confidence interval) (all such values). Hazard ratios and 95% confidence intervals were calculated by the cause-specific competing risk model with adjustments for age at recruitment, sex, ethnicity, education level, average total household income before tax, Townsend Deprivation Index, physical activity, alcohol consumption status, smoking status, intakes of protein and fat (percentage of energy intake), total energy intake, history of hypertension, diabetes, and cardiovascular diseases, and family history of dementia, except the stratification factor (free sugar intake or non-free sugar intake were mutually adjusted for each other). ^b^ Test for interaction was calculated by involving the multiplicative interaction term in the fully adjusted model.

**
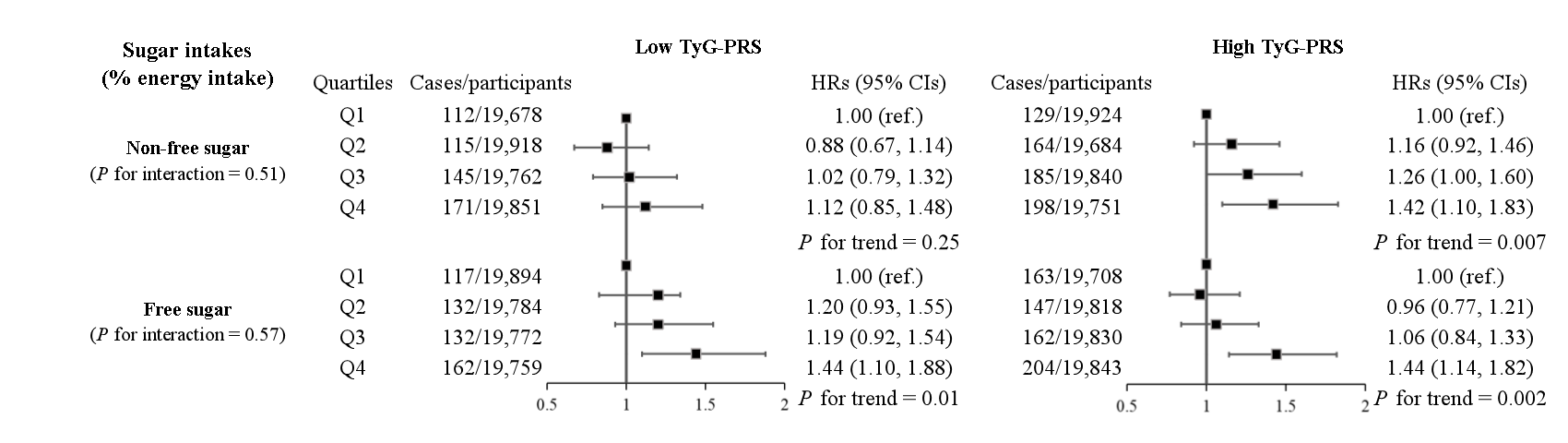
Supplementary Fig 2. Associations of sugar intake with incident dementia according to** **the PRS of TyG (n = 158,408)**

*Abbreviations:* CIs, confidence intervals; HRs, hazard ratios; PRS, polygenic risk score; TyG, triglyceride glucose index.

HRs and 95% CIs were calculated by the cause-specific competing risk model with adjustments for age at recruitment, sex, ethnicity, education level, average total household income before tax, Townsend Deprivation Index, physical activity, alcohol intake status, smoking status, intakes of protein and fat (percentage of energy intake), total energy intake, history of hypertension, diabetes, and cardiovascular diseases, and family history of dementia.


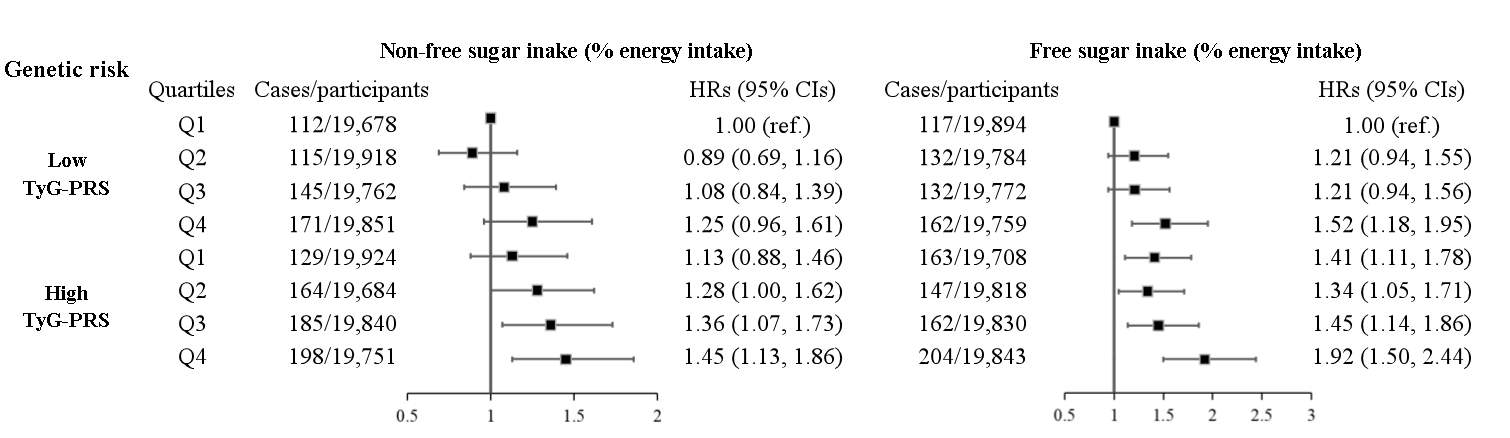


**Supplementary Fig 3. Joint associations of sugar intake and the PRS of TyG with incident dementia (n = 158,408)**

*Abbreviations:* CIs, confidence intervals; HRs, hazard ratios; PRS, polygenic risk score; TyG, triglyceride glucose index.

HRs and 95% CIs were calculated by the cause-specific competing risk model with adjustments for age at recruitment, sex, ethnicity, education level, average total household income before tax, Townsend Deprivation Index, physical activity, alcohol intake status, smoking status, intakes of protein and fat (percentage of energy intake), total energy intake, history of hypertension, diabetes, and cardiovascular diseases, and family history of dementia.

**Supplementary Table 10. Associations between genetic risk of gut microbial and incident dementia (n = 158,408)**

| **Gut microbial** | **HRs (95% CIs)** | ***P* value** | **FDR adjusted-*P*** |
| --- | --- | --- | --- |
| *Bacterodies* | 1.124 (1.004, 1.259) | 0.0423 | 0.0846 |
| *Gordonibacter* | 0.891 (0.796, 0.997) | 0.0443 | 0.0590 |
| *Oscillospira* | 1.122 (1.002, 1.257) | 0.0455 | 0.0455 |
| *Ruminococcaceae UCG-014* | 0.853 (0.762, 0.954) | 0.0056 | 0.0224 |

*Abbreviations:* CIs, confidence intervals; HRs, hazard ratios; FDR, false discovery rate; PRS, polygenic risk score.

HRs and 95% CIs were calculated by the cause-specific competing risk model with adjustments for age at recruitment, sex, ethnicity, education level, average total household income before tax, Townsend Deprivation Index, physical activity, alcohol consumption status, smoking status, intakes of protein and fat (percentage of energy intake), total energy intake, history of hypertension, diabetes, and cardiovascular diseases, family history of dementia,

**Supplementary Table 11. Associations between genetic risk of dementia and incident dementia (n = 158,408)**

| Dementia-PRS | Cases/participants | HRs (95% CIs) | | |
| --- | --- | --- | --- | --- |
|  |  | Model 1 | Model 2 | Model 3 |
| Per standard deviation increase | - | 1.69 (1.61, 1.78) | 1.74 (1.65, 1.82) | 1.71 (1.63, 1.80) |
| <median value | 369/79,204 | 1.00 (Ref) | 1.00 (Ref) | 1.00 (Ref) |
| ≥median value | 850/79,204 | 2.31 (2.04, 2.61) | 2.37 (2.10, 2.68) | 2.30 (2.03, 2.60) |

*Abbreviations:* CIs, confidence intervals; HRs, hazard ratios; PRS, polygenic risk score.

HRs and 95% CIs were calculated by the cause-specific competing risk model with adjustments for age at recruitment, sex, ethnicity, education level, average total household income before tax, Townsend Deprivation Index, physical activity, alcohol consumption status, smoking status, intakes of protein and fat (percentage of energy intake), total energy intake, history of hypertension, diabetes, and cardiovascular diseases, family history of dementia, first 10 genetic principal components, and genotyping batch (free sugar intake or non-free sugar intake were mutually adjusted for each other).


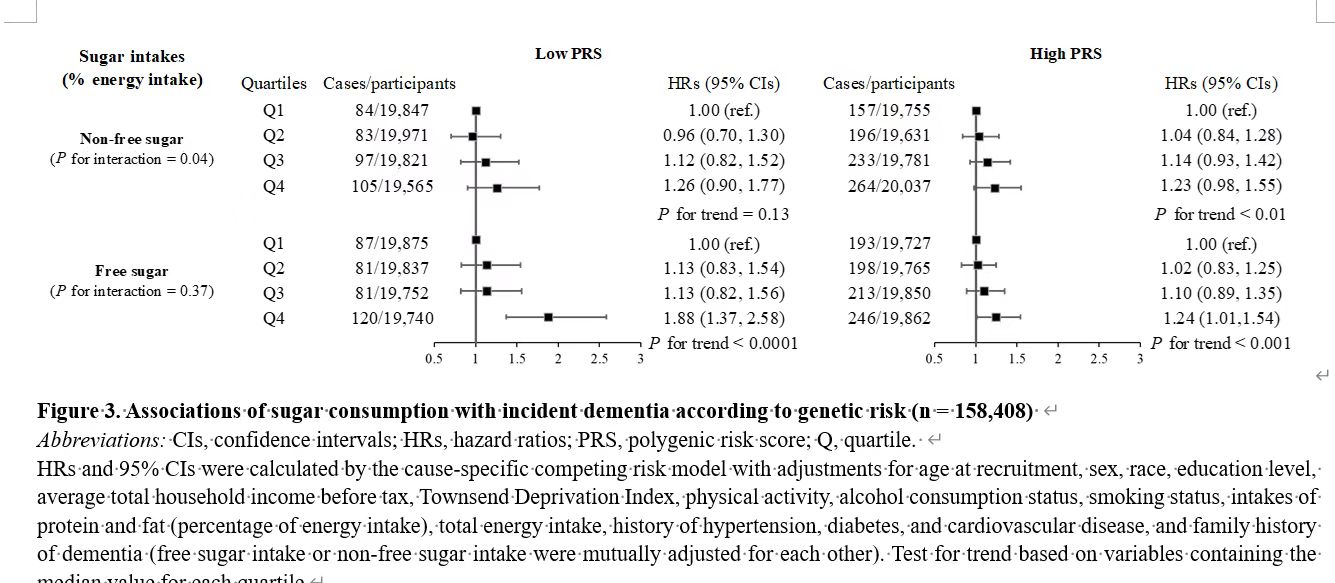


**Supplementary Fig 4. Associations of sugar intake with incident dementia according to genetic risk (n = 158,408)**

*Abbreviations:* CIs, confidence intervals; HRs, hazard ratios; PRS, polygenic risk score; Q, quartile.

HRs and 95% CIs were calculated by the cause-specific competing risk model with adjustments for age at recruitment, sex, race, education level, average total household income before tax, Townsend Deprivation Index, physical activity, alcohol consumption status, smoking status, intakes of protein and fat (percentage of energy intake), total energy intake, history of hypertension, diabetes, and cardiovascular disease, and family history of dementia (free sugar intake or non-free sugar intake were mutually adjusted for each other). Test for trend based on variables containing the median value for each quartile.

**
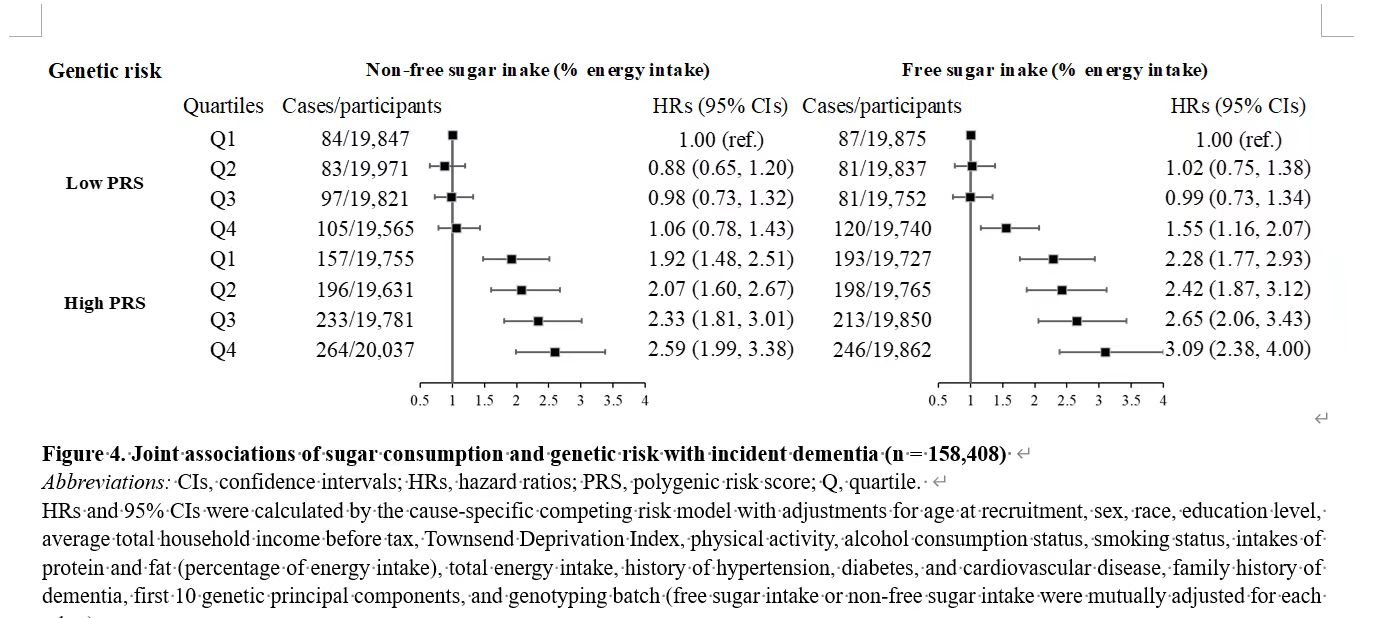
**

**Supplementary Fig 5. Joint associations of sugar intake and genetic risk with incident dementia (n = 158,408)**

*Abbreviations:* CIs, confidence intervals; HRs, hazard ratios; PRS, polygenic risk score; Q, quartile.

HRs and 95% CIs were calculated by the cause-specific competing risk model with adjustments for age at recruitment, sex, race, education level, average total household income before tax, Townsend Deprivation Index, physical activity, alcohol consumption status, smoking status, intakes of protein and fat (percentage of energy intake), total energy intake, history of hypertension, diabetes, and cardiovascular disease, family history of dementia, first 10 genetic principal components, and genotyping batch (free sugar intake or non-free sugar intake were mutually adjusted for each other).
